# Nickel-catalyzed reductive coupling of unactivated alkyl bromides and aliphatic aldehydes†

**DOI:** 10.1039/d1sc03712a

**Published:** 2021-08-10

**Authors:** Cole L. Cruz, John Montgomery

**Affiliations:** Department of Chemistry, University of Michigan 930 North University Avenue Ann Arbor Michigan 48108-1055 USA jmontg@umich.edu

## Abstract

A mild, convenient coupling of aliphatic aldehydes and unactivated alkyl bromides has been developed. The catalytic system features the use of a common Ni(ii) precatalyst and a readily available bioxazoline ligand and affords silyl-protected secondary alcohols. The reaction is operationally simple, utilizing Mn as a stoichiometric reductant, and tolerates a wide range of functional groups. The use of 1,5-hexadiene as an additive is an important reaction parameter that provides significant benefits in yield optimizations. Initial mechanistic experiments support a mechanism featuring an alpha-silyloxy Ni species that undergoes formal oxidative addition to the alkyl bromide *via* a reductive cross-coupling pathway.

## Abstract 2

Aliphatic aldehydes and alkyl bromides are reductively coupled using nickel catalysis. A BiOX ligand and 1,5-hexadiene paired with a silyl chloride and Mn as the terminal reductant are important features of the process.

## Graphical abstract

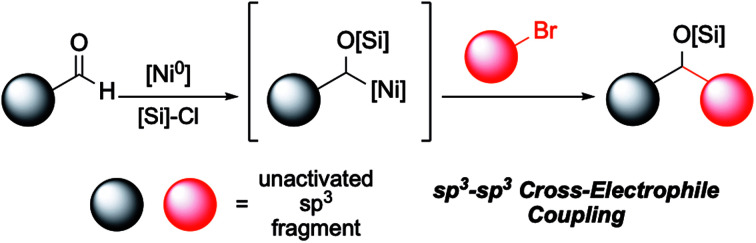

## Introduction

**Scheme 1:**
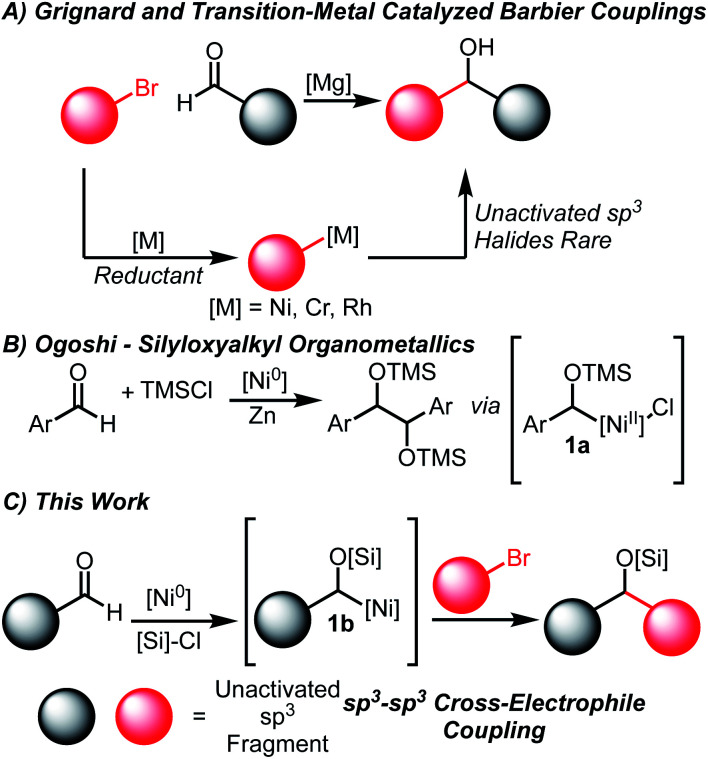
Strategies for forging C–C bonds from aldehydes and halides.

The coupling of carbonyl compounds and carbon-nucleophiles is of broad interest to the chemical community to build molecular complexity. The most ubiquitous methods are the Grignard and related Barbier-type reactions that transform organohalide coupling partners into suitable organometallic nucleophiles (Scheme 1A).^1,2^ However, the need to pre-generate highly reactive organometallic intermediates is undesirable and occasionally non-trivial, especially on small scales relevant for high-throughput experimentation.^3–5^ While Barbier conditions allow for *in situ* generation of organometallic nucleophiles, these are generally restricted to activated allylic or propargylic halides.

An especially appealing strategy for introducing Grignard-type couplings for medicinal chemistry efforts is through transition-metal catalysis. These processes are attractive due to the generation of metalated intermediates of lower nucleophilicity, a higher control of selectivity by tuning the catalytic systems, and ability to be applied on process scales.^6–8^ Net reductive couplings using organohalide feedstocks have been developed using stoichiometric reductants to enable catalyst turnover, obviating the need for pre-generation of the organometallic nucleophile.^9–16^ Among the most common systems are Rh,^10^ Ni^11,14–16^ and Cr^17–24^-catalyzed couplings of aldehydes and organohalides. Importantly, each of these systems are proposed to proceed *via* Grignard-type mechanisms, generating organometallic nucleophiles that undergo formal additions to carbonyl electrophiles.^11,14,20,25^ The vast majority of Ni-catalyzed couplings of this type only tolerate aromatic aldehydes and either aryl, allylic or propargylic halides.^11,13,15,16,18,26,27^

The limitations of existing methods are potentially derived from the properties of the requisite metalated nucleophilic intermediates.^11,14,25^ Aliphatic aldehydes are less suitable coupling partners for the organonickel intermediates generated from oxidative addition of aryl halides, as noted by Weix.^11^ Additionally, controlling the selective activation and heterocoupling of alkyl halides under reductive conditions is a considerable challenge as homocoupling pathways are often favorable.^28–30^

Due to these challenges, a significant gap remains in the development of reductive couplings that join aliphatic aldehydes and unactivated sp^3^ fragments. Cr-catalyzed couplings of alkyl halides (using Co co-catalysis) have been demonstrated however examples are limited.^31^ Alternative coupling partners such as redox-active esters have offered access to similar skeleton frameworks, although similar issues of homocoupling can be encountered.^12,13^ An electrochemical Cr-catalyzed coupling of redox-active esters and aldehydes has been recently demonstrated by Reisman, Blackmond, and Baran.^31^ While the scope of the electrochemical process is exceptionally broad, examples with aliphatic halides and aliphatic aldehydes were not illustrated. Key developments from MacMillan similarly provide a broad array of substrate combinations in additions of various bromides to aldehydes through a method that involves acyl radical additions.^32^ Other selective couplings of alkyl organonickel nucleophiles derived from alkyl halides have been described by Martin, and their use has been focused thus far to trapping with electrophiles such as CO_2_ and isocyanates.^33–36^

In order to circumvent these challenges in reductive aldehyde/alkyl halide couplings, we envisioned developing a method to activate aldehydes in an Umpolung fashion.^37–40^ A key report from Ogoshi detailing the formation of α-silyloxy Ni(ii) complexes of type **1a** (Scheme 1B) from Ni(0) sources, aldehydes and silyl chlorides provided inspiration towards this goal.^41^ Indeed, Mackenzie^42^ and Weix^43^ have shown that similar allyl complexes derived from Ni(0), Michael acceptors and silyl chlorides undergo C–C coupling with appropriate organohalide sources. We hypothesized that the generation of this α-silyloxy Ni-complex **1b** (Scheme 1C) could be leveraged with known Ni-catalyzed cross-electrophile coupling reactions of unactivated sp^3^ halides to afford silyl-protected ether products.^30^ This outcome represents an important gap in the field and is made complex by the myriad of potential homocoupling, isomerization, and elimination pathways available to the substrates.^44^

## Results and discussion

An initial screen of ligands commonly employed in reductive cross-couplings revealed BiOX as a uniquely promising candidate for selective coupling (Table 1, see ESI† for more optimization details). In all reactions, competitive formation of the corresponding enol ether **3** and silyl ether **4** in addition to homocoupling of the alkyl bromide was observed. Upon further screening we found that olefin additives could minimize the formation of these side products, affording better mass balance and higher yields, with excellent reactivity observed when using 1,5-hexadiene. Olefin additives have been demonstrated to have a significant impact on the efficiency of catalytic reactions in other contexts,^45^ and the 1,5-hexadiene additive here proved to be important in yield optimization and side product minimization. Further optimization revealed that when using 0.75 equivalents of this additive, in addition to 0.50 equivalents of NaI, excellent yields of the desired product were obtained.

**Table 1:** Optimization of reaction parameters^a^^,^^b^

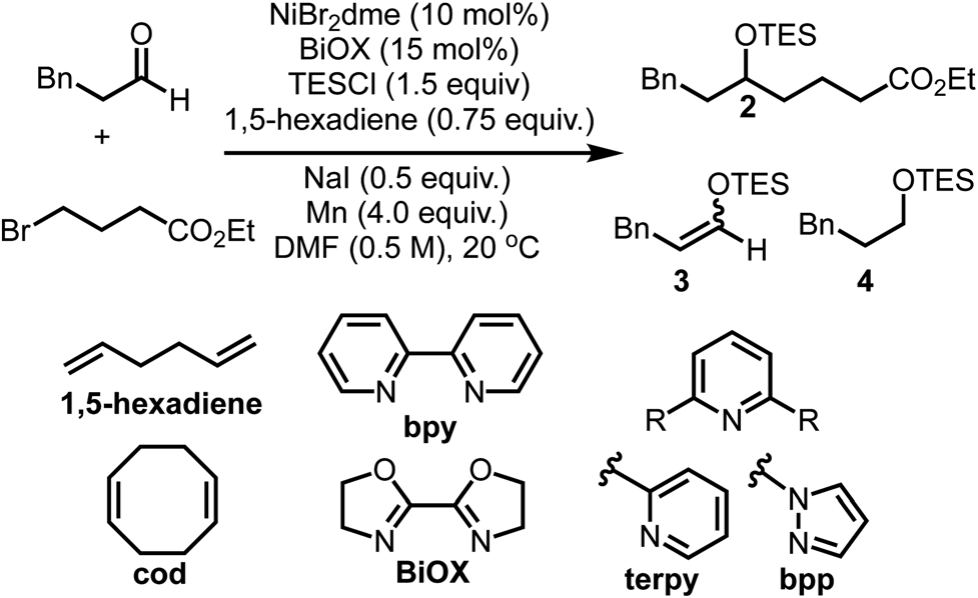				
Entry	Deviation from standard conditions	**2**^c^ (%)	**3**^c^ (%)	**4**^c^ (%)
1	None	93	<5	<5
2	No NaI	76	10	10
3	No diene	46	20	20
4	1-Octene instead of 1,5-hexadiene	76	10	12
5	cod instead of 1,5-hexadiene	59	19	23
6	*E*-stilbene instead of 1,5-hexadiene	21	20	20
7	Duroquinone instead of 1,5-hexadiene	6	<5	<5
8	bpy instead of BiOX	15	24	21
9	terpy instead of BiOX	0	40	30
10	bpp instead of BiOX	47	27	20
11	PPh_3_ instead of BiOX	0	50	50
12	Zn instead of Mn	85	ND	0
13	TMSC1 instead of TESCI	46	ND	ND
14	No Ni	0	<5	0
15	No ligand	0	10	0
16	No Mn	0	10	0

a ND = not determined.

b Unless otherwise stated, reactions were run on a 0.2 mmol scale at 20 °C with respective to the aldehyde substrate and 2.0 equiv. of alkyl bromide.

c Yields were determined either by NMR *vs.* mesitylene or by GC-FID with 1,3,5-trimethoxybenzene as an internal standard.

With optimized conditions in hand, we explored the reaction scope. Varying the aldehyde component revealed that the reaction tolerated steric variation around the reactive site while enabling chemoselective activation of the aldehyde (Table 2). Benzyl ethers and straight-chain aldehydes were shown to couple effectively. Substrates with β-branching, such as citronellal (**7**) and isovaleraldehyde (**8**), delivered the desired products in good yield. Aldehydes bearing protected amines such as carbamates were well tolerated, showing no activation or cleavage of the Boc group in **9**. α-Branched aldehydes showed comparable reactivity to linear substrates (**10–13**), however, aldehydes bearing α-quaternary centers did not afford meaningful amounts of coupled product.

**Table 2:** Aldehyde reaction scope^a^

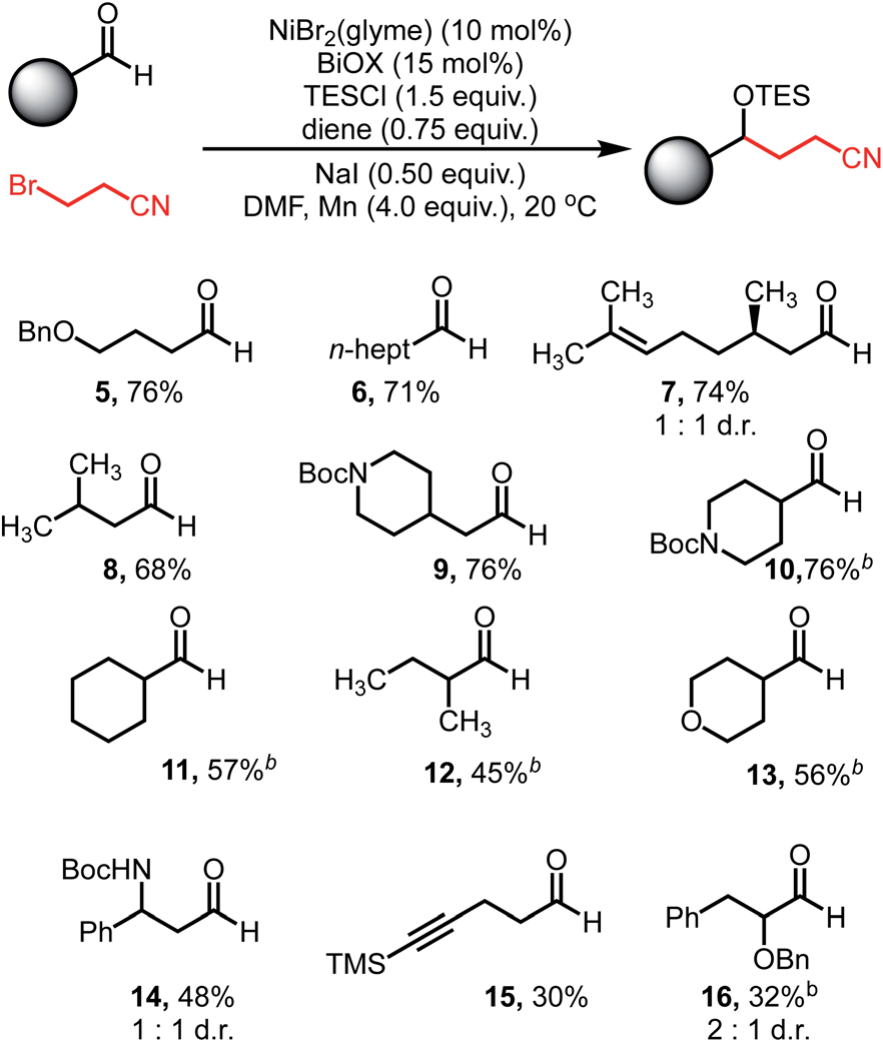

a General reaction conditions on a 0.5 mmol scale: aldehyde (1.0 equiv.), 3-bromopropionitrile (2.0 equiv.), NiBr_2_(glyme) (10 mol%), BiOX (15 mol%), TESCl (1.5 equiv.), 1,5-hexadiene (0.75 equiv.), NaI (0.5 equiv.), Mn (4.0 equiv.), DMF (1.0 mL), 20 °C. Yields refer to isolated yields.

b Reaction run without NaI.

Alkyne functionality was also tolerated, affording modest yields of the carbonyl coupling (**15**). Additionally, decreased reactivity was observed when heteroatom functionality is located at the α-position (**16**). The reaction displayed good reactivity for aldehydes, however ketones afforded minimal amounts of coupled products. Additionally, aryl aldehydes are incompatible under these conditions due to efficient, undesired pinacol homocoupling (see ESI†).^41^

Exploration of the scope of the alkyl bromide coupling partner displayed similarly broad functional group compatibility (Table 3). As a general note, while we found that the inclusion of NaI was beneficial during the initial optimization with ethyl 4-bromobutyrate, in some cases the inclusion of NaI led to decreased yields of the cross-coupled products. We attribute this to enhanced reactivity of certain alkyl iodide substrates generated from *in situ* halogen exchange reactions (see ESI† for more discussion).

**Table 3:** Alkyl bromide reaction scope^a^

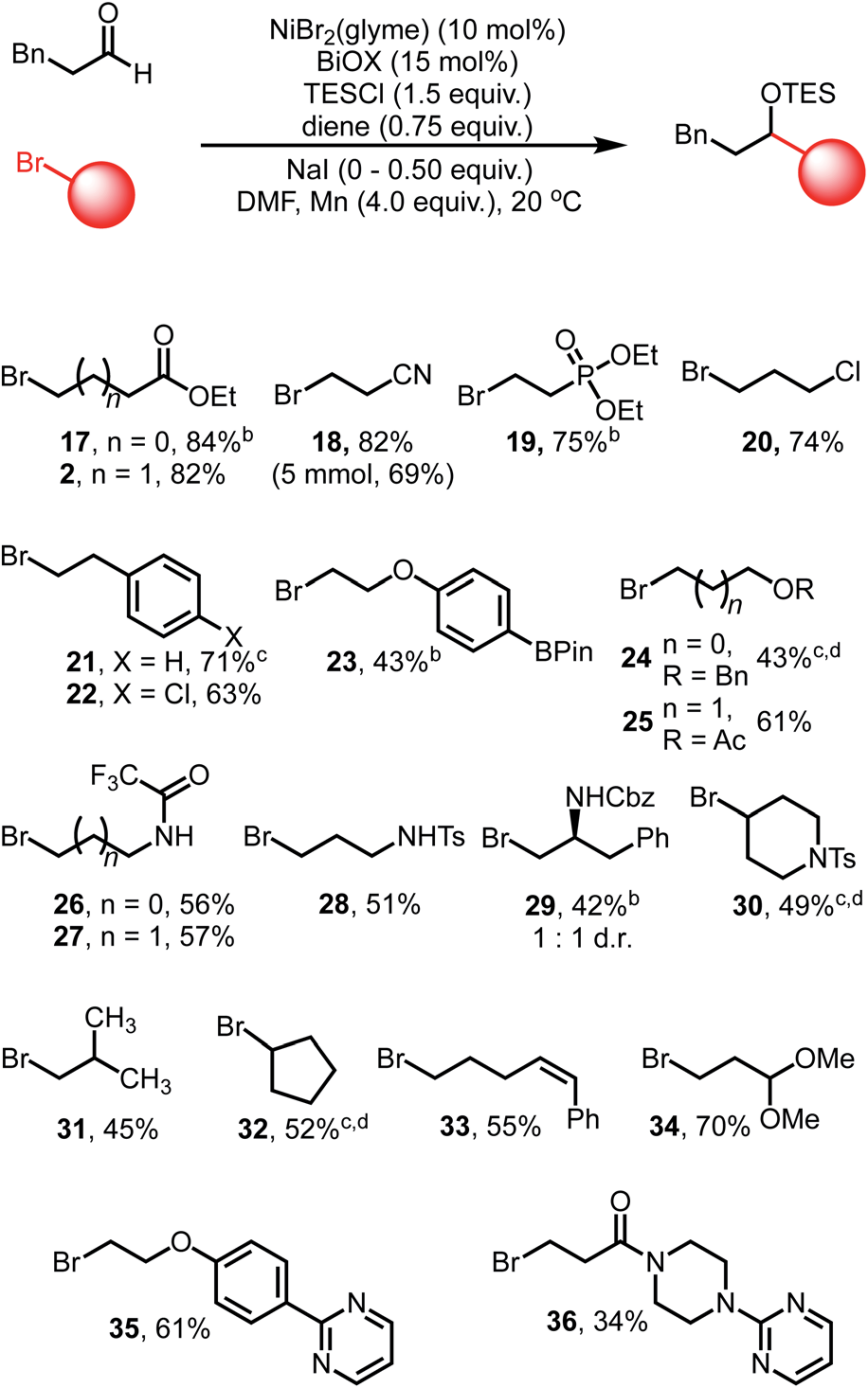

a General reaction conditions: dihydrocinnamaldehyde (1.0 equiv.), alkyl bromide (1.0–2.0 equiv.), NiBr_2_(glyme) (10 mol%), BiOX (15 mol%), TESCl (1.5 equiv.), 1,5-hexadiene (0.75 equiv.), NaI (0.5 equiv.), Mn (4.0 equiv.), DMF (1.0 mL), 20 °C. Yields refer to isolated yields.

b Reaction run without NaI.

c Reaction run with 1.0 equiv. LiBr instead of NaI.

d Product isolated as the corresponding alcohol following TBAF deprotection.

Esters, nitriles, and phosphonate esters (**2**, **17–19**) are well tolerated and afford high yields of coupled products, highlighting the mild nature of these conditions. Alkyl and aryl chlorides (**20**, **22**) are not activated under these conditions, nor are boronate esters (**23**), offering further sites for derivatization *via* other cross-coupling systems. Ethers (**24**) and acetates (**25**) are coupled in excellent yields, allowing for polyol structures to be constructed. Protected amine functional groups, such as trifluoroacetamides (**26–27**), sulfonamides (**28**) and carbamates (**29**) are also tolerated. Sterically encumbered bromides such as protected piperidine derivatives (**30**), isobutyl bromide (**31**) and cycloalkyl bromides (**32**) are competent coupling partners, albeit displaying diminished yields. Secondary bromides such as **30** and **32** could be coupled in moderate yields when LiBr is used in place of NaI.^10,46,47^

We also found that more activated olefins are not susceptible to competitive reactions under these conditions as evidenced by substrate **33**. The geometric configuration of the olefin is maintained throughout the reaction, suggesting that chain-walking processes related to activation of the bromide are slow in this catalytic system. Potentially Lewis acidic sensitive groups such as acetals (**34**) are also tolerated. Finally, heterocyclic functionality is tolerated, as evidenced by the clean participation of pyrimidine structures **35** and **36**. Efficient coupling was also demonstrated on a 5.0 mmol scale, obtaining coupled product **18** in slightly diminished yield (69%).

**Scheme 2:**
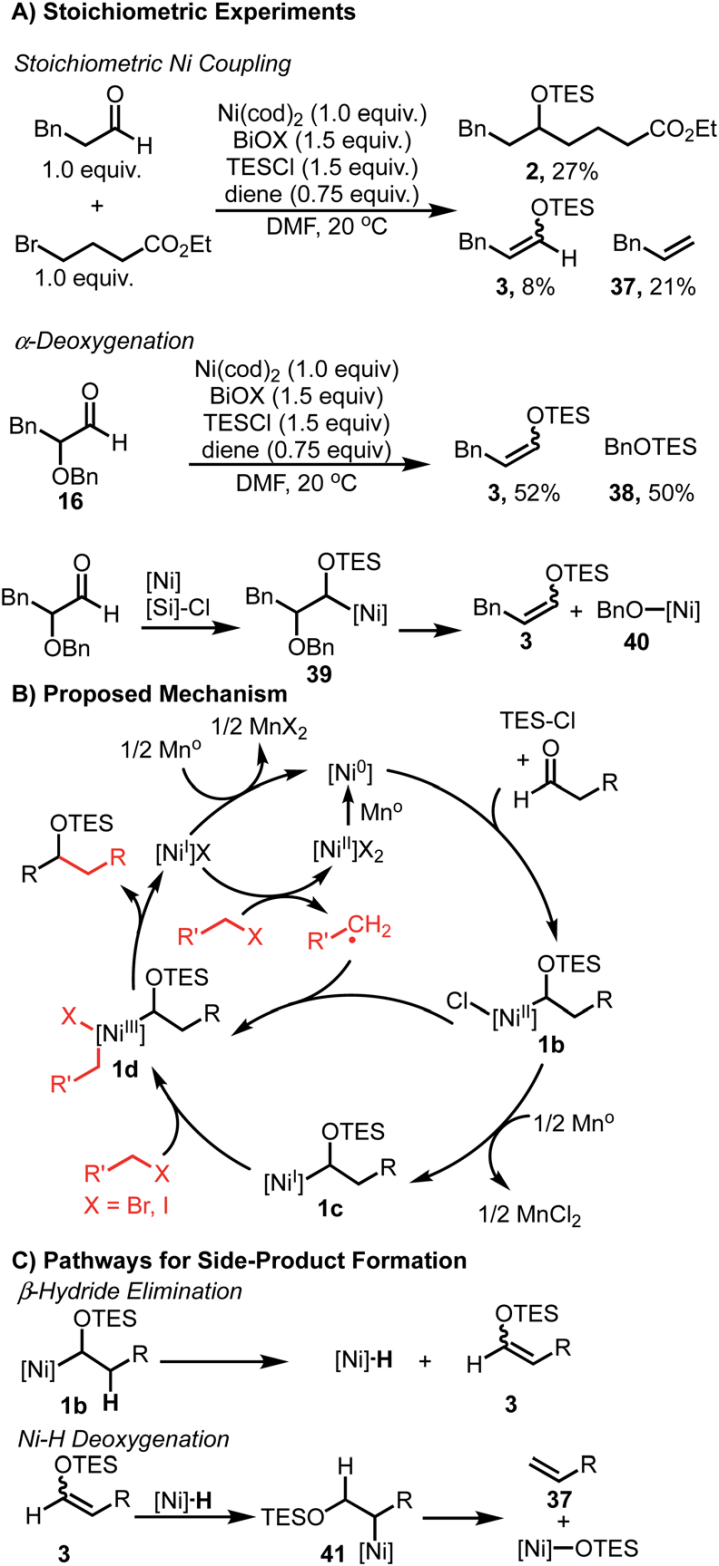
Mechanistic considerations and catalytic cycle.

We anticipated that the formation of enol ether **3** and silyl ether **4** (Table 1) may shed light on key intermediates along the catalytic pathway. Stoichiometric experiments revealed similar product distributions observed under the catalytic conditions. When using Ni(cod)_2_ and equivalent amounts of aldehyde and bromide, a 27% yield of the coupled product **2** was observed (Scheme 2A). In addition, undesired products **3** and **37** were also observed in 8% and 21% yield, respectively. Additionally, when α-oxy aldehyde **16** was subjected to a stoichiometric reaction, enol ether **3** was observed in 52% yield in addition to 50% yield of silyl ether **40**. The formation of both of these species would be consistent with a sequence outlined (Scheme 2A) wherein intermediate **40** undergoes β-oxy elimination to furnish **3**, and the resulting nickel alkoxide **40** undergoes silylation with TESCl to furnish **38**.

The observation of **3** and **37** in both the stoichiometric experiments as well as the catalytic coupling conditions suggest a common mechanism for their formation. Control experiments confirmed that the aldehyde is only consumed when both Ni(ii) salt and reductant are present (see ESI† for details). This observation is consistent with activation of the aldehyde *via* interaction with a low-valent Ni species. Additionally, when TESCl is omitted, complete consumption of the aldehyde is observed to give a complex mixture, suggesting that the presence of TESCl leads to stabilization of a key intermediate and allows for productive coupling. Importantly, only trace enolization is observed when Ni is omitted from these reactions, ruling out TESCl-mediated enolization as a pathway to generate HCl, which could react to form a nickel hydride species.

Taken together, these results provide preliminary evidence for the formation of an α-silyloxy(alkyl)nickel species. A catalytic cycle combining these observations is shown (Scheme 2B). First, following reduction of the Ni(ii) pre-catalyst, Ni(0) undergoes complexation with the aldehyde substrate and TESCl to furnish intermediate **1b**. This intermediate can then engage the alkyl halide coupling partner *via* either a radical-chain process as noted by Weix,^30^ or through a sequential reduction – oxidative addition pathway involving Ni(i) intermediate **1c**, to produce **1d**.^48,49^ Regardless of the exact nature of this step, the resulting dialkyl Ni(iii) intermediate **1d** can then undergo reductive elimination to furnish the coupled product and a Ni(i) salt. This Ni(i) salt is either reduced by Mn to regenerate the active Ni(0) catalyst or activates another equivalent of alkyl halide to propagate the radical chain process. A stoichiometric reaction between aldehyde and alkyl bromide, in the absence of TESCl, afforded no cross-coupled product and only bromide homocoupling was observed. This suggests that alternative mechanisms where an alkyl-Ni nucleophile forms and undergoes a formal migratory insertion with the aldehyde substrate to form the C–C bond are unlikely.^33^ While α-silyloxy(alkyl)nickel intermediates **1b** and/or **1c** rationalize the observed reactivity, the involvement of α-silyloxy(alkyl)chlorides or the corresponding ketyl radicals cannot be firmly excluded as possible intermediates in the catalytic cycle.^40,50^

The formation of **3**, **4** and **37** can be explained *via* undesired side-pathways of silyloxy intermediates **1b** or **1c** (Scheme 2C). β-Hydride elimination from any silyloxy(alkyl)nickel intermediates would result in the formation of derivatives of **3** and a Ni–H species. Reinsertion of this Ni–H species into **3** can afford a new alkyl-Ni intermediate **41** poised to undergo β-oxy elimination to furnish allylbenzene **37** and a Ni-alkoxide. Alternatively, the Ni–H species can reduce an equivalent of aldehyde to ultimately produce **4**.

As noted in the optimization studies, alkene additives play a beneficial role, although not strictly required for productive catalysis (Table 1, entry 3). Variation in alkene structure is tolerated, with beneficial effects generally being observed and 1,5-hexadiene being optimal among those examined (Table 1, entries 1, 4–7). This diene is desirable in that it forms stable complexes with nickel as illustrated in related complexes by Pörschke^51^ and Hazari.^52^ Furthermore, prior work from our lab illustrated significant benefits of 1,5-hexadiene compared with 1,5-cyclooctadiene by avoiding chain-walking pathways in C–H activation processes.^53,54^ The origin of the beneficial effects in aldehyde/alkyl halide couplings may involve increasing catalyst stability,^51–56^ suppressing substrate degradation pathways (Scheme 2), or promoting slow catalytic steps.^45^ Studies are underway to better understand these aspects.

## Conclusions

In conclusion, an efficient reductive coupling of aliphatic aldehydes and unactivated alkyl halides has been developed. The process addresses a key limitation of prior methods, specifically the ability to introduce unactivated C(sp^3^) groups in both the aldehyde and alkyl halide reaction components. Additionally, the process tolerates a wide range of functionality and is amenable to scaleup. The reaction utilizes an air-stable Ni(ii) pre-catalyst and easily synthesized BiOX ligand. Preliminary mechanistic experiments suggest a novel mechanism proceeding through a silyloxy(alkyl)nickel intermediate that effectively engages a free radical derived from an alkyl halide. Efforts to expand the scope of coupling partners and devise more efficient catalytic systems are currently underway.

## Author contributions

CLC performed the experimental work and CCL and JM jointly conceived the project, analyzed data, and wrote the manuscript.

## Conflicts of interest

There are no conflicts to declare.

## Supplementary Material

SC-012-D1SC03712A-s001
