# Supplementary material for: Nickel-catalyzed reductive coupling of unactivated alkyl bromides and aliphatic aldehydes
Source: Chem Sci. 2021 Aug 10;12(36):11995–2000. doi: 10.1039/d1sc03712a (PMC8457385; doi:10.1039/d1sc03712a)
Supplement: SC-012-D1SC03712A-s001 [file SC-012-D1SC03712A-s001.pdf]

Electronic Supplementary Material (ESI) for Chemical Science.
This journal is © The Royal Society of Chemistry 2021

Supporting Information:

Nickel-Catalyzed Reductive Coupling of Unactivated Alkyl Bromides and Aliphatic Aldehydes

Cole L. Cruz, and John Montgomery*

Department of Chemistry, University of Michigan, 930 N. University Ave., Ann Arbor, MI 48108-1055, USA

E-mail: jmontg@umich.edu

Table of Contents

I. General Information……………………………………………………………….......S2
II. Optimization Screens…………………………………… …………….…….…..S3-S10
III. Synthesis of Starting Materials…………………………. …..……………….……S11-S14
IV. Mechanistic Studies and Discussions……...……………………………..….......…S15-S23
V. Reductive Coupling Procedure and Characterization Data…...………………...…S24-S35
VI. References……………………………………………………………...…….S36-S37
VII. NMR Spectra………………………………… ……....……………………S38-S74

I. General Information

Methods and Materials: Proton and carbon magnetic resonance spectra (^{1}H NMR and ^{13}C NMR) were recorded on either Varian vnmrs 500 or Varian vnmr 600(^{1}H NMR at 400 MHz, 600 MHz and ^{13}C NMR at 100, 150 MHz) spectrometer with solvent resonance as the internal standard (^{1}H NMR: $CDCl_3$ at 7.26 ppm; ^{13}C NMR: $CDCl_3$ at 77.0 ppm). ^{1}H NMR data are reported as follows: chemical shift, multiplicity (s = singlet, d = doublet, t = triplet, dd = doublet of doublets, dt = doublet of triplets, td = triplet of doublets, m = multiplet, br = broad singlet, bm = broad multiplet), coupling constants (Hz), and integration. Flash chromatography was performed using SiliaFlash P60 silica gel (40-63 μm) purchased from Silicycle. All solvents were dried by passage over activated alumina columns immediately prior to use unless otherwise noted. Bromides for which syntheses are not described within were bought from commercial sources and either used as received or eluted through a 1" basic alumina plug immediately prior to use. Aldehydes for which syntheses are not described within were bought from commercial sources and either used as received or purified via distillation from $CaCl_2$. $NiBr_2$(dme) (97%) was purchased from Strem and used as received within a nitrogen-filled glovebox. Chlorotriethylsilane was purchased from Oakwood Chemicals or TCI Chemicals and distilled from calcium hydride then stored in a sealed Schlenck tube. Manganese (325 mesh) as well as Manganese (325 mesh, 99.99% trace metals basis) were both purchased from Sigma-Aldrich and used as received within a nitrogen-filled glovebox or on the benchtop. BiOX was synthesized according to a reported procedure.[1]

II. Optimization Screens

General Procedure for Reaction Optimization: Optimization reactions were setup on a 0.2 mmol scale with respect to limiting reagent (generally the aldehyde component). An oven-dried, 8 mL culture tube equipped with a stir bar was brought into a nitrogen-filled glovebox. The Ni precatalyst, ligand, manganese and any other solid additives were charged to the tube, which was then sealed with a PTFE-coated septum screw-cap. The tube with solid reagents was then removed from the glovebox and placed under a nitrogen atmosphere. The vial was then charged with 0.4 mL DMF and any liquid additives, if relevant. The mixture was then stirred at 1100 rpm for 10 min, after which point the aldehyde, silane and bromide were added sequentially, neat. The nitrogen needle was then removed and the vial caps were sealed with electrical tape and allowed to stir for 16 h. After, reactions were quenched with sat. aqueous NH_4Cl and diluted with ether. The organic layer was filtered through a short silica gel plug and then analyzed via GCMS or NMR using either 1,3,5-trimethoxybenzene or mesitylene, respectively, as an internal standard.

Initial Ligand Screen:

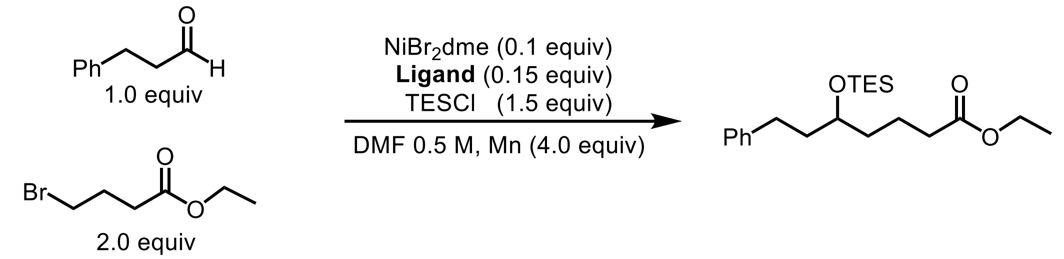

Ligands evaluated:

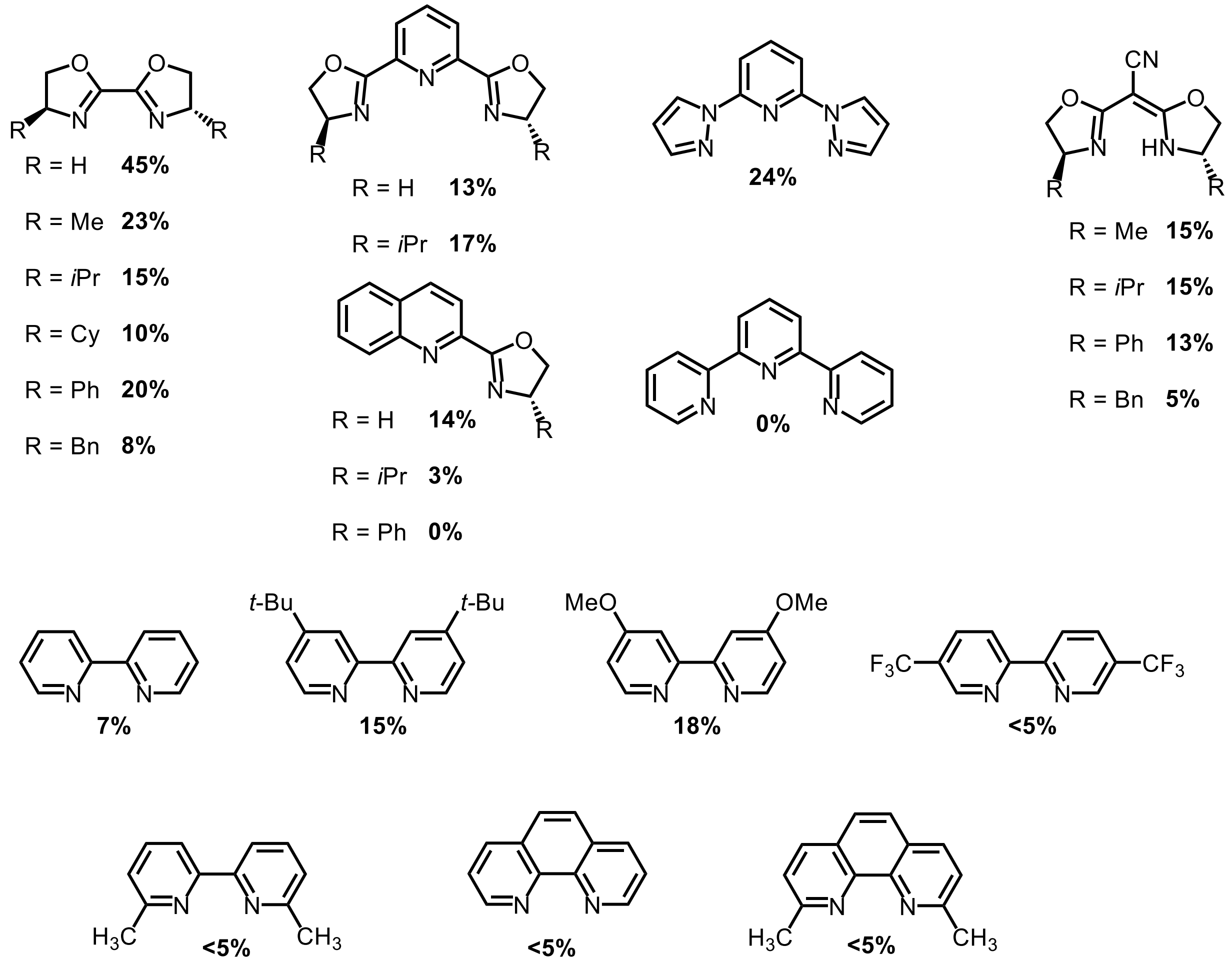

Initial Silane Screen

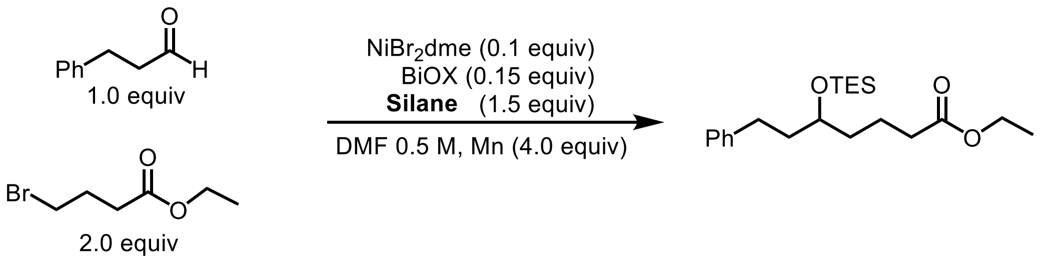

**Silane**	Yield (%)
TESCl	46
TIPSCl	10%
TMSBr	<5%
TMSOTf	<5%

1,5-Hexadiene Equivalents

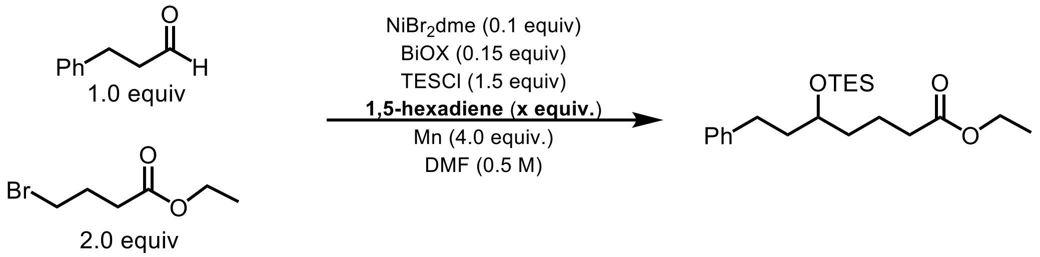

**1,5-hexadiene equiv.**	Yield (%)
0	46
0.25	53
0.50	67
0.75	76
1.0	76

Additive Screen

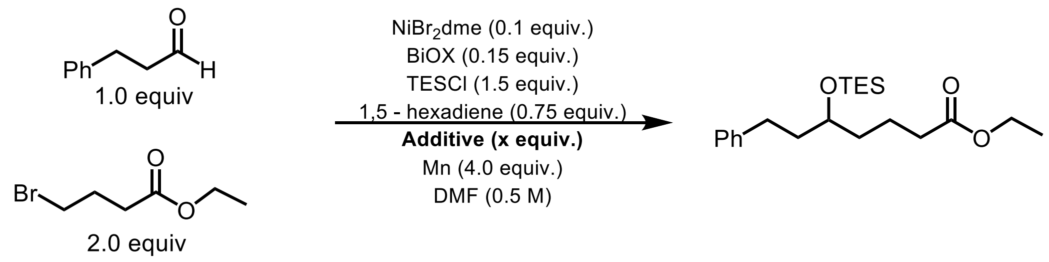

**Additive (equiv.)**	Yield (%)
None	76
LiBr (0.5 equiv.)	61
$N(Bu)_4Br$ (0.5 equiv.)	60
$N(Bu)_4I$ (0.5 equiv.)	76
NaI (0.5 equiv.)	93

Extended Final Optimization Table

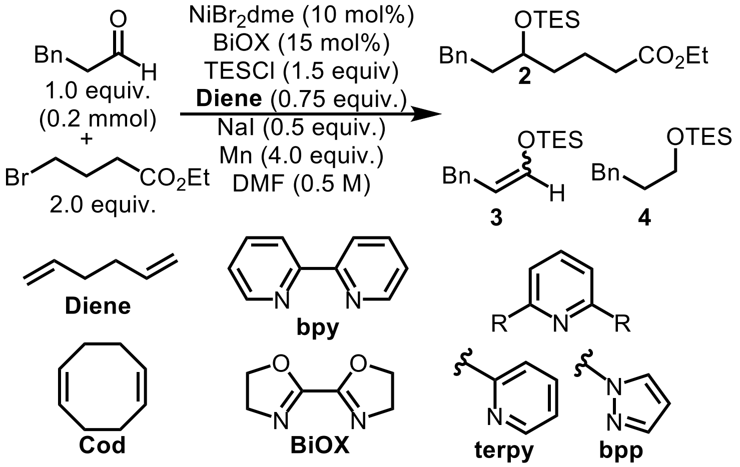

Entry	Deviation	2 (%)	3 (%)	4 (%)
1	*none*	93	<5	<5
2	*no NaI*	76	10	10
3	*no diene*	46	20	20
4	*1-octene instead of* ***diene***	76	10	12
5	***Cod*** *instead of* ***diene***	59	19	23
6	**bpy** instead of **BiOX**	15	24	21
7	**terpy** instead of **BiOX**	0	40	30
8	***bpp*** *instead of* ***BiOX***	47	27	20
9	*PPh_3 instead of* ***BiOX***	0	50	50
10	*Zn instead of Mn*	85	ND	0
11	*TMSCl instead of TESCl*	46	ND	ND
12	*TIPSCl instead of TESCl*	31	ND	ND
13	*$PhMe_2SiCl$ instead of TESCl*	22	ND	ND
14	*No Ni*	0	<5	0
15	*No Ligand*	0	10	0
16	*No Mn*	0	10	0
17	***TDAE*** *instead of Mn*	0	ND	ND
18	*15 mol% Ni, 18 mol% BiOX*[a]	68	ND	ND
19	*3.0 equiv. Bromide*	64	ND	ND
20	*1.0 equiv. Bromide*	91	ND	ND
21	*1.0 equiv. Bromide, 0.5 mmol*	80	ND	ND
22	*none, time = 2 hours*	64	ND	ND
23	*none, time = 4 hours*	96	ND	ND
24	*$NiCl_2$dme instead of $NiBr_2$dme*	68	10	12
25	*iPr-BiOX instead of BiOX*	10	ND	ND
26	*Bn-BiOX instead of BiOX*	13	ND	ND

ND = Not Determined

Differing Effects of Sodium Iodide Addition

As noted in the text of the manuscript and optimization tables here, the addition of NaI was found to increase yield when coupling with ethyl 4-bromobutyrate and many other cases. However, in some cases this additive lead to decreased yields such as when coupling with ethyl 3-bromopropionate or diethyl 2-bromoethylphosphonate. We attribute this to enhanced HalEx rates for these substrates due to increased electrophilicity of the alkyl bromide induced by nearby electropositive groups such as esters and phosphonates.[2]. These increased rates result in a larger reservoir of the more reactive alkyl iodide which may then undergo competitive homocoupling at faster rates. This is in line with our initial observations when substituting alkyl iodides for the alkyl bromide substrates that result in lower yields.

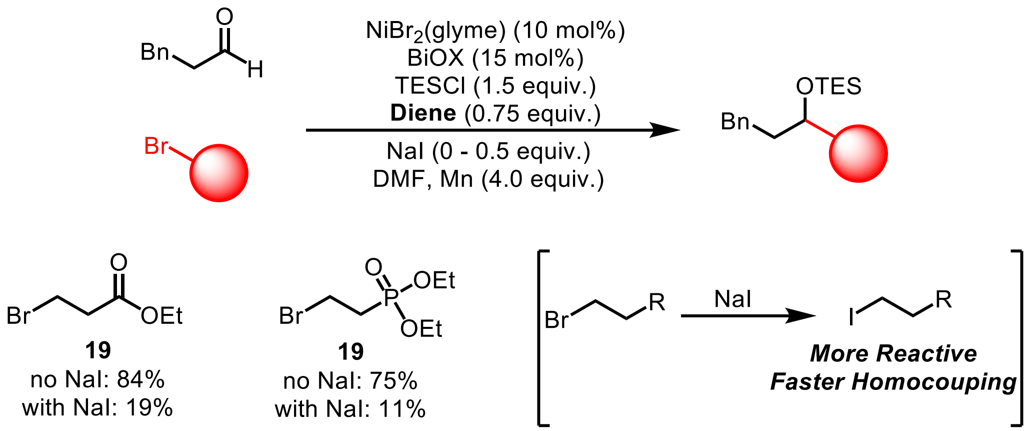

Unsuccessful Substrates

Aryl Aldehydes

Aryl aldehydes were found to be incompatible substrates and did not afford appreciable amounts of heterocoupled products. Instead, under all conditions tested the aryl aldehydes preferentially underwent homocoupling to afford the pinacol-type products as reported by Ogoshi.[3]

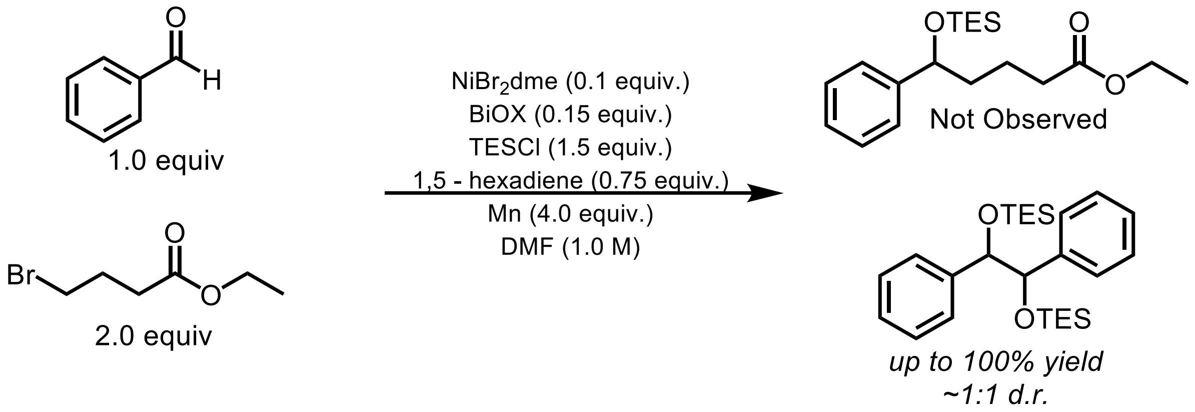

Substrates with Bronsted Basic Moieties

Certain substrates were not tolerated under these reaction conditions. Specifically, substrates (both aldehyde or bromide coupling partners) that also contained functional groups capable of acting as Bronsted bases afforded little to no desired product. For example, piperazine-containing bromide **A** did not afford appreciable amounts of cross-coupled product. Instead, enol ether **3** was the major product. We attribute this to a facile base-mediated enolization of the aldehyde substrate. Additionally, pyridine-containing substrates **B** and **C** did not afford cross-coupled product.

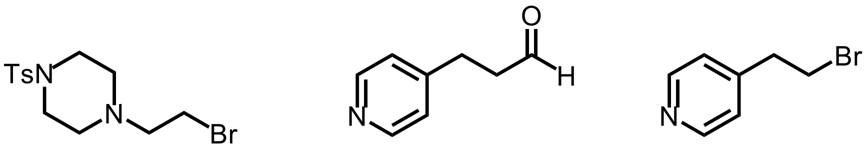

A **B** **C**

In general, due to the presence of enolizable positions on the aldehyde substrates and the silyl chloride reagent, mild-to-moderately basic functional groups are not well tolerated under these conditions. Unprotected amines and basic heterocycles are likely to be limitations as a result of this, as seen above. However, factors that attenuate the basicity of these groups appear to allow for successful reactivity such as in **36** or less-basic heterocycles like pyrimidines as seen in **35**.

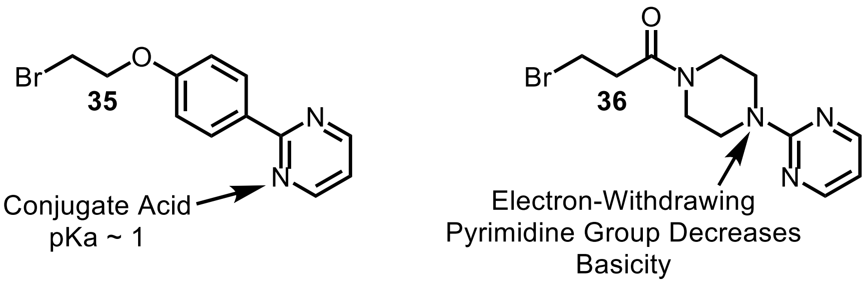

Ketone Substrates

In addition to the aldehyde substrates shown in **Scheme 2**, a number of aliphatic ketone substrates were also subjected to the reaction conditions however no cross-coupled products were observed.

Representative Unsuccessful Ketone Substrates

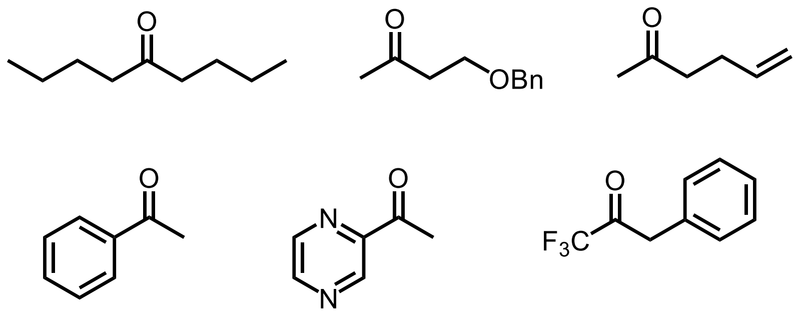

III. Synthesis of Starting Materials

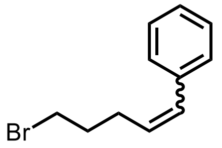

(5-bromopent-1-en-1-yl)benzene (S1): Synthesized by a known procedure from benzaldehyde and (4-bromobutyl)triphenylphosphonium bromide.[4] After column chromatography the product was obtained as a mixture of *E* / *Z* isomers (8 : 92) determined by relative integrations of olefinic protons. Designations of respective signals are given where possible.

^{1}H NMR (500 MHz, $CDCl_3$) δ 7.39 – 7.32 (m, 2H), 7.29 – 7.19 (m, 3H), 6.49 (d, *J* = 11.5 Hz, 1H, *Z*-isomer), 6.46 (m, 1H, *E*-isomer) 6.17 (dt, *J* = 15.8, 7.0 Hz, *E*-isomer), 5.63 (dt, *J* = 11.6, 7.3 Hz, 1H, *Z*-isomer), 3.47 (t, *J* = 6.7 Hz, 2H, *E*-isomer), 3.43 (t, *J* = 6.8 Hz, 2H, *Z*-isomer), 2.49 (m, *J* = 7.4, 1.8 Hz, 2H, *Z*-isomer), 2.39 (m, *J* = 7.1, 1.4 Hz, 2H, *E*-isomer), 2.09 – 1.96 (m, 2H); **^{13}C NMR** (126 MHz, $CDCl_3$) δ 137.2, 130.5, 130.2, 130.1, 128.6, 128.2, 126.7, 33.0, 32.9, 27.2; **HRMS (CI+):** *m/z* calculated for [M+]: 224.0201; found: 224.0192

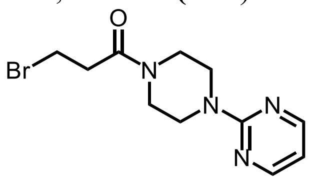

3-bromo-1-(4-(pyrimidin-2-yl)piperazin-1-yl)propan-1-one (S2): Adapted from a known procedure via acylation of 3-bromopropionyl chloride.[5] To a flame-dried 100 mL round-bottom flask was added 2-(piperazin-1-yl)pyrimidine (10.0 mmol, 1.0 equiv.), DMAP (5.0 mmol, 0.5 equiv.) and 20 mL THF. The flask was cooled in an ice-water bath and then 3-bromopropionyl chloride (11.0 mmol, 1.1 equiv.) was added, neat, over 5 min resulting in the formation of a white precipitate. The mixture was then allowed to stir for 30 min in the ice-water bath after which point the reaction was quenched w/ sat. sodium bicarbonate solution. The mixture was extracted 3x w/ 25 mL DCM and then the combined organics were washed successively with water and Brine, then dried over sodium sulfate. The organics were filtered and concentrated and the resulting solid was triturated from hexanes to afford the product as a white solid (887 mg, 30% yield). (*Note:* We found that extended reaction times beyond 30 minutes resulted in significant formation of the corresponding alkyl chloride that could not be separated from the desired bromide).

^{1}H NMR (500 MHz, $CDCl_3$) δ 8.33 (d, *J* = 4.7 Hz, 2H), 6.55 (t, *J* = 4.7 Hz, 2H), 3.90 – 3.86 (m, 2H), 3.84 (dd, *J* = 6.6, 4.0 Hz, 2H), 3.72 (dd, *J* = 6.6, 4.0 Hz, 2H), 3.68 (t, *J* = 7.1 Hz, 2H), 3.60 – 3.49 (m, 2H), 2.97 (t, *J* = 7.1 Hz, 2H).; **^{13}C NMR** (126 MHz, $CDCl_3$) δ 168.68, 161.47, 157.78, 110.56, 45.24, 43.67, 43.48, 41.56, 36.37, 27.15; **HRMS (ESI+):** *m/z* calculated for [M+H]: 299.0507; found: 299.0496

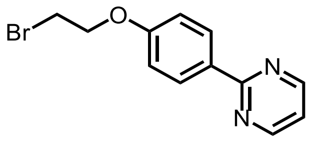

2-(4-(2-bromoethoxy)phenyl)pyrimidine (S3): Synthesized via alkylation of 4-(pyrimidin-2-yl)phenol. A 25 mL flame-dried round bottom flask was charged with 4-(pyrimidin-2-yl)phenol[6] (2.0 mmol, 1.0 equiv.), K_2CO_3 (5.0 mmol, 2.5 equiv.) and DMF (3.0 mL). Then, 1,2-dibromoethane (40.0 mmol, 20.0 equiv.) was added, neat, in one portion and the resulting mixture heated to 70 °C and stirred for 16 h. After, the reaction was cooled and diluted with 10 mL water and 10 mL EtOAc. The layers were separated and the aqueous was then washed twice more with 10 mL EtOAc. The combined organics were then washed with Brine, dried over sodium sulfate, filtered and concentrated. The residue was purified via silica gel column chromatography to afford the product as a white solid (200 mg, 36% yield).

^{1}H NMR (500 MHz, $CDCl_3$) δ 8.76 (d, J = 4.8 Hz, 2H), 8.40 (d, J = 8.9 Hz, 2H), 7.13 (4.8 Hz, 1H), 7.01 (d, J = 8.9 Hz, 2H), 4.37 (t, J = 6.3 Hz, 2H), 3.67 (t, J = 6.3 Hz, 2H); **^{13}C NMR** (126 MHz, $CDCl_3$) δ 164.3, 160.3, 157.1, 130.9, 129.8, 118.4, 114.6, 67.8, 28.8; **HRMS (ESI+):** *m/z* calculated for [M+H]: 279.0133 found: 279.0157

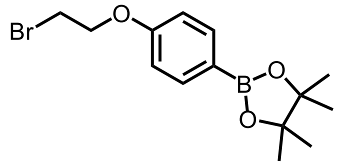

2-(4-(2-bromoethoxy)phenyl)-4,4,5,5-tetramethyl-1,3,2-dioxaborolane (S4): Synthesized in a similar fashion to **S3** via alkylation of 4-(4,4,5,5-tetramethyl-1,3,2-dioxaborolan-2-yl)phenol. **S4** was obtained as a white solid (464 mg, 16%).

^{1}H NMR (500 MHz, $CDCl_3$) δ 7.75 (d, J = 8.6 Hz, 2H), 6.90 (d, J = 8.7 Hz, 2H), 4.32 (t, J = 6.4 Hz, 2H), 3.64 (t, J = 6.4 Hz, 2H), 1.33 (s, 12H); **^{13}C NMR** (126 MHz, $CDCl_3$) δ 160.6, 136.6, 113.9, 83.6, 67.5, 28.9, 24.8; **IR** (thin film): 2360, 1642, 1529, 1393, 1287, 1204, 1159, 1053 cm^{-1}; **HRMS (ESI-):** Under these ionization conditions, the pinacol ester was observed to hydrolyze to the corresponding boronic acid and the formate adduct could be observed. *m/z* calculated for [M-C_6H_{12}][-CO_2H]: 288.9883 found: 288.9888

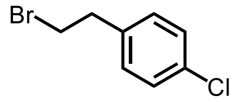

1-(2-bromoethyl)-4-chlorobenzene: Synthesized by a reported procedure from 4-chlorophenethyl alcohol.[7] Spectral properties matched previous reports.

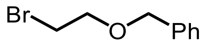

((2-bromoethoxy)methyl)benzene: Synthesized by a reported procedure from 2-(benzyloxy)ethanol.[8] Spectral properties matched previous reports.

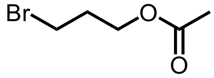

3-bromopropyl acetate: Synthesized by a reported procedure from 3-bromo-1-propanol.[9] Spectral properties matched previous reports.

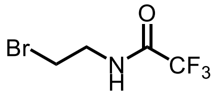

N-(2-bromoethyl)-2,2,2-trifluoroacetamide: Synthesized by a reported procedure from 2-bromoethylamine hydrobromide.[10] Spectral properties matched previous reports.

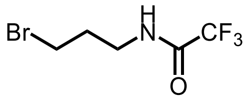

N-(3-bromopropyl)-2,2,2-trifluoroacetamide: Synthesized by a reported procedure from 3-bromopropylamine hydrobromide.[11] Spectral properties matched previous reports.

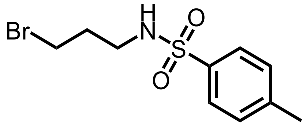

N-(3-bromopropyl)-4-methylbenzenesulfonamide: Synthesized by a reported procedure from 3-bromopropylamine hydrobromide.[12] Spectral properties matched previous reports.

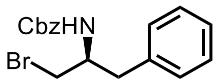

benzyl (S)-(1-bromo-3-phenylpropan-2-yl)carbamate: Synthesized by a reported procedure from Z-L-phenylalaninol.[13] Spectral properties matched previous reports.

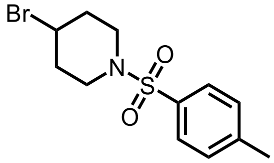

4-bromo-1-tosylpiperidine: Synthesized by a reported procedure from 1-tosylpiperidin-4-ol.[14] Spectral properties matched previous reports.

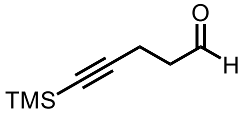

5-(trimethylsilyl)pent-4-ynal: Synthesized by a reported procedure from 5-(trimethylsilyl)pent-4-yn-1-ol.[15] Spectral properties matched previous reports.

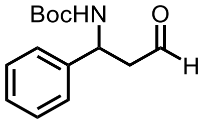

tert-butyl (3-oxo-1-phenylpropyl)carbamate: Synthesized by a reported procedure via oxidation of *tert*-butyl (3-hydroxy-1-phenylpropyl)carbamate.[16] Spectral properties matched previous reports.

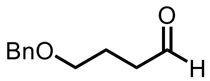

4-(benzyloxy)butanal: Synthesized by a reported procedure via oxidation of 4-(benzyloxy)butan-1-ol.[17] Spectral properties matched previous reports.

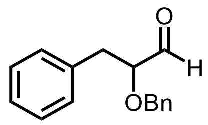

2-(benzyloxy)-3-phenylpropanal: Synthesized by a reported procedure via dithiane deprotection of 2-(1-(benzyloxy)-2-phenylethyl)-1,3-dithiane.[18] Spectral properties matched previous reports.

^{1}H NMR (**400 MHz**, Chloroform-*d*) δ 9.69 (d, *J* = 1.9 Hz, 1H), 7.35 – 7.08 (m, 10H), 4.60 (d, *J* = 11.8 Hz, 1H), 4.48 (d, *J* = 11.8 Hz, 1H), 4.00 – 3.95 (m, 1H), 3.04 (dd, *J* = 14.1, 4.7 Hz, 1H), 2.94 (dd, *J* = 14.2, 8.5 Hz, 1H)

IV. Mechanistic Studies and Considerations

Investigating Possible Formation of Organomanganese Species

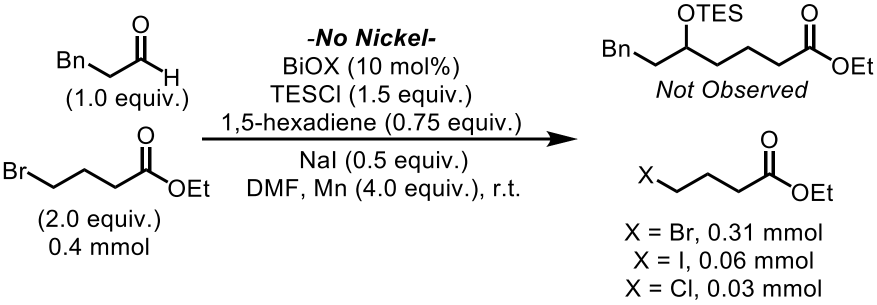

A control experiment was performed where no Ni source was added to the reaction and no heterocoupled product was observed. Additionally, the mass-balance of the initial ethyl 4-bromobutyrate was found to be completely conserved, though spread amongst different halogenated species (from in-situ HalEx reactions[2]). While the generation of organomanganese species directly from highly activated Mn^0 and alkyl halides is known[19], these results suggest that when using these manganese sources this is not an operable pathway under these reaction conditions. The observation of some alkyl chloride species is interesting and suggests that the TESCl also undergoes partial HalEx exchange to other silyl-halide species. It's worth noting that when monitoring the reaction via GCMS under standard coupling procedures the alkyl iodide cannot be observed suggesting that its consumption is more rapid than the corresponding bromide, contributing to various reaction dynamics (see **Differing Effects of Sodium Iodide Addition** above).

Exploring Aldehyde-Degradation Pathways

In all reactions throughout optimization, various amounts of aldehyde degradation products were observed in varying amounts depending on condition, especially when olefin additives such as 1,5-hexadiene were omitted. The predominant species identified were the enol ether species **3**, silyl ether **4** as well as allylbenzene **37**.

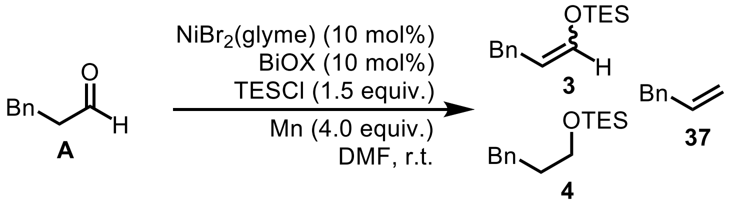

Deviation	A (%)	3 (%)	4 (%)	37 (%)
none	<5	30	20	11
No Ni	>95	<5	0	0
No Mn	90	10	0	0
No TESCl	<5	ND	ND	ND

ND = Not Detected

These results suggested that the enolization process is catalyzed by a low-valent Ni species. Additionally, during optimization reactions the enol ether was observed to form predominately as the *Z*-isomer (~4 : 1 *Z* : *E*). When **3** was synthesized using standard conditions (triethylamine in DMF, see below), the enol ether was isolated with a slight preference for the *E*-isomer (~1 : 2 *Z* : *E*).

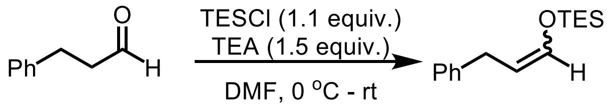

Triethyl((3-phenylprop-1-en-1-yl)oxy)silane (3): A flame-dried 50 mL round-bottom flask was charged with dihydrocinnamaldehyde (5.0 mmol, 1.0 equiv.) and DMF (10 mL). The flask was cooled in an ice-water bath and then triethylamine (7.5 mmol, 1.5 equiv.) was added and the solution was stirred for 5 minutes. Then, the chlorotriethylsilane (5.5 mmol, 1.1 equiv.) was added and the mixture stirred for 3 hours. The reaction was then diluted with DCM and 10 mL saturated NH_4Cl and the layers were separated. The aqueous was extracted twice with 10 mL DCM and then the combined organics were washed with Brine, dried over sodium sulfate, filtered and concentrated. The residue was then purified via silica gel column chromatography to afford **3** as a clear oil in a 2 : 1 ratio of *E* : *Z* isomers. Spectral data matched a previous report.[20]

^{1}H NMR (**500 MHz**, Chloroform-*d*) δ 7.30 – 7.16 (m, 5H), 6.36 (d, *J* = 11.9 Hz, 1H, *E*-isomer), 6.33 (d, *J* = 7.3 Hz, 1H, *Z*-isomer), 5.17 (dt, *J* = 11.9 Hz, 7.5 Hz 1H, *E*-isomer), 4.67 (td, *J* = 7.3 Hz, 5.7 Hz), 3.46 (d, *J* = 5.7 Hz, 2H, *Z*-isomer), 3.24 (d, *J* = 5.7 Hz, 2H, *E*-isomer) (m, 1H), 3.04

(dd, J = 14.1, 4.7 Hz, 1H), 2.94 (dd, J = 14.2, 8.5 Hz, 1H), 1.02 – 0.96 (m, 9H), 0.72 – 0.66 (m, 6H)

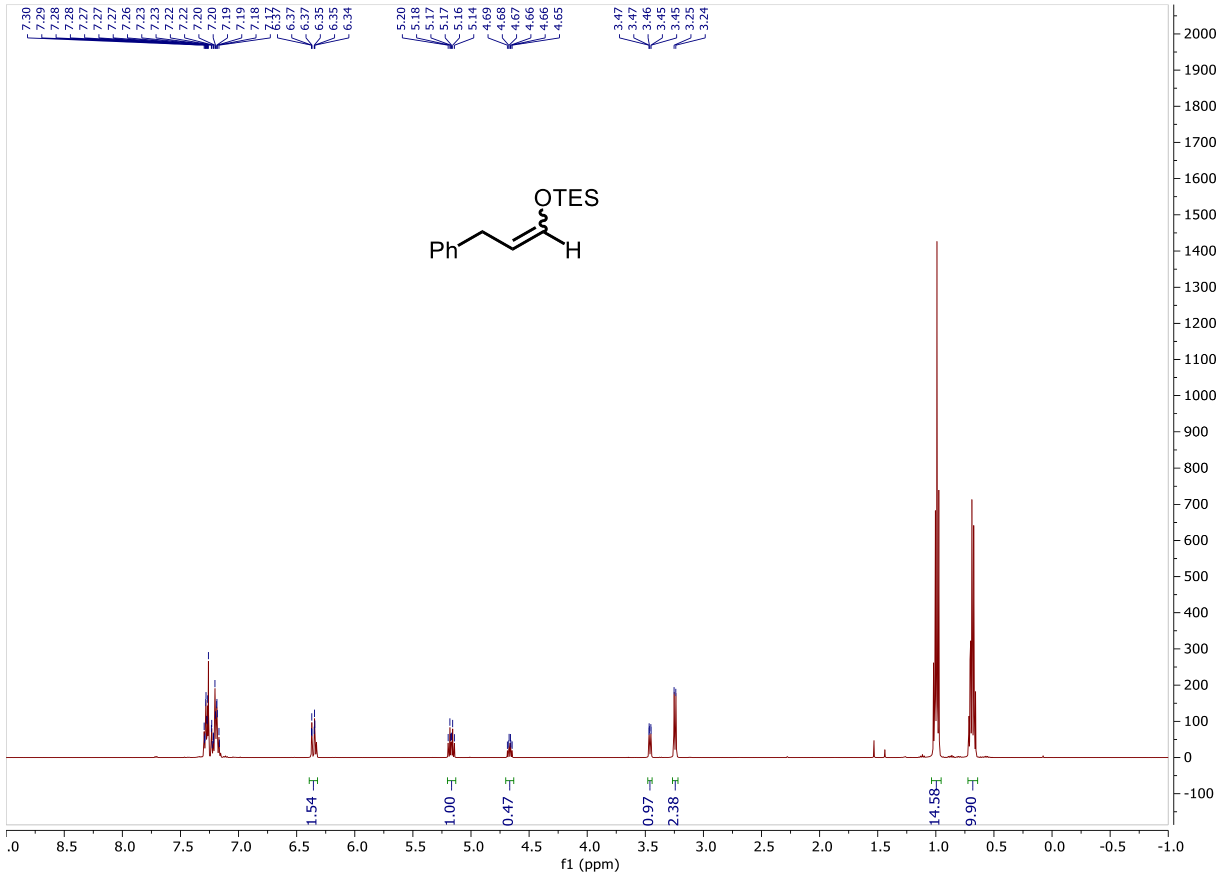

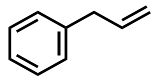

Allylbenzene: A sample of commercially available allylbenzene (Sigma-Aldrich) was used as a reference sample for NMR and GCMS validation. **^{1}H NMR** (400 MHz, Chloroform-*d*) δ 7.38 – 7.27 (m, 2H), 7.28 – 7.19 (m, 3H), 5.98 (ddt, *J* = 16.9, 10.2, 6.7 Hz, 1H), 5.19 – 4.87 (m, 2H), 3.40 (d, *J* = 6.7 Hz, 1H).

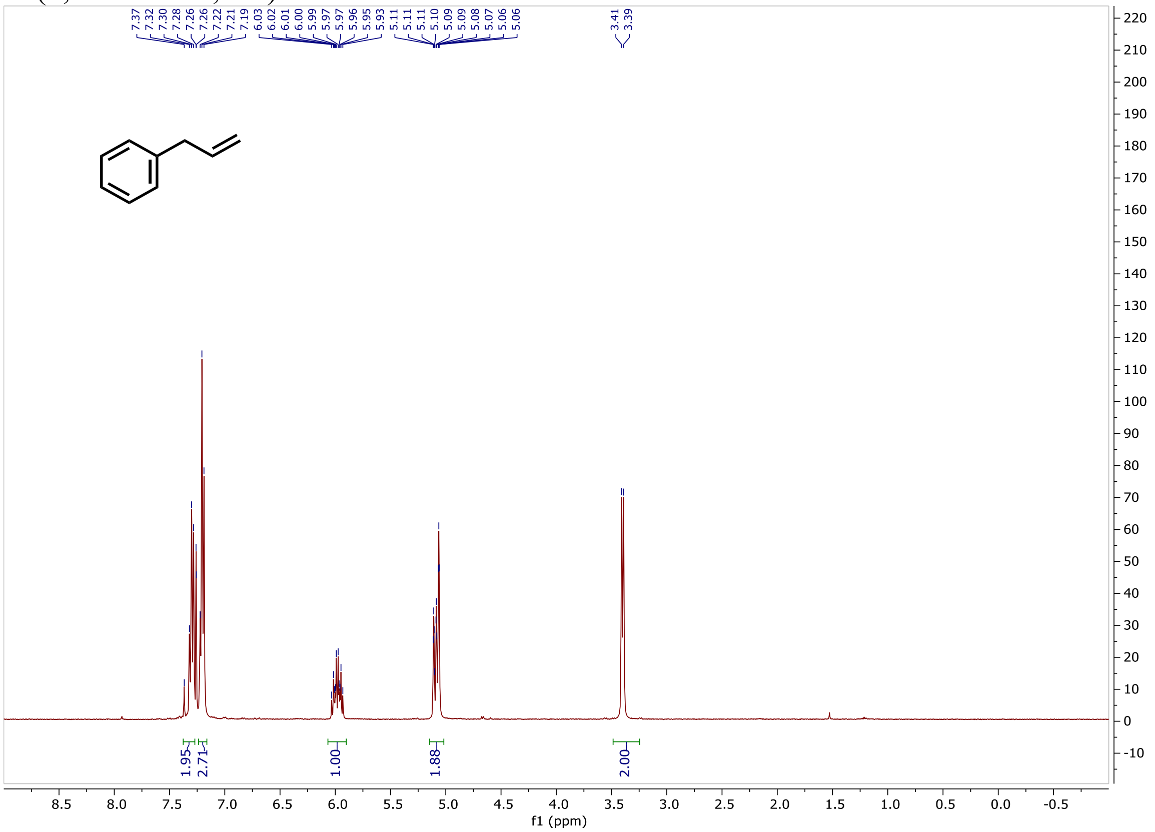

General Procedure for Stoichiometric Experiments: In a nitrogen-filled glovebox, a dried 8 mL culture tube equipped with a stir bar was charged w/ $Ni(cod)_2$ (0.2 mmol, 1.0 equiv.) and BiOX (0.3 mmol, 1.5 equiv.). The tube was sealed with a screw-cap with a Teflon-coated septum and removed from the glovebox then placed under a nitrogen atmosphere. Then, DMF (4 mL) was added followed by 1,5-hexadiene (0.15 mmol, 0.75 equiv.) and the mixture was stirred for 15 minutes to afford a dark red mixture. After stirring, dihydrocinnamaldehyde (0.2 mmol, 1.0 equiv.) was added, followed by TESCl (0.3 mmol, 1.5 equiv.) and finally ethyl 4-bromobutyrate (1-2 equiv.), if applicable. The reactions were stirred for 4-16 hours (most reactions generally turned a green-yellow color upon the end of the reaction) and then poured into a saturated aqueous solution of NH_4Cl. The aqueous layer was extracted 3x with 10 mL 1:1 ether / pentane solution and the combined organics were washed with Brine, dried over sodium sulfate, filtered and concentrated. The crude material was dissolved in a small amount of $CDCl_3$ and Mesitylene (0.2 mmol, 1.0 equiv.) was added as an internal standard and the mixtures analyzed via ^1H NMR.

Stoichiometric Reductive Coupling Reaction:

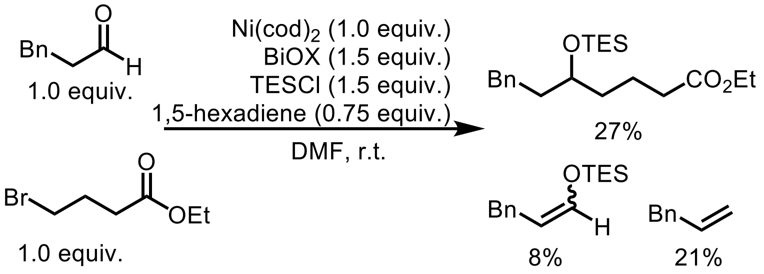

The above reaction was carried out according to the general procedure. The reaction afforded 27% yield of the desired coupling product, 8% yield of the enol ether product (as determined by integration of the alpha-olefin protons at 6.31 – 6.35 ppm) and 21% of allyl benzene (5.97 ppm) along with 22% of remaining aldehyde and an undetermined amount of homocoupled bromide. This stoichiometric experiment demonstrates both the competency of Ni to mediate the formation of all species generated under the catalytic conditions and also rules out the requirement of Mn for product formation, suggesting that the formation of an alkyl-manganese species is not necessary for product formation.

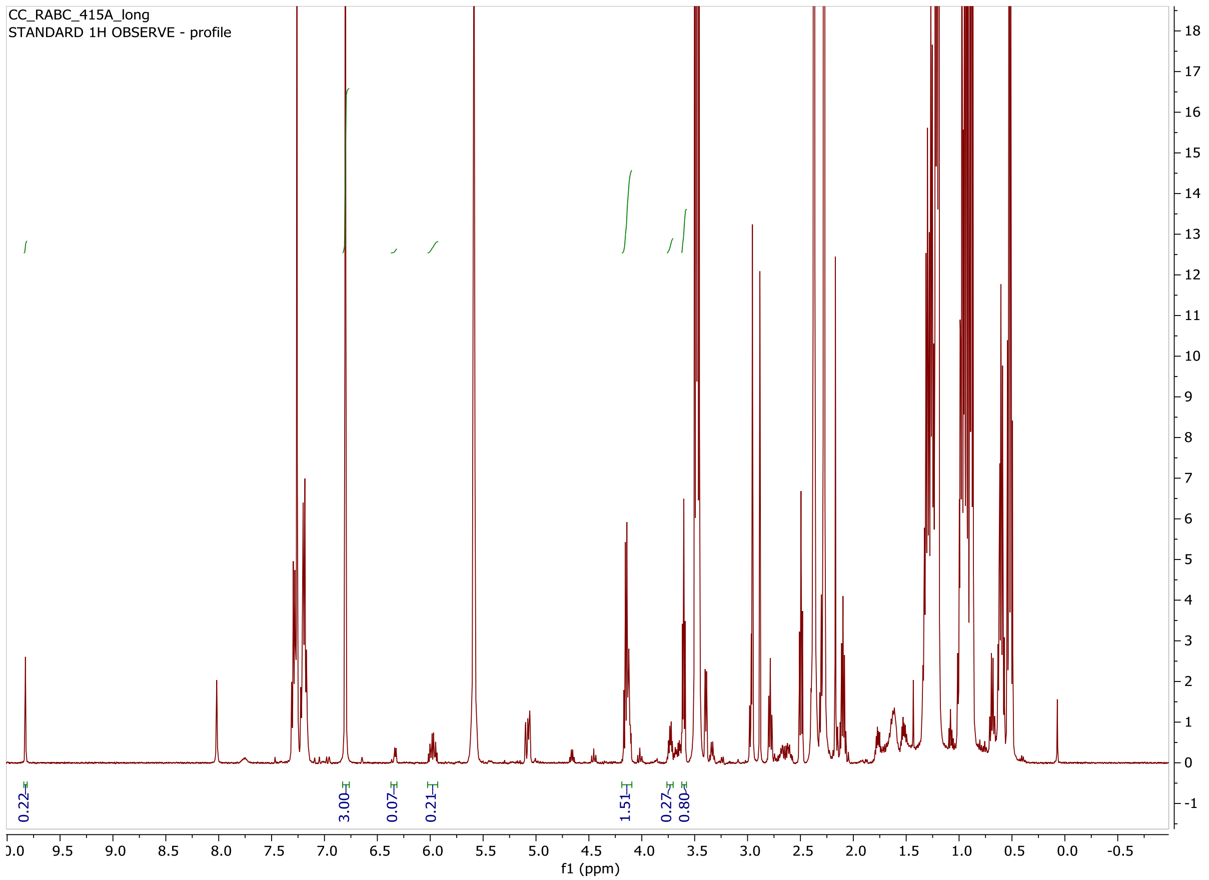

Stoichiometric Reductive Coupling Reaction – No TESCl:

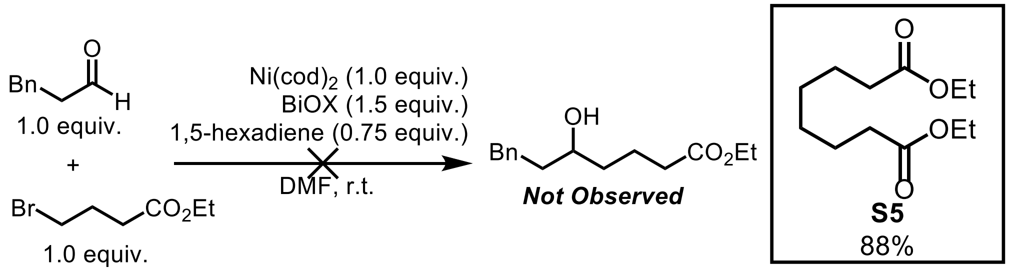

The above reaction was carried out by modifying the general procedure and omitting the TESCl. The reaction did not afford any products resulting from C-C heterocoupling of the two components (detectable by either ^{1}H NMR or GCMS). Instead, the major species obtained at the end of the reaction were residual aldehyde substrate (55% remaining) and the product resulting from homocoupling of the alkyl bromide **S5** (88% yield). The remaining mass balance of the aldehyde is distributed among aldol self-condensation products and unidentified species. These results are inconsistent with a mechanism wherein an alkyl-nickel nucleophile is generated from oxidative addition of Ni(0) to the alkyl bromide and then undergoes C-C bond formation via nucleophilic addition to the aldehyde substrate.

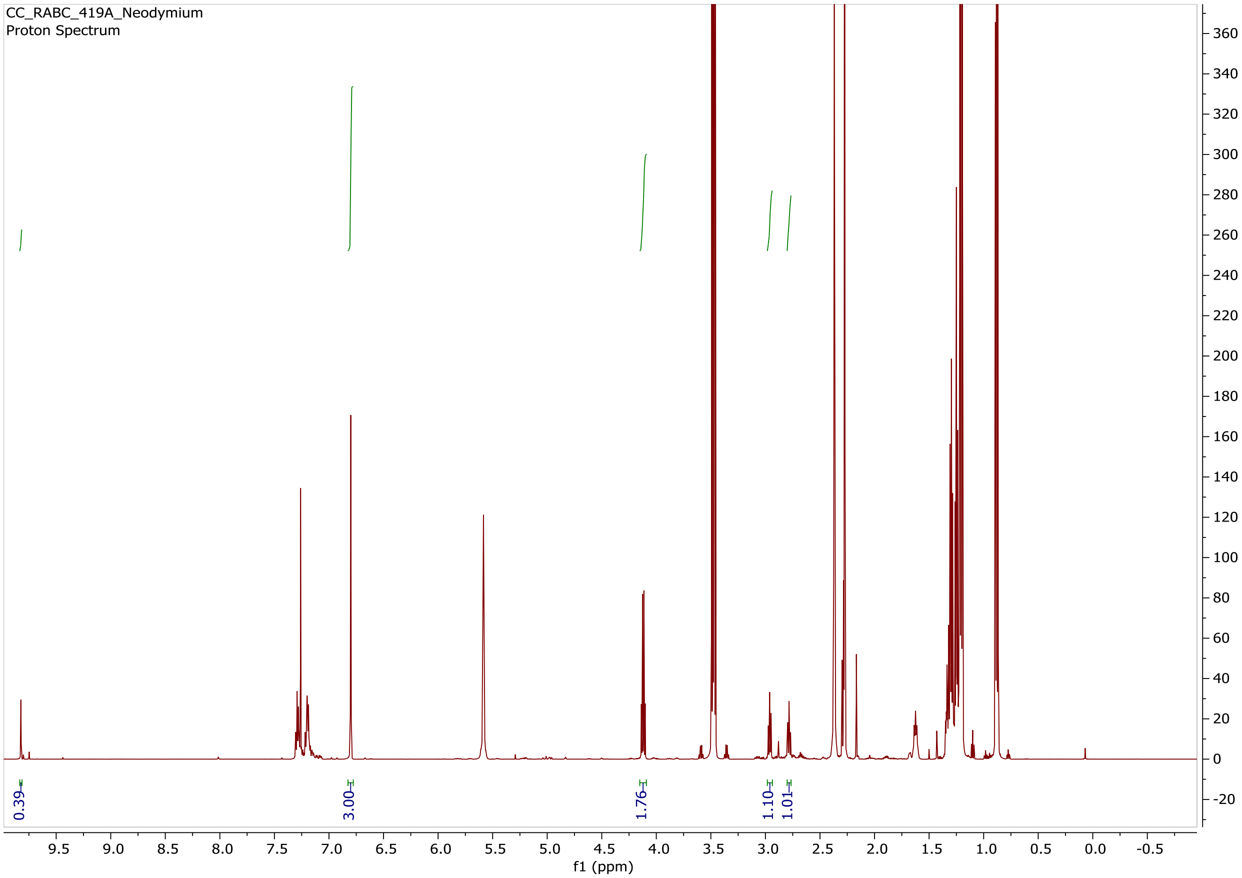

Stoichiometric Reaction – Ni-Catalyzed Alpha-Deoxygenation:

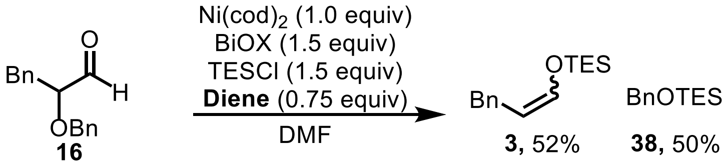

The above reaction was carried out by modifying the general procedure by replacing the dihydrocinnamaldehyde with aldehyde **16** and omitting the alkyl bromide. NMR integrations reveal 23% remaining aldehyde (9.68 ppm), 52% **3** (~6.33 ppm), and 50% **38** (4.73 ppm).

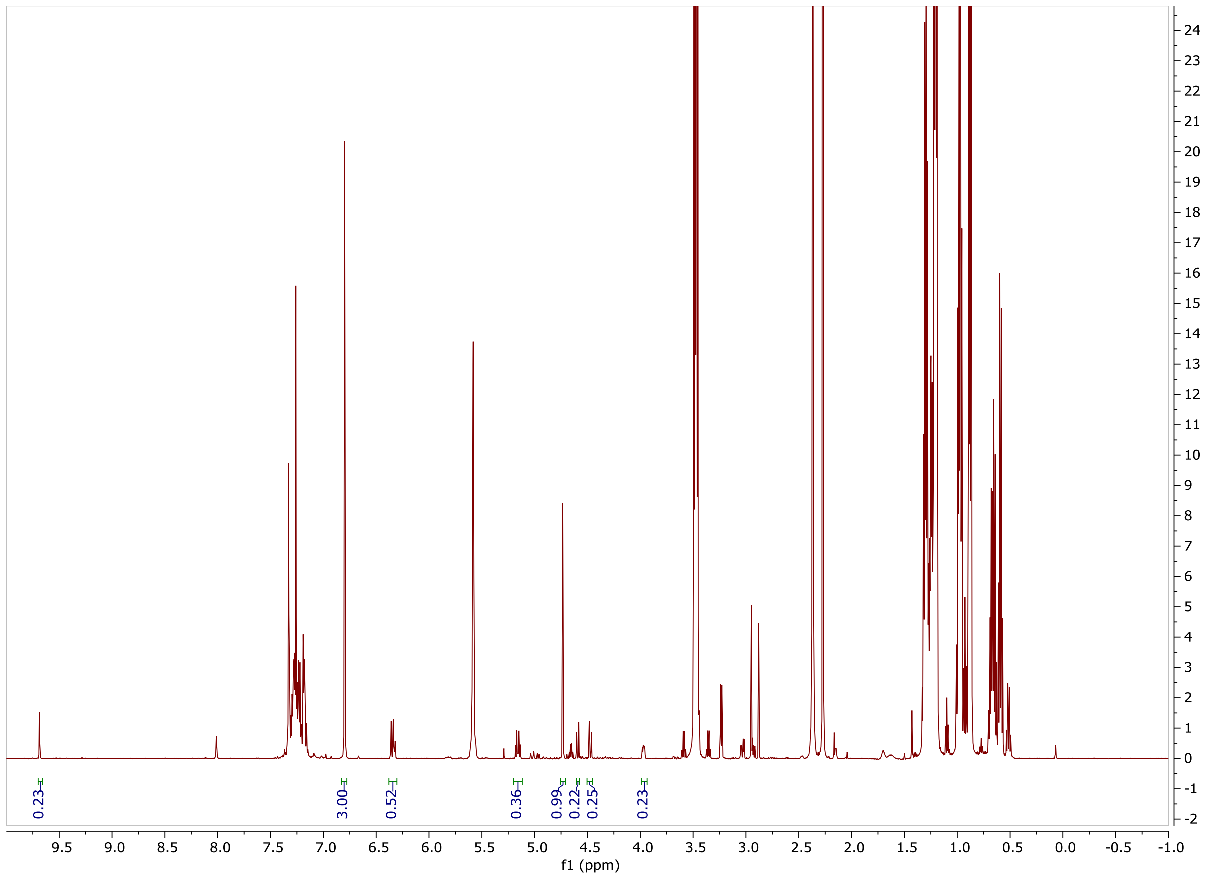
0.23
3.00
0.52
0.36
0.99
0.22
0.25
0.23
f1 (ppm)

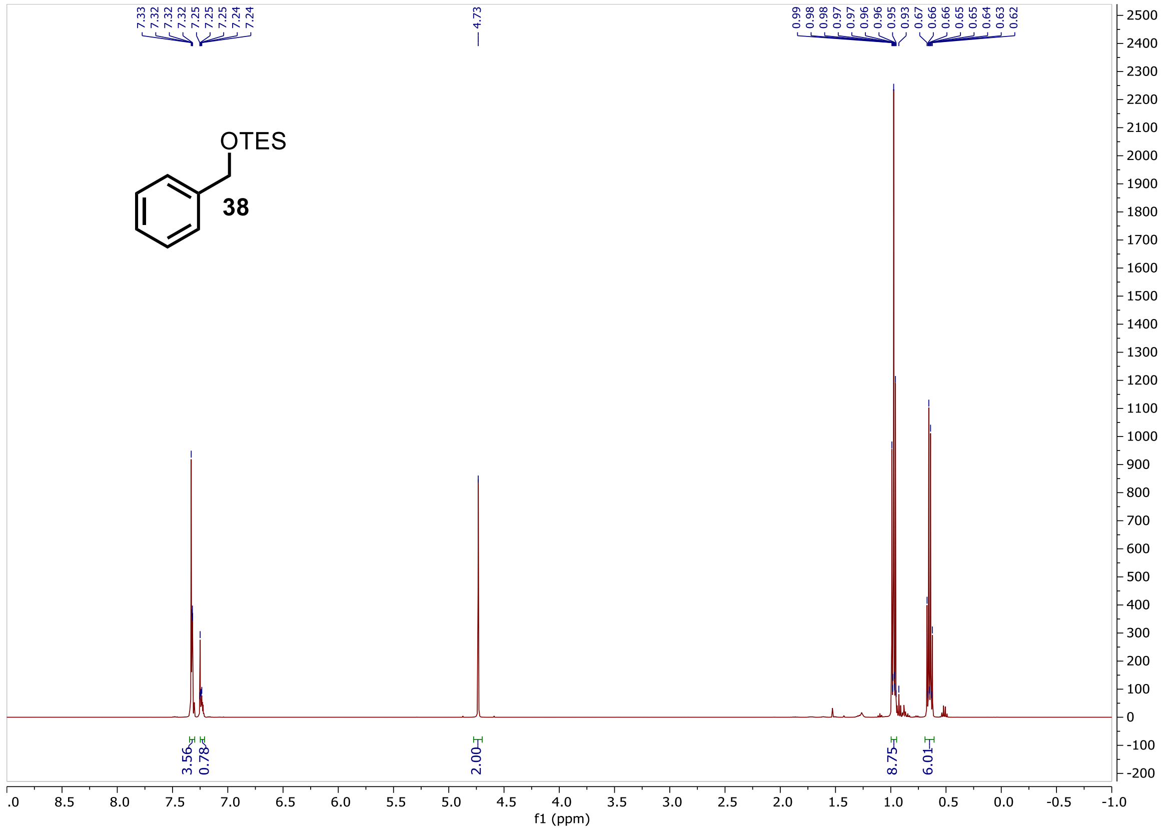
OTES
38
f1 (ppm)

V. Reductive Coupling Procedure and Characterization Data

General Procedure for Reductive Coupling: An oven-dried, 8 mL culture tube equipped with a stir bar was brought into a nitrogen-filled glovebox. The Ni precatalyst (0.10 equiv.), ligand (0.15 equiv.), manganese (4.0 equiv.) and sodium iodide (0.50 equiv.), which was then sealed with a PTFE-coated septum screw-cap. The tube with solid reagents was then removed from the glovebox and placed under a nitrogen atmosphere. The vial was then charged with DMF (to achieve a 0.5 M concentration with respect to aldehyde substrate) and 1,5-hexadiene (0.75 equiv.). The mixture was then stirred at 1100 rpm for 10 minutes after which point the aldehyde (1.0 equiv.), chlorotriethylsilane (1.5 equiv.) and bromide (1.0 – 2.0 equiv.) were added sequentially, neat. The nitrogen needle was then removed, the vial caps were sealed with electrical tape, and the mixture was allowed to stir at 1100 rpm at 20 °C for 5-16 hours. Reactions were then quenched with sat. aqueous NH_4Cl and diluted with ether. The layers were then separated and the aqueous layer was washed twice more with ether. The combined organics were then washed with brine, dried over sodium sulfate, filtered and concentrated. The resulting residue was then purified via silica gel column chromatography (using EtOAc / hexanes mixtures as mobile phase) to afford the products.

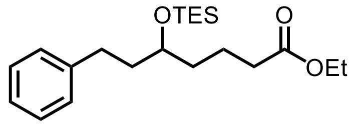

Ethyl 7-phenyl-5-((triethylsilyl)oxy)heptanoate (2): Synthesized by the general procedure using 0.5 mmol dihydrocinnamaldehyde and 1.0 mmol ethyl 4-bromobutyrate and a reaction time of 5 hours. The crude residue was purified via column chromatography to afford the product in 82% yield (average of two runs) as a light-yellow oil.

^{1}H NMR (500 MHz, $CDCl_3$) δ 7.30 – 7.26 (m, 2H), 7.21 – 7.13 (m, 3H), 4.13 (q, *J* = 7.1 Hz, 2H), 3.74 (m, 1H), 2.64 (m, 2H), 2.30 (t, *J* = 7.4 Hz, 2H), 1.88 – 1.43 (m, 6H), 1.26 (t, *J* = 7.1 Hz, 3H), 0.97 (t, *J* = 7.9 Hz, 9H), 0.61 (q, *J* = 7.9 Hz, 6H); **^{13}C NMR** (126 MHz, $CDCl_3$) δ 173.5, 142.5, 128.32, 128.30, 125.6, 71.5, 60.2, 36.4, 34.4, 31.6, 30.9, 20.9, 14.2, 6.9, 5.1; **HRMS (ESI+):** *m/z* calculated for [M+Na]: 387.2331 found: 387.2322

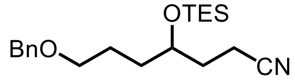

8-(Benzyloxy)-4-((triethylsilyl)oxy)octanenitrile (5): Synthesized by the general procedure using 0.5 mmol 4-(benzyloxy)butanal and 1.0 mmol 3-bromopropionitrile and a reaction time of 5 hours. The crude residue was purified via column chromatography to afford the product in 76% yield (average of two runs) as a light-yellow oil.

^{1}H NMR (500 MHz, $CDCl_3$) δ 7.37 – 7.32 (m, 4H), 7.29 (m, 1H), 4.50 (m, 2H), 3.82 (m, 1H), 3.47 (t, *J* = 6.1 Hz, 2H), 2.49 – 2.33 (m, 2H), 1.81 (s, 1H), 1.77 – 1.67 (m, 1H), 1.66 – 1.48 (m,

4H), 0.96 (t, *J* = 8.0 Hz, 9H), 0.61 (q, *J* = 7.9 Hz, 6H); **^{13}C NMR** (126 MHz, $CDCl_3$) δ 138.4, 128.3, 127.59, 127.54, 72.9, 70.1, 70.0, 33.5, 32.3, 25.3, 13.0, 6.8, 5.0; **HRMS (ESI+):** *m/z* calculated for [M+H]: 438.2359 found: 438.2346

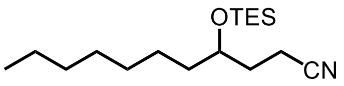

4-((Triethylsilyl)oxy)undecanenitrile (6): Synthesized by the general procedure using 0.5 mmol Octanal and 1.0 mmol 3-bromopropionitrile and a reaction time of 16 hours. The crude residue was purified via column chromatography to afford the product in 71% yield (average of two runs) as a light-yellow oil.

^{1}H NMR (500 MHz, $CDCl_3$) δ 3.77 (m, 1H), 2.40 (m, 2H), 1.81 (m, 1H), 1.76 – 1.61 (m, 1H), 1.52 – 1.38 (m, 2H), 1.27 (m, 10H), 0.96 (t, *J* = 7.9 Hz, 9H), 0.88 (t, *J* = 7.1 Hz, 3H), 0.61 (d, *J* = 7.9 Hz, 6H); **^{13}C NMR** (126 MHz, $CDCl_3$) δ 120.1, 70.3, 36.9, 32.2, 31.7, 29.6, 29.2, 25.0, 22.6, 14.0, 13.0, 6.8, 5.0; **HRMS (ESI+):** *m/z* calculated for [M+H]: 298.2566 found: 298.2560

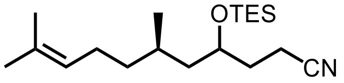

(6R)-6,10-dimethyl-4-((triethylsilyl)oxy)undec-9-enenitrile (7): Synthesized by the general procedure using 0.5 mmol (R)-Citronellal and 1.0 mmol 3-bromopropionitrile and a reaction time of 5 hours. The crude residue was purified via column chromatography to afford the product as a 1:1 mixture of diastereomers in 74% yield (average of two runs) as a light-yellow oil.

^{1}H NMR (600 MHz, $CDCl_3$) δ 5.13 – 5.05 (m, 1H), 3.86 (m, 1H), 2.47 – 2.33 (m, 2H), 2.04 – 1.90 (m, 1H), 1.85 (s, 0H), 1.76 – 1.70 (m, 1H), 1.69 (s, 2H), 1.64 (s, 0H), 1.60 (d, *J* = 1.2 Hz, 5H), 1.53 – 1.45 (m, 1H), 1.41 (s, 0H), 1.31 (s, 0H), 1.19 – 1.14 (m, 1H), 0.96 (td, *J* = 7.9, 0.9 Hz, 12H), 0.89 (d, *J* = 6.4 Hz, 4H), 0.65 – 0.59 (m, 8H); **^{13}C NMR** (151 MHz, $CDCl_3$) δ 131.4, 124.49, 124.44, 120.13, 120.10, 68.5, 68.4, 44.4, 37.6, 37.1, 33.0, 32.3, 29.1, 28.7, 25.72, 25.70, 25.33, 25.30, 19.8, 19.6, 17.6, 12.8, 12.7, 5.09, 5.06; **HRMS (ESI+):** *m/z* calculated for [M+H]: 324.2723 found: 324.2719

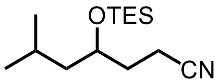

6-Methyl-4-((triethylsilyl)oxy)heptanenitrile (8): Synthesized by the general procedure using 0.5 mmol Isovaleraldehyde and 1.0 mmol 3-bromopropionitrile and a reaction time of 5 hours. The crude residue was purified via column chromatography to afford the product in 68% yield (average of two runs) as a light-yellow oil.

^{1}H NMR (500 MHz, $CDCl_3$) δ 3.85 (m, 1H), 2.47 – 2.35 (m, 2H), 1.92 – 1.80 (m, 1H), 1.75 – 1.56 (m, 2H), 1.38 (m, 1H), 1.33 – 1.26 (m, 1H), 0.97 (t, *J* = 7.9 Hz, 9H), 0.90 (m, 6H), 0.61 (q, *J* = 7.9 Hz, 6H); **^{13}C NMR** (126 MHz, $CDCl_3$) δ 120.1, 68.6, 46.1, 32.6, 24.6, 23.2, 22.5, 12.7, 6.8, 5.0; **HRMS (ESI+):** *m/z* calculated for [M+H]: 256.2097 found: 256.2099

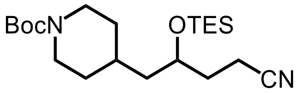

Tert-butyl 4-(4-cyano-2-((triethylsilyl)oxy)butyl)piperidine-1-carboxylate (9): Synthesized by the general procedure using 0.5 mmol tert-butyl 4-(2-oxoethyl)piperidine-1-carboxylate and 1.0 mmol 3-bromopropionitrile and a reaction time of 16 hours. The crude residue was purified via column chromatography to afford product in 76% yield (average of two runs) as a light-yellow oil.

^{1}H NMR (500 MHz, $CDCl_3$) δ 4.08 (br, 2H), 3.89 (m, 1H), 2.67 (br, 2H), 2.49 – 2.34 (m, 2H), 1.85 (m, 1H), 1.75 – 1.68 (m, 1H), 1.64 (m, 2H), 1.45 (m, 9H), 1.38 – 1.29 (m, 1H), 1.14 – 1.04 (m, 2H), 0.96 (t, *J* = 7.9 Hz, 9H), 0.61 (q, *J* = 7.9 Hz, 6H); **^{13}C NMR** (126 MHz, $CDCl_3$) δ 154.7, 119.8, 79.3, 67.6, 43.7, 32.6 (major rotamer), 32.5 (minor rotamer), 32.4 (major rotamer), 32.3 (minor rotamer), 28.4, 12.7, 6.8, 5.0; **HRMS (ESI+):** *m/z* calculated for [M+Na]: 419.2706 found: 419.2696

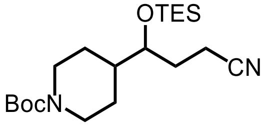

Tert-butyl 4-(3-cyano-1-((triethylsilyl)oxy)propyl)piperidine-1-carboxylate (10): Synthesized by the general procedure using 0.5 mmol tert-butyl 4-formylpiperidine-1-carboxylate and 1.0 mmol 3-bromopropionitrile and a reaction time of 16 hours. The crude residue was purified via column chromatography to afford the product in 76% yield (average of two runs) as a light-yellow oil.

^{1}H NMR (500 MHz, $CDCl_3$) δ 4.16 (br, 2H), 3.61 (m, 1H), 2.62 (br, 2H), 2.40 (m, 2H), 1.76 (m, 2H), 1.61 (m, 2H), 1.56 – 1.50 (m, 1H), 1.45 (s, 9H), 1.23 – 1.10 (m, 2H), 0.96 (t, *J* = 7.9 Hz, 9H), 0.62 (q, *J* = 7.9 Hz, 6H); **^{13}C NMR** (126 MHz, $CDCl_3$) δ 154.7, 119.8, 79.4, 73.7, 41.7, 28.9, 28.4, 28.0, 27.1, 12.9, 6.9, 5.1; **HRMS (ESI+):** *m/z* calculated for [M+Na]: 405.2549 found: 405.2540

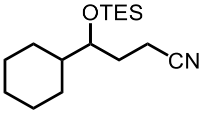

4-Cyclohexyl-4-((triethylsilyl)oxy)butanenitrile (11): Synthesized by the general procedure using 0.5 mmol Cyclohexanecarboxaldehyde, 1.0 mmol 3-bromopropionitrile, omitting NaI and a reaction time of 16 hours. The crude residue was purified via column chromatography to afford the product in 57% yield (average of two runs) as a light-yellow oil.

^{1}H NMR (500 MHz, $CDCl_3$) δ 3.56 (m, 1H), 2.46 – 2.34 (m, 2H), 1.79 – 1.65 (m, 8H), 1.39 (m, 1H), 1.25 – 1.06 (m, 3H), 0.97 (t, *J* = 7.9 Hz, 9H), 0.62 (q, *J* = 7.8 Hz, 6H); **^{13}C NMR** (126 MHz, $CDCl_3$) δ 120.1, 74.6, 43.4, 29.0, 28.9, 27.6, 26.5, 26.4, 26.2, 13.2, 6.9, 5.1; **HRMS (ESI+):** *m/z* calculated for [M+H]: 282.2253 found: 282.2243

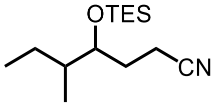

5-Methyl-4-((triethylsilyl)oxy)heptanenitrile (12): Synthesized by the general procedure using 0.5 mmol 2-Methylbutyraldehyde, 1.0 mmol 3-bromopropionitrile, omitting NaI and a reaction time of 16 hours. The crude residue was purified via column chromatography to afford the product as a 1:1 mixture of diastereomers in 45% yield (average of two runs) as a light-yellow oil.

^{1}H NMR (500 MHz, $CDCl_3$) δ 3.73 – 3.60 (m, 1H), 2.52 – 2.29 (m, 2H), 1.84 – 1.63 (m, 2H), 1.54 (s, 1H), 1.47 – 1.29 (m, 1H), 1.12 (m, 1H), 0.97 (t, J = 7.9 Hz, 9H), 0.93 – 0.88 (m, 3H), 0.85 (d, J = 6.9 Hz, 3H), 0.63 (q, J = 7.9, 6H); **^{13}C NMR** (151 MHz, $CDCl_3$) δ 120.1, 120.0, 74.6, 73.7, 40.5, 40.3, 28.3, 27.5, 25.9, 23.6, 12.14, 12.10, 6.91, 6.90 5.12, 5.10; **HRMS (CI+):** *m/z* calculated for [M+H]: 256.2097 found: 256.2093

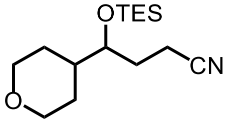

4-(Tetrahydro-2H-pyran-4-yl)-4-((triethylsilyl)oxy)butanenitrile (13): Synthesized by the general procedure using 0.5 mmol tetrahydro-2H-pyran-4-carbaldehyde, 1.0 mmol 3-bromopropionitrile, omitting NaI and a reaction time of 16 hours. The crude residue was purified via column chromatography to afford the product in 56% yield (average of two runs) as a light-yellow oil.

^{1}H NMR (500 MHz, $CDCl_3$) δ 4.01 (m, 2H), 3.60 (m, 1H), 3.35 (m, 2H), 2.41 (m, 2H), 1.83 – 1.75 (m, 2H), 1.68 – 1.56 (m, 2H), 1.55 – 1.50 (m, 1H), 1.36 (m, 2H), 0.97 (t, J = 8.0 Hz, 9H), 0.63 (q, J = 7.9 Hz, 6H); **^{13}C NMR** (126 MHz, $CDCl_3$) δ 119.8, 73.8, 67.8, 40.5, 29.0, 28.9, 28.1, 12.8, 6.9, 5.1; **HRMS (CI+):** *m/z* calculated for [M+H]: 284.2046 found: 284.2047

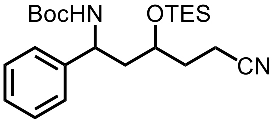

Tert-butyl (5-cyano-1-phenyl-3-((triethylsilyl)oxy)pentyl)carbamate (14): Synthesized by the general procedure using 0.5 mmol tert-butyl (3-oxo-1-phenylpropyl)carbamate and 1.0 mmol 3-bromopropionitrile and a reaction time of 16 hours. The crude residue was purified via column chromatography to afford the product as a 1:1 mixture of diastereomers in 48% yield (average of two runs) as a light-yellow oil.

^{1}H NMR (600 MHz, CD_3CN) δ 7.39 – 7.26 (m, 5H), 5.20 – 5.10 (br, 0.5H, diastereomer A), 4.70 – 4.56 (br, 0.5H, diastereomer B), 3.95 – 3.85 (br, 0.5 H, diastereomer A), 3.83 – 3.74 (br, 0.5H, diastereomer B), 2.46 – 2.35 (m, 3H), 1.90 – 1.80 (m, 4H), 1.40 (s, 9H), 1.04 – 0.83 (m, 9H), 0.70 – 0.51 (m, 6H); **^{13}C NMR** (151 MHz, CD_3CN) δ 156.2, 129.4, 128.0, 127.8, 127.3, 127.1, 126.9, 126.8, 121.3, 79.3, 69.6, 68.7, 52.7, 44.6, 44.2, 33.8, 33.0, 28.6, 13.3, 13.2, 7.3, 7.2, 5.7, 5.7; **HRMS (ESI+):** *m/z* calculated for [M+Na]: 441.2549 found: 441.2538

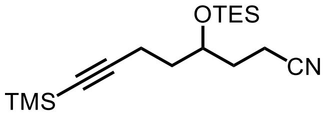

4-((Triethylsilyl)oxy)-8-(trimethylsilyl)oct-7-ynenitrile (15): Synthesized by the general procedure using 0.5 mmol 5-(trimethylsilyl)pent-4-ynal and 1.0 mmol 3-bromopropionitrile and a reaction time of 16 hours. The crude residue was purified via column chromatography to afford the product in 30% yield (average of two runs) as a light-yellow oil.

^{1}H NMR (500 MHz, $CDCl_3$) δ 4.00 – 3.92 (m, 1H), 2.45 – 2.37 (m, 2H), 2.28 (m, 2H), 1.87 (m, 1H), 1.82 – 1.57 (m, 3H), 0.97 (t, *J* = 7.9 Hz, 9H), 0.64 (q, *J* = 8.0 Hz, 6H), 0.15 (s, 9H); **^{13}C NMR** (126 MHz, $CDCl_3$) δ 119.84, 106.14, 85.45, 68.92, 35.15, 32.22, 15.84, 12.81, 6.88, 5.00, 0.04; **HRMS (ESI+):** *m/z* calculated for [M+H]: 324.2179 found: 324.2171

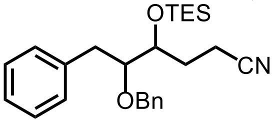

5-(Benzyloxy)-6-phenyl-4-((triethylsilyl)oxy)hexanenitrile (16): Synthesized by the general procedure using 0.5 mmol 2-(benzyloxy)-3-phenylpropanal, 1.0 mmol 3-bromopropionitrile, omitting NaI and a reaction time of 16 hours. The crude residue was purified via column chromatography to afford the product in 30% yield (average of two runs) as a light-yellow oil.

^{1}H NMR (500 MHz, $CDCl_3$) δ 7.34 – 7.19 (m, 8H), 7.19 – 7.07 (m, 2H), 4.52 (d, *J* = 11.4 Hz, 0.67H, major diastereomer), 4.33 (d, *J* = 11.5 Hz, 0.67H, major diastereomer), 4.28 (m, 0.67H, minor diastereomer), 3.97 – 3.91 (m, 0.33H, minor diastereomer), 3.84 (m, 0.67H, major diastereomer), 3.64 – 3.59 (m, 0.67H, major diastereomer), 3.59 – 3.55 (m, 0.33H, minor diastereomer), 2.98 (d, *J* = 13.9, 0.33H, minor diastereomer), 2.81 (m, 1.34H, major diastereomer), 2.59 (dd, *J* = 13.8, 10.3 Hz, 0.33H, minor diastereomer), 2.55 – 2.33 (m, 2H, major + minor diastereomer), 2.15 – 2.07 (m, 0.33H, minor diastereomer), 2.06 – 1.97 (m, 0.66H, major diastereomer), 1.90 – 1.82 (m, 0.66H, major diastereomer), 1.80 – 1.71 (m, 0.33H, minor diastereomer), 1.00 – 0.87 (m, 9H, major + minor diastereomer), 0.59 (m, 6H, major + minor diastereomer); **^{13}C NMR** (126 MHz, $CDCl_3$) δ 138.7, 138.2, 138.0, 129.3, 128.46, 128.2, 127.9, 127.7, 127.5, 126.3, 120.0, 84.0, 73.2, 72.8, 72.4, 38.2, 35.0, 28.1, 27.2, 14.1, 13.2, 6.8, 5.7, 4.9; **HRMS (ESI+):** *m/z* calculated for [M+H]: 410.2515 found: 410.2505

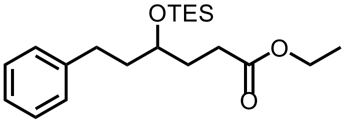

Ethyl 6-phenyl-4-((triethylsilyl)oxy)hexanoate (17): Synthesized by the general procedure using 0.5 mmol dihydrocinnamaldehyde, 1.0 mmol Ethyl 3-bromopropiolate, omitting NaI and a reaction time of 16 hours. The crude residue was purified via column chromatography to afford the product in 84% yield (average of two runs) as a light-yellow oil.

^{1}H NMR (500 MHz, $CDCl_3$) δ 7.30 – 7.26 (m, 3H), 7.18 (m, 2H), 4.13 (q, J = 7.1 Hz, 2H), 3.83 – 3.74 (m, 1H), 2..71 – 2.59 (m, 2H), 2.44 – 2.33 (m, 2H), 1.92 – 1.84 (m, 1H), 1.83 – 1.73 (m, 3H), 1.26 (t, J = 7.1 Hz, 3H), 0.97 (t, J = 8.0 Hz, 49), 0.61 (q, J = 7.9 Hz, 6H);**^{13}C NMR (**126 MHz, $CDCl_3$) δ 173.8, 142.3, 128.3, 128.2, 125.7, 70.8, 60.2, 38.9, 31.7, 31.5, 30.0, 14.2, 6.9, 5.0; **HRMS (ESI+):** *m/z* calculated for [M+Na+]: 373.2175 found: 373.2168

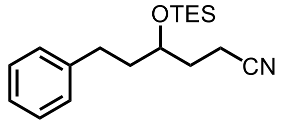

6-Phenyl-4-((triethylsilyl)oxy)hexanenitrile (18): Synthesized by the general procedure using 0.5 mmol dihydrocinnamaldehyde, 1.0 mmol 3-bromopropionitrile and a reaction time of 5 hours. The crude residue was purified via column chromatography to afford the product in 82% yield (average of two runs) as a light yellow oil. For the 5.0 mmol scale reaction, the general procedure was modified in the following way: the reaction was performed in a flame-dried 25 mL round-bottom flask and setup on the benchtop. Workup and purification in a similar fashion afforded the product in 69% yield as a yellow oil.

^{1}H NMR (500 MHz, $CDCl_3$) δ 7.29 (m, 2H), 7.23 – 7.15 (m, 3H), 3.86 (m, 1H), 2.64 (t, J = 8.3 Hz, 2H), 2.46 – 2.39 (m, 2H), 1.93 – 1.74 (m, 4H), 0.97 (t, J = 8.0 Hz, 9H), 0.63 (q, J = 8.0 Hz, 6H);**^{13}C NMR** (126 MHz, $CDCl_3$) δ 141.6, 128.4, 128.2, 125.9, 119.9, 69.9, 38.7, 32.3, 31.4, 13.0, 6.8, 5.0; **HRMS (ESI+):** *m/z* calculated for [M+H]: 304.2097 found: 304.2095

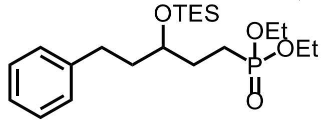

Diethyl (5-phenyl-3-((triethylsilyl)oxy)pentyl)phosphonate (19): Synthesized by the general procedure using 0.5 mmol dihydrocinnamaldehyde, 1.0 mmol Diethyl 2-bromoethylphosphonate, omitting NaI and a reaction time of 16 hours. The crude residue was purified via column chromatography to afford the product in 75% yield as a light-yellow oil.

^{1}H NMR (500 MHz, $CDCl_3$) δ 7.30 – 7.26 (m, 2H), 7.21 – 7.14 (m, 3H), 4.15 – 4.03 (m, 4H), 3.81 – 3.73 (m, 1H), 2.70 – 2.56 (m, 2H), 1.88 – 1.70 (m, 6H), 1.32 (t, J = 7.1 Hz, 6H), 0.96 (t, J = 8.0 Hz, 9H), 0.60 (q, J = 8.0 Hz, 6H);**^{13}C NMR** (126 MHz, $CDCl_3$) δ 141.6, 128.4, 128.2, 125.9, 119.9, 69.9, 38.7, 32.3, 31.4, 13.0, 6.8, 5.0; **^{31}P{^{1}H} NMR** (202 MHz, $CDCl_3$) δ 32.94; **HRMS (ESI+):** *m/z* calculated for [2M+Na]: 851.4608 found: 851.4627

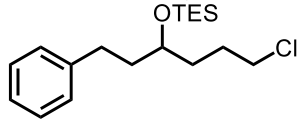

((6-Chloro-1-phenylhexan-3-yl)oxy)triethylsilane (20): Synthesized by the general procedure using 0.5 mmol dihydrocinnamaldehyde, 1.0 mmol 1-bromo-3-chloropropane and a reaction time of 16 hours. The crude residue was purified via column chromatography to afford the product in 74% yield as a light-yellow oil.

^{1}H NMR (500 MHz, $CDCl_3$) δ 7.28 (m, 2H), 7.18 (m, 3H), 3.82 – 3.73 (m, 1H), 3.55 (t, J = 6.6 Hz, 2H), 2.73 – 2.56 (m, 2H), 1.95 – 1.74 (m, 4H), 1.73 – 1.55 (m, 2H), 0.97 (t, J = 7.9 Hz, 9H),

0.61 (q, J = 7.9 Hz, 6H); **^{13}C NMR** (126 MHz, $CDCl_3$) δ 142.3, 128.3, 128.2, 125.7, 71.2, 45.4, 39.0, 34.2, 31.7, 28.5, 6.9, 5.1; **HRMS (CI):** *m/z* calculated for [M+H]: 351.2355 found: 351.2344

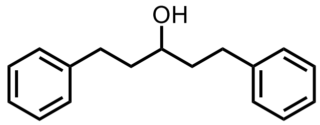

1,5-Diphenylpentan-3-ol (21): Synthesized by the general procedure using 0.5 mmol dihydrocinnamaldehyde, 1.0 mmol 2-bromoethylbenzene and a reaction time of 16 hours. After workup, the residue was dissolved in 2 mL THF and treated w/ 2.0 mL 1M TBAF (THF solution) and stirred for 2 hours. The solution was then concentrated and the residue was purified via column chromatography to afford the alcohol as a clear oil in 71% yield (average of 2 trials). Spectral data were in agreement with previously reported preparations.[21]

^{1}H NMR (401 MHz, $CDCl_3$) δ 7.31 – 7.24 (m, 5H), 7.22 – 7.16 (m, 5H), 3.71 – 3.62 (m, 1H), 2.84 – 2.74 (m, 2H), 2.72 – 2.62 (m, 2H), 1.90 – 1.71 (m, 4H)

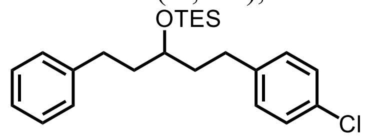

((1-(4-Chlorophenyl)-5-phenylpentan-3-yl)oxy)triethylsilane (22): Synthesized by the general procedure using 0.5 mmol dihydrocinnamaldehyde and 1.0 mmol 1-chloro-4-(2-chloroethyl)benzene and a reaction time of 16 hours. The crude residue was purified via column chromatography to afford the product in 63% yield as a light-yellow oil.

^{1}H NMR (600 MHz, $CDCl_3$) δ 7.30 – 7.27 (m, 2H), 7.25 (d, J = 8.4 Hz, 2H), 7.21 – 7.17 (m, 3H), 7.10 (d, J = 8.4 Hz, 2H), 3.80 – 3.75 (m, J = 5.7 Hz, 1H), 2.75 – 2.53 (m, 4H), 1.87 – 1.78 (m, 4H), 0.99 (t, J = 7.9 Hz, 9H), 0.62 (q, J = 7.9 Hz, 6H); **^{13}C NMR** (151 MHz, $CDCl_3$) δ 142.4, 140.9, 131.3, 129.6, 128.4, 128.37, 128.30, 125.7, 71.3, 39.0, 38.8, 31.7, 31.0, 6.9, 5.1; **HRMS (CI):** *m/z* calculated for [M+H]: 389.2067 found: 389.2050

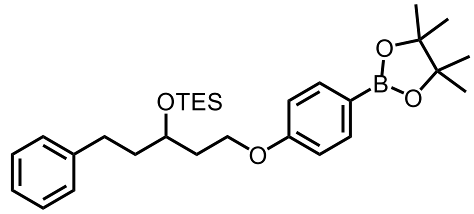

Triethyl((1-phenyl-5-(4-(4,4,5,5-tetramethyl-1,3,2-dioxaborolan-2-yl)phenoxy)pentan-3-yl)oxy)silane (23): Synthesized by the general procedure using 0.2 mmol dihydrocinnamaldehyde and 0.2 mmol **S4** and a reaction time of 16 hours. The crude residue was purified via column chromatography to afford the product in 43% yield (average of 2 trials) as a light-yellow oil.

^{1}H NMR (500 MHz, $CDCl_3$) δ 7.77 – 7.71 (m, 2H), 7.32 – 7.25 (m, 2H), 7.23 – 7.16 (m, 3H), 6.91 – 6.85 (m, 2H), 4.13 – 4.06 (m, 2H), 4.05 – 3.99 (m, 1H), 2.73 – 2.64 (m, 2H), 2.07 – 1.99

(m, 1H), 1.97 – 1.88 (m, *J* = 7.6 Hz, 1H), 1.87 – 1.80 (m, 2H), 1.34 (s, 12H), 0.94 (t, *J* = 7.9 Hz, 9H), 0.58 (q, *J* = 7.9 Hz, 6H); **^{13}C NMR** (126 MHz, $CDCl_3$) δ 161.5, 142.3, 136.4, 128.37, 128.32, 125.7, 113.7, 83.5, 68.7, 64.2, 39.5, 36.2, 31.5, 24.8, 6.9, 5.0; **HRMS (ESI+):** *m/z* calculated for [M+H]: 519.3078 found: 519.3070

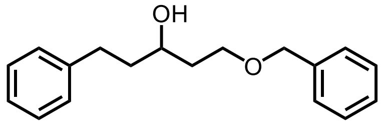

1-(Benzyloxy)-5-phenylpentan-3-ol (24): Synthesized by the general procedure using 0.5 mmol dihydrocinnamaldehyde, 1.0 mmol ((2-bromoethoxy)methyl)benzene, omitting NaI and a reaction time of 16 hours. After workup, the residue was dissolved in 2 mL THF and treated w/ 2.0 mL 1M TBAF (THF solution) and stirred for 2 hours. The solution was then concentrated and the residue was purified via column chromatography to afford the alcohol as a clear oil in 43% yield (average of 2 trials). Spectral data were in agreement with previously reported preparations.[22]

^{1}H NMR ^{1}H NMR (600 MHz, $CDCl_3$) δ 7.38 – 7.27 (m, 7H), 7.22 – 7.15 (m, 3H), 4.52 (s, 2H), 3.85 (m, 1H), 3.75 – 3.70 (m, 1H), 3.68 – 3.62 (m, 1H), 2.94 (s, 1H), 2.83 – 2.76 (m, 1H), 2.72 – 2.63 (m, 1H), 1.78 (m, 4H); **^{13}C NMR** (150 MHz, $CDCl_3$) δ 142.2, 137.8, 128.4, 128.4, 128.3, 127.7, 127.6, 125.7, 73.3, 70.8, 69.2, 39.1, 36.4, 31.9; **HRMS (ESI+):** *m/z* calculated for [M+H]: 519.3078 found: 519.3070

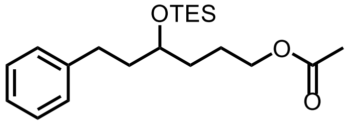

6-Phenyl-4-((triethylsilyl)oxy)hexyl acetate (25): Synthesized by the general procedure using 0.5 mmol dihydrocinnamaldehyde and 1.0 mmol 3-bromopropyl acetate and a reaction time of 5 hours. The crude residue was purified via column chromatography to afford the product in 61% yield (average of 2 trials) as a light-yellow oil.

^{1}H NMR (500 MHz, $CDCl_3$) δ 7.30 – 7.25 (m, 2H), 7.20 – 7.15 (m, 3H), 4.11 – 4.03 (m, 1H), 3.79 – 3.72 (m, 1H), 2.74 – 2.56 (m, 2H), 2.05 (s, 3H), 1.82 – 1.48 (m, 6H), 0.97 (t, *J* = 7.9 Hz, 9H), 0.61 (q, *J* = 7.9 Hz, 6H); **^{13}C NMR** (126 MHz, $CDCl_3$) δ 171.1, 142.4, 128.3, 128.2, 125.7, 71.3, 64.6, 38.9, 33.2, 31.7, 24.4, 20.9, 6.9, 5.1; **HRMS (CI):** *m/z* calculated for [M+H]: 351.2355 found: 351.2344

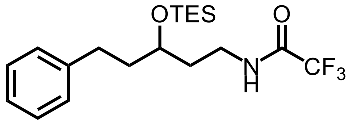

2,2,2-Trifluoro-N-(5-phenyl-3-((triethylsilyl)oxy)pentyl)acetamide (26): Synthesized by the general procedure using 0.5 mmol dihydrocinnamaldehyde and 1.0 mmol N-(2-bromoethyl)-2,2,2-trifluoroacetamide and a reaction time of 16 hours. The crude residue was purified via column chromatography to afford the product in 56% yield as a light-yellow oil.

^{1}H NMR (500 MHz, $CDCl_3$) δ 7.46 (br, 1H), 7.32 – 7.27 (m, 2H), 7.24 – 7.18 (m, 1H), 7.18 – 7.13 (m, 2H), 3.98 – 3.92 (m, 1H), 3.54 – 3.48 (m, 2H), 2.66 – 2.55 (m, 2H), 1.95 – 1.81 (m, 3H), 1.81 – 1.73 (m, 1H), 0.96 (t, *J* = 8.0 Hz, 9H), 0.66 – 0.56 (q, *J* = 8.0 Hz, 6H); **^{13}C NMR**

(126 MHz, $CDCl_3$) δ 156.9 (q, $^{2}J_{CF}$ = 36.7 Hz), 141.3, 128.5, 128.1, 126.0, 115.9 (q, $^{1}J_{CF}$ = 287.8 Hz), 71.5, 37.7, 36.8, 33.1, 31.8, 6.7, 4.7; **HRMS (ESI+):** *m/z* calculated for [M+H]: 390.2076 found: 390.2068

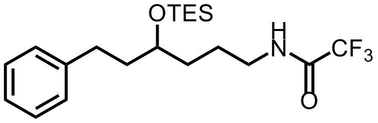

2,2,2-Trifluoro-N-(6-phenyl-4-((triethylsilyl)oxy)hexyl)acetamide (27): Synthesized by the general procedure using 0.5 mmol dihydrocinnamaldehyde and 1.0 mmol N-(3-bromopropyl)-2,2,2-trifluoroacetamide and a reaction time of 16 hours. The crude residue was purified via column chromatography to afford the product in 57% yield as a light-yellow oil.

^{1}H NMR (500 MHz, $CDCl_3$) δ 7.30 – 7.26 (m, 2H), 7.21 – 7.14 (m, 3H), 6.77 (br, 1H), 3.82 – 3.77 (m, 1H), 3.55 – 3.44 (m, 1H), 3.31 – 3.23 (m, 1H), 2.69 – 2.55 (m, 2H), 1.85 – 1.54 (m, 6H), 0.97 (t, *J* = 7.9 Hz, 9H), 0.61 (q, *J* = 7.8 Hz, 6H); **^{13}C NMR** (126 MHz, $CDCl_3$) δ 156.9 (q, $^{2}J_{CF}$ = 36.7 Hz), 141.3, 128.5, 128.1, 126.0, 115.9 (q, $^{1}J_{CF}$ = 287.8 Hz), 71.5, 37.7, 36.8, 33.1, 31.8, 6.7, 4.7; **HRMS (ESI+):** *m/z* calculated for [M+H]: 404.2233 found: 404.2219

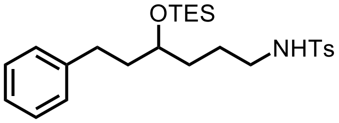

4-Methyl-N-(6-phenyl-4-((triethylsilyl)oxy)hexyl)benzenesulfonamide (28): Synthesized by the general procedure using 0.5 mmol dihydrocinnamaldehyde and 1.0 mmol N-(3-bromopropyl)-4-methylbenzenesulfonamide and a reaction time of 16 hours. The crude residue was purified via column chromatography to afford the product in 51% yield as a light-yellow oil.

^{1}H NMR (600 MHz, $CDCl_3$) δ 7.74 (d, *J* = 8.3 Hz, 1H), 7.31 – 7.24 (m, 4H), 7.21 – 7.16 (m, 1H), 7.16 – 7.12 (d, *J* = 8.3 Hz, 2H), 4.50 (t, *J* = 6.2 Hz, 1H), 3.75 – 3.61 (m, 1H), 2.99 – 2.90 (m, 2H), 2.63 – 2.53 (m, 2H), 2.41 (s, 3H), 1.75 – 1.65 (m, 2H), 1.57 – 1.40 (m, 4H), 0.93 (t, *J* = 7.9 Hz, 9H), 0.56 (q, *J* = 8.0 Hz, 6H); **^{13}C NMR** (126 MHz, $CDCl_3$) δ 143.2, 142.2, 137.0, 129.6, 128.3, 127.0, 125.6, 71.2, 38.7, 33.7, 31.5, 25.2, 21.4, 6.8, 4.9; **HRMS (ESI+):** *m/z* calculated for [M+Na]: 484.2318 found: 484.2334

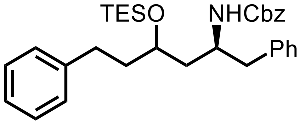

Benzyl ((2S)-1,6-diphenyl-4-((triethylsilyl)oxy)hexan-2-yl)carbamate (29): Synthesized by the general procedure using 0.5 mmol dihydrocinnamaldehyde, 1.0 mmol benzyl (S)-(1-bromo-3-phenylpropan-2-yl)carbamate, omitting NaI and a reaction time of 16 hours. The crude residue was purified via column chromatography to afford the product as a 1:1 mixture of diastereomers in 42% yield as a light-yellow oil.

^{1}H NMR (500 MHz, $CDCl_3$) δ 7.39 – 7.07 (m, 15H), 5.21 – 4.75 (m, 3H), 4.05 – 3.76 (m, 2H), 3.15 – 2.43 (m, 4H), 1.87 – 1.46 (m, 4H), 0.93 (t, *J* = 7.9 Hz, 9H), 0.57 (m, 6H); **^{13}C NMR** (126 MHz, $CDCl_3$) δ 155.7, 142.2, 141.8, 129.5, 128.45, 128.40, 128.36, 128.30, 128.2, 128.0, 127.9, 126.4, 126.3, 125.7, 125.6, 69.9, 69.5, 66.4, 66.2, 50.4, 50.0, 41.6, 41.2, 41.1, 39.3, 39.0, 38.6, 31.4, 31.1, 25.0, 6.9, 5.0; **HRMS (ESI+):** *m/z* calculated for [M+Na]: 540.2910 found: 540.2958

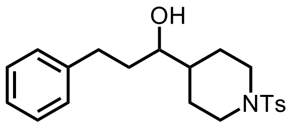

3-Phenyl-1-(1-tosylpiperidin-4-yl)propan-1-ol (30): Synthesized by the general procedure using 0.5 mmol dihydrocinnamaldehyde, 1.0 mmol 4-bromo-1-tosylpiperidine and replacing NaI w/ 1.0 equiv. LiBr and a reaction time of 16 hours. After workup, the residue was dissolved in 2 mL THF and treated w/ 2.0 mL 1M TBAF (THF solution) and stirred for 2 hours. The solution was then concentrated and the residue was purified via column chromatography to afford the alcohol as a white solid in 49% yield (average of 2 trials).

^{1}H NMR (500 MHz, $CDCl_3$) δ 7.63 (d, *J* = 8.0 Hz, 2H), 7.31 (d, *J* = 8.0 Hz, 1H), 7.29 – 7.26 (m, 2H), 7.22 – 7.14 (m, 3H), 3.87 – 3.79 (m, 2H), 3.43 – 3.35 (m, 1H), 2.83 – 2.76 (m, 1H), 2.66 – 2.58 (m, 1H), 2.43 (s, 3H), 2.24 – 2.14 (m, 2H), 1.88 – 1.81 (m, 1H), 1.81 – 1.73 (m, 1H), 1.72 – 1.61 (m, 2H), 1.51 – 1.34 (m, 3H), 1.33 – 1.19 (m, 1H); **^{13}C NMR** (126 MHz, $CDCl_3$) δ 143.3, 141.7, 133.2, 129.5, 128.4, 128.3, 127.7, 125.9, 74.2, 46.3, 46.2, 41.3, 35.8, 32.0, 27.6, 26.7, 21.5; **HRMS (ESI+):** *m/z* calculated for [M+Na]: 396.1609 found: 396.1606

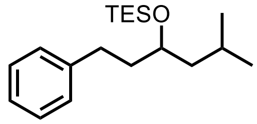

Triethyl((5-methyl-1-phenylhexan-3-yl)oxy)silane (31): Synthesized by the general procedure using 0.5 mmol dihydrocinnamaldehyde, 1.0 mmol isobutyl bromide and replacing NaI w/ 1.0 equiv. LiBr and a reaction time of 16 hours. The crude residue was purified via column chromatography to afford the product as a 1:1 mixture of diastereomers in 45% yield as a light-yellow oil.

^{1}H NMR (600 MHz, $CDCl_3$) δ 7.30 – 7.26 (m, 2H), 7.21 – 7.16 (m, 3H), 3.81 – 3.76 (m, 1H), 2.72 – 2.67 (m, 1H), 2.66 – 2.59 (m, 1H), 1.83 – 1.67 (m, 2H), 1.45 – 1.32 (m, 2H), 0.99 – 0.96 (m, 9H), 0.91 – 0.88 (m, 6H), 0.64 – 0.59 (m, 6H), **^{13}C NMR** (151 MHz, $CDCl_3$) δ 142.7, 128.3, 128.3, 125.6, 70.1, 46.5, 39.4, 31.5, 24.5, 23.0, 22.8, 6.9, 5.2; **HRMS (ESI+):** *m/z* calculated for [M+H]: 307.2457 found: 307.2445

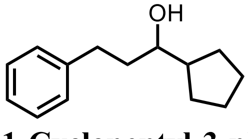

1-Cyclopentyl-3-phenylpropan-1-ol (32): Synthesized by the general procedure using 0.5 mmol dihydrocinnamaldehyde, 1.0 mmol cyclopentyl bromide and replacing NaI w/ 1.0 equiv. LiBr and a reaction time of 16 hours. After workup, the residue was dissolved in 2 mL THF and treated w/ 2.0 mL 1M TBAF (THF solution) and stirred for 2 hours. The solution was then concentrated and the residue was purified via column chromatography to afford the alcohol as a light-yellow oil in 52% yield (average of 2 trials). Spectral data were in agreement with previously reported preparations.[23]

^{1}H NMR (500 MHz, $CDCl_3$) δ 7.31 – 7.26 (m, 2H), 7.24 – 7.16 (m, 3H), 3.46 – 3.40 (m, 1H), 2.90 – 2.82 (m, 1H), 2.72 – 2.64 (m, 1H), 1.95 – 1.49 (m, 10H), 1.40 – 1.29 (m, 1H), 1.23 – 1.15 (m, 1H); **^{13}C NMR** (126 MHz, $CDCl_3$) δ 142.4, 128.4, 128.3, 125.7, 75.3, 46.4, 37.9, 32.1, 29.1, 28.5, 25.7, 25.5

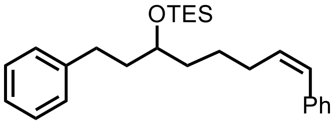

((1,8-Diphenyloct-7-en-3-yl)oxy)triethylsilane (33): Synthesized by the general procedure using 0.5 mmol dihydrocinnamaldehyde and 1.0 mmol **S1** and a reaction time of 16 hours. The crude residue was purified via column chromatography to afford the product as a mixture of *E* / *Z* isomers (8 : 92) determined by relative integrations of olefinic protons in 55% yield (average of two trials). The isomeric ratio of the coupled product was found to be unchanged from that of the starting material **S1** (*E* : *Z* 8 : 92).

^{1}H NMR (500 MHz, $CDCl_3$) δ 7.35 – 7.27 (m, 6H), 7.24 – 7.15 (m, 4H), 6.43 (d, *J* = 11.6 Hz, 0.9H, *Z*-isomer), 6.39 (d, *J* = 15.8 Hz, 0.1H, *E*-isomer), 6.22 (dt, *J* = 15.8, 6.9 Hz, 0.1H, *E*-isomer), 5.66 (dt, *J* = 11.6, 7.2 Hz, 0.9H, *Z*-isomer), 3.78 – 3.73 (m, 0.1H, *E*-isomer), 3.73 – 3.68 (m, 0.9H, *Z*-isomer), 2.73 – 2.55 (m, 2H), 2.38 – 2.30 (m, 1.8H, *Z*-isomer), 2.25 – 2.19 (m, 0.2H, *E*-isomer), 1.83 – 1.68 (m, 2H), 1.55 – 1.45 (m, 4H), 0.96 (t, *J* = 7.9 Hz, 9H), 0.60 (q, *J* = 7.9 Hz, 6H); **^{13}C NMR** (126 MHz, $CDCl_3$) δ 142.6, 137.7, 132.8, 129.0, 128.7, 128.32, 128.31, 128.1, 126.4, 125.6, 71.7, 36.7, 31.7, 28.6, 25.7, 6.9, 5.1; **HRMS (CI):** *m/z* calculated for [M+H]: 395.2770 found: 395.2756

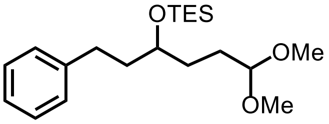

((6,6-Dimethoxy-1-phenylhexan-3-yl)oxy)triethylsilane (34): Synthesized by the general procedure using 0.5 mmol dihydrocinnamaldehyde and 1.0 mmol 3-bromopropionaldehyde dimethyl acetal and a reaction time of 5 hours. The crude residue was purified via column chromatography to afford the product as a light-yellow oil in 70% yield (average of two trials).

^{1}H NMR (500 MHz, $CDCl_3$) δ 7.30 – 7.25 (m, 2H), 7.20 – 7.15 (m, 3H), 4.36 (t, *J* = 5.6 Hz, 1H), 3.77 – 3.71 (m, 1H), 3.32 (s, 3H), 3.32 (s, 3H), 2.73 – 2.57 (m, 2H), 1.82 – 1.48 (m, 6H), 0.97 (t, *J* = 7.9 Hz, 9H), 0.61 (q, *J* = 8.0 Hz, 6H); **^{13}C NMR** (126 MHz, $CDCl_3$) δ 142.5, 128.33, 128.30, 125.6, 104.6, 71.5, 52.6, 52.5, 39.0, 31.7, 31.6, 28.3, 6.9, 5.1; **HRMS (CI+):** *m/z* calculated for [M-CH_3+H]: 337.2199 found: 337.2174

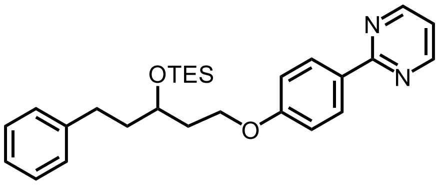

2-(4-((5-Phenyl-3-((triethylsilyl)oxy)pentyl)oxy)phenyl)pyrimidine (35): Synthesized by the general procedure using 0.2 mmol dihydrocinnamaldehyde and 0.2 mmol **S3** and a reaction time of 16 hours. The crude residue was purified via column chromatography to afford the product as a light-yellow oil in 61% yield (average of two trials).

^{1}H NMR (500 MHz, $CDCl_3$) δ 8.76 (d, *J* = 4.8 Hz, 2H), 8.38 (d, *J* = 8.5 Hz, 2H), 7.31 – 7.26 (m, 2H), 7.22 – 7.16 (m, 3H), 7.11 (t, *J* = 4.8 Hz, 2H), 6.98 (d, *J* = 8.5 Hz, 2H), 4.19 – 4.10 (m, 2H), 4.09 – 4.01 (m, 1H), 2.74 – 2.67 (m, 2H), 2.10 – 2.02 (m, 1H), 2.01 – 1.92 (m, 1H), 1.92 – 1.81 (m, 2H), 0.95 (t, *J* = 7.9 Hz, 9H), 0.60 (q, *J* = 7.9 Hz, 6H); **^{13}C NMR** (126 MHz, $CDCl_3$) δ 164.6, 161.3, 157.1, 142.3, 130.1, 129.7, 128.39, 128.33, 125.7, 118.2, 114.3, 68.6, 64.5, 39.5, 36.2, 31.5, 6.9, 5.0; **HRMS (ESI+):** *m/z* calculated for [M+H]: 449.2624 found: 449.2610

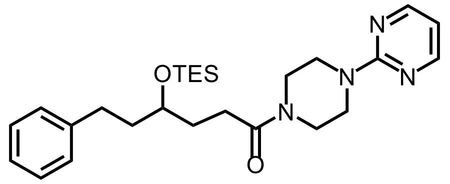

6-Phenyl-1-(4-(pyrimidin-2-yl)piperazin-1-yl)-4-((triethylsilyl)oxy)hexan-1-one (36): Synthesized by the general procedure using 0.2 mmol dihydrocinnamaldehyde and 0.2 mmol **S2** and a reaction time of 16 hours. The crude residue was purified via column chromatography to afford the product as a light-yellow oil in 34% yield (average of two trials).

^{1}H NMR (400 MHz, $CDCl_3$) δ 8.33 (d, *J* = 4.7 Hz, 2H), 7.32 – 7.24 (m, 2H), 7.21 – 7.15 (m, 3H), 6.54 (t, *J* = 4.7 Hz, 1H), 3.89 – 3.78 (m, 5H), 3.74 – 3.67 (m, 2H), 3.57 – 3.51 (m, 2H), 2.73 – 2.59 (m, 2H), 2.54 – 2.36 (m, 2H), 2.01 – 1.91 (m, 1H), 1.86 – 1.73 (m, 3H), 0.97 (t, *J* = 7.9 Hz, 9H), 0.61 (q, *J* = 7.7 Hz, 6H); **^{13}C NMR** (100 MHz, $CDCl_3$) δ 171.7, 161.5, 157.7, 142.3, 128.37, 128.30, 125.7, 110.4, 71.1, 45.2, 43.7, 43.5, 41.3, 39.1, 32.0, 31.7, 28.9, 6.9, 5.1; **HRMS (ESI+):** *m/z* calculated for [M+H]: 469.2999 found: 469.3003

VI. References

(1) Wenker, H. Syntheses from Ethanolamine. V. Synthesis of Δ^2-Oxazoline and of 2,2'-Δ^2-Dioxazoline. *J. Am. Chem. Soc.* **1938**, *60* (9), 2152–2153. https://doi.org/10.1021/ja01276a036.

(2) Bordwell, F. G.; Brannen, W. T. The Effect of the Carbonyl and Related Groups on the Reactivity of Halides in SN2 Reactions. *J. Am. Chem. Soc.* **1964**, *86* (21), 4645–4650. https://doi.org/10.1021/ja01075a025.

(3) Ogoshi, S.; Kamada, H.; Kurosawa, H. Reaction of (H2-Arylaldehyde)Nickel(0) Complexes with Me3SiX (X=OTf, Cl). Application to Catalytic Reductive Homocoupling Reaction of Arylaldehyde. *Tetrahedron* **2006**, *62* (32), 7583–7588. https://doi.org/10.1016/j.tet.2006.03.124.

(4) Lu, S.-C.; Wei, S.-C.; Wang, W.-X.; Zhang, W.; Tu, Z.-F. One-Pot Synthesis of Fused Benzo[c]Carbazoles by Photochemical Intramolecular Annulation of 3-Acyl-2-Haloindoles with Tethered Styrenes. *Eur. J. Org. Chem.* **2011**, *2011* (29), 5905–5910. https://doi.org/10.1002/ejoc.201100876.

(5) Pasquinucci, L.; Prezzavento, O.; Marrazzo, A.; Amata, E.; Ronsisvalle, S.; Georgoussi, Z.; Fourla, D.-D.; Scoto, G. M.; Parenti, C.; Aricò, G.; Ronsisvalle, G. Evaluation of N-Substitution in 6,7-Benzomorphan Compounds. *Bioorganic & Medicinal Chemistry* **2010**, *18* (14), 4975–4982. https://doi.org/10.1016/j.bmc.2010.06.005.

(6) Song, B.; Zheng, X.; Mo, J.; Xu, B. Palladium-Catalyzed Monoselective Halogenation of C□H Bonds: Efficient Access to Halogenated Arylpyrimidines Using Calcium Halides. *Adv. Synth. Catal.* **2010**, *352* (2–3), 329–335. https://doi.org/10.1002/adsc.200900778.

(7) Han, Y.; Zheng, B.; Peng, Y. Construction of Chiral 2-Substituted Octahydroindoles from Cyclic Ketones and Nitroolefins Bearing Only One α-Substituent. *Adv. Synth. Catal.* **2015**, *357* (6), 1136–1142. https://doi.org/10.1002/adsc.201400851.

(8) Machida, S.; Kato, N.; Harada, K.; Ohkanda, J. Bivalent Inhibitors for Disrupting Protein Surface-Substrate Interactions and for Dual Inhibition of Protein Prenyltransferases. *J. Am. Chem. Soc.* **2011**, *133* (4), 958–963. https://doi.org/10.1021/ja1086112.

(9) Demko, Z. P.; Sharpless, K. B. An Intramolecular [2 + 3] Cycloaddition Route to Fused 5-Heterosubstituted Tetrazoles. *Org. Lett.* **2001**, *3* (25), 4091–4094. https://doi.org/10.1021/ol010220x.

(10) Andersson, I. E.; Batsalova, T.; Haag, S.; Dzhambazov, B.; Holmdahl, R.; Kihlberg, J.; Linusson, A. (*E*)-Alkene and Ethylene Isosteres Substantially Alter the Hydrogen-Bonding Network in Class II MHC A^q /Glycopeptide Complexes and Affect T-Cell Recognition. *J. Am. Chem. Soc.* **2011**, *133* (36), 14368–14378. https://doi.org/10.1021/ja2038722.

(11) Wolf, N.; Kersting, L.; Herok, C.; Mihm, C.; Seibel, J. High-Yielding Water-Soluble Asymmetric Cyanine Dyes for Labeling Applications. *J. Org. Chem.* **2020**, *85* (15), 9751–9760. https://doi.org/10.1021/acs.joc.0c01084.

(12) Bassetto, M.; Leyssen, P.; Neyts, J.; Yerukhimovich, M. M.; Frick, D. N.; Courtney-Smith, M.; Brancale, A. In Silico Identification, Design and Synthesis of Novel Piperazine-Based Antiviral Agents Targeting the Hepatitis C Virus Helicase. *European Journal of Medicinal Chemistry* **2017**, *125*, 1115–1131. https://doi.org/10.1016/j.ejmech.2016.10.043.

(13) Zhong, C.; Wang, Y.; Hung, A. W.; Schreiber, S. L.; Young, D. W. Diastereoselective Control of Intramolecular Aza-Michael Reactions Using Achiral Catalysts. *Org. Lett.* **2011**, *13* (20), 5556–5559. https://doi.org/10.1021/ol202276h.
(14) Bedford, R. B.; Brenner, P. B.; Carter, E.; Gallagher, T.; Murphy, D. M.; Pye, D. R. Iron-Catalyzed Borylation of Alkyl, Allyl, and Aryl Halides: Isolation of an Iron(I) Boryl Complex. *Organometallics* **2014**, *33* (21), 5940–5943. https://doi.org/10.1021/om500847j.
(15) He, W.; Huang, J.; Sun, X.; Frontier, A. J. Total Synthesis of (±)-Merrilactone A. *J. Am. Chem. Soc.* **2008**, *130* (1), 300–308. https://doi.org/10.1021/ja0761986.
(16) Zhou, S.; Wang, S.; Wang, J.; Nian, Y.; Peng, P.; Soloshonok, V. A.; Liu, H. Configurationally Stable (*S*)- and (*R*)-α-Methylproline-Derived Ligands for the Direct Chemical Resolution of Free Unprotected β ^{3} -Amino Acids: Configurationally Stable (*S*)- and (*R*)-α-Methylproline-Derived Ligands for the Direct Chemical Resolution of Free Unprotected β ^{3} -Amino Acids. *Eur. J. Org. Chem.* **2018**, *2018* (15), 1821–1832. https://doi.org/10.1002/ejoc.201800120.
(17) Perlmutter, P.; Selajerern, W.; Vounatsos, F. Enantioselective Synthesis of the Dioxabicyclo[3.2.1]Octane Core of the Zaragozic Acids via Intramolecular Wacker-Type Cyclisation Reactions. *Org. Biomol. Chem.* **2004**, *2* (15), 2220. https://doi.org/10.1039/b406280a.
(18) Hu, B.; Li, Y.; Li, Z.; Meng, X. Aniline Mediated Oxidative C–C Bond Cleavage of α-Alkoxy Aldehydes in Air and a Model Reaction for the Synthesis of α-(d)-Amino Acid Derivatives. *Org. Biomol. Chem.* **2013**, *11* (25), 4138. https://doi.org/10.1039/c3ob40685g.
(19) Cahiez, G.; Duplais, C.; Buendia, J. Chemistry of Organomanganese(II) Compounds. *Chem. Rev.* **2009**, *109* (3), 1434–1476. https://doi.org/10.1021/cr800341a.
(20) Iovel, I.; Popelis, J.; Gaukhman, A.; Lukevics, E. Hydrosilylation of Unsaturated (Hetero)Aromatic Aldehydes and Related Compounds Catalyzed by Transition Metal Complexes. *Journal of Organometallic Chemistry* **1998**, 8.
(21) Zhang, Q.-C.; Wu, F.-T.; Hao, H.-M.; Xu, H.; Zhao, H.-X.; Long, L.-S.; Huang, R.-B.; Zheng, L.-S. Modulating the Rotation of a Molecular Rotor through Hydrogen-Bonding Interactions between the Rotator and Stator. *Angew. Chem. Int. Ed.* **2013**, 4.
(22) Fujioka, H.; Ohba, Y.; Hirose, H.; Murai, K.; Kita, Y. Mild and Efficient Removal of Hydroxyethyl Unit from 2-Hydroxyethyl Ether Derivatives Leading to Alcohols. *Org. Lett.* **2005**, *7* (15), 4.
(23) Harada, S.; Kowase, N.; Tabuchi, N.; Taguchi, T.; Dobashi, Y.; Dobashi, A.; Hanzawa, Y. Hydrozirconation of Alkenyloxirane Derivatives: Preparation of Cycloalkylmethanols. *Tetrahedron* **1998**, *54* (5–6), 753–766. https://doi.org/10.1016/S0040-4020(97)10340-4.

VII. NMR Spectra

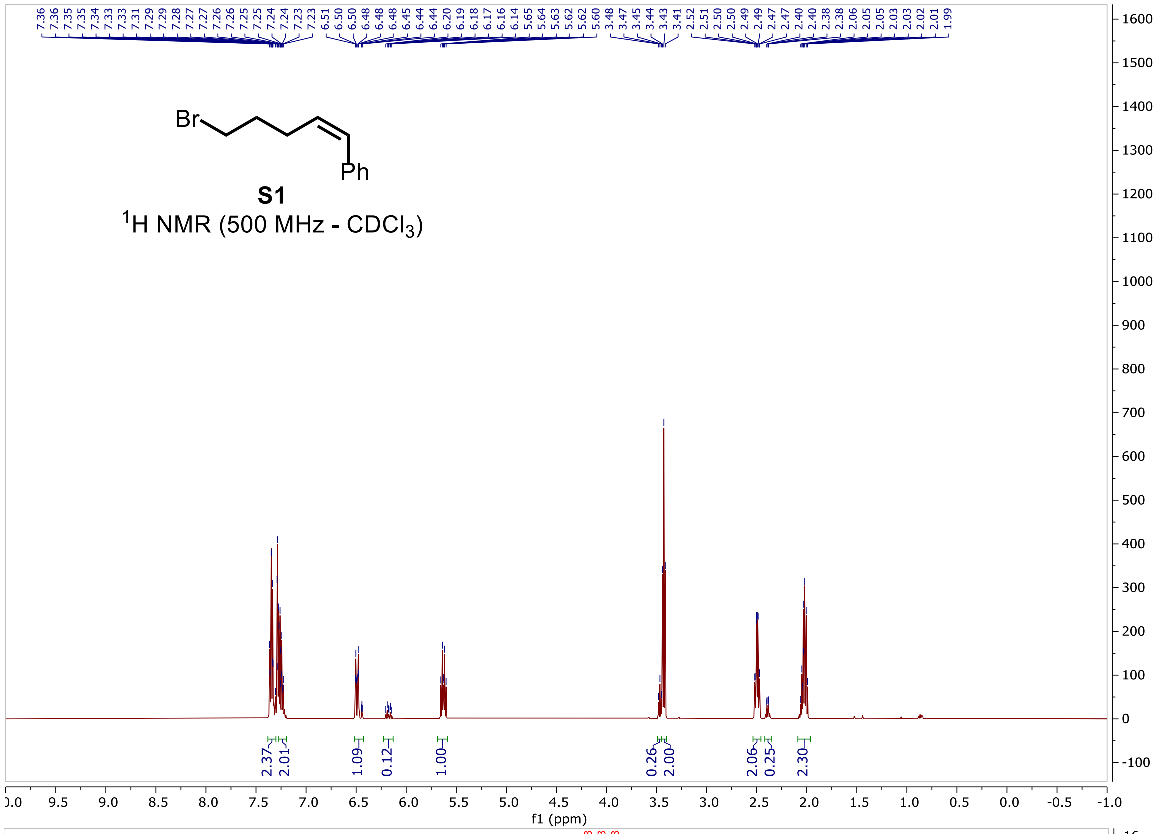

S1

^{1}H NMR (500 MHz - $CDCl_3$)

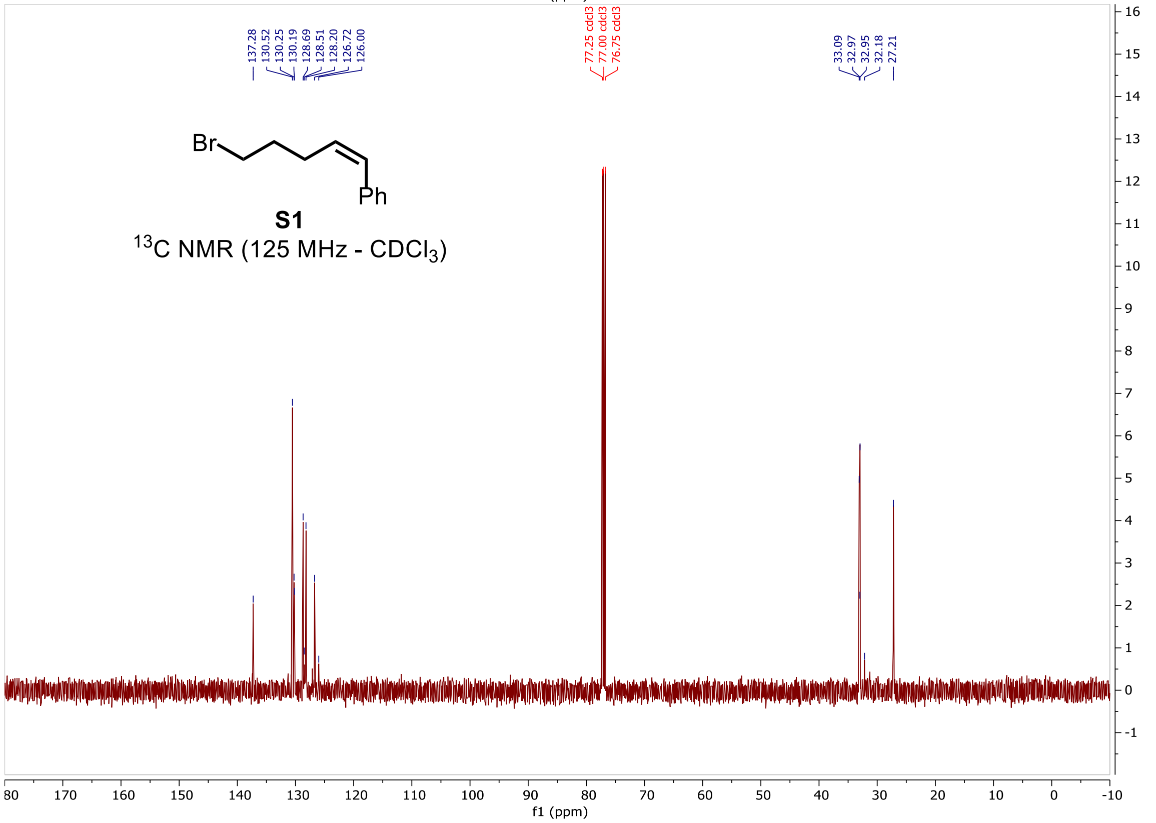

S1

^{13}C NMR (125 MHz - $CDCl_3$)

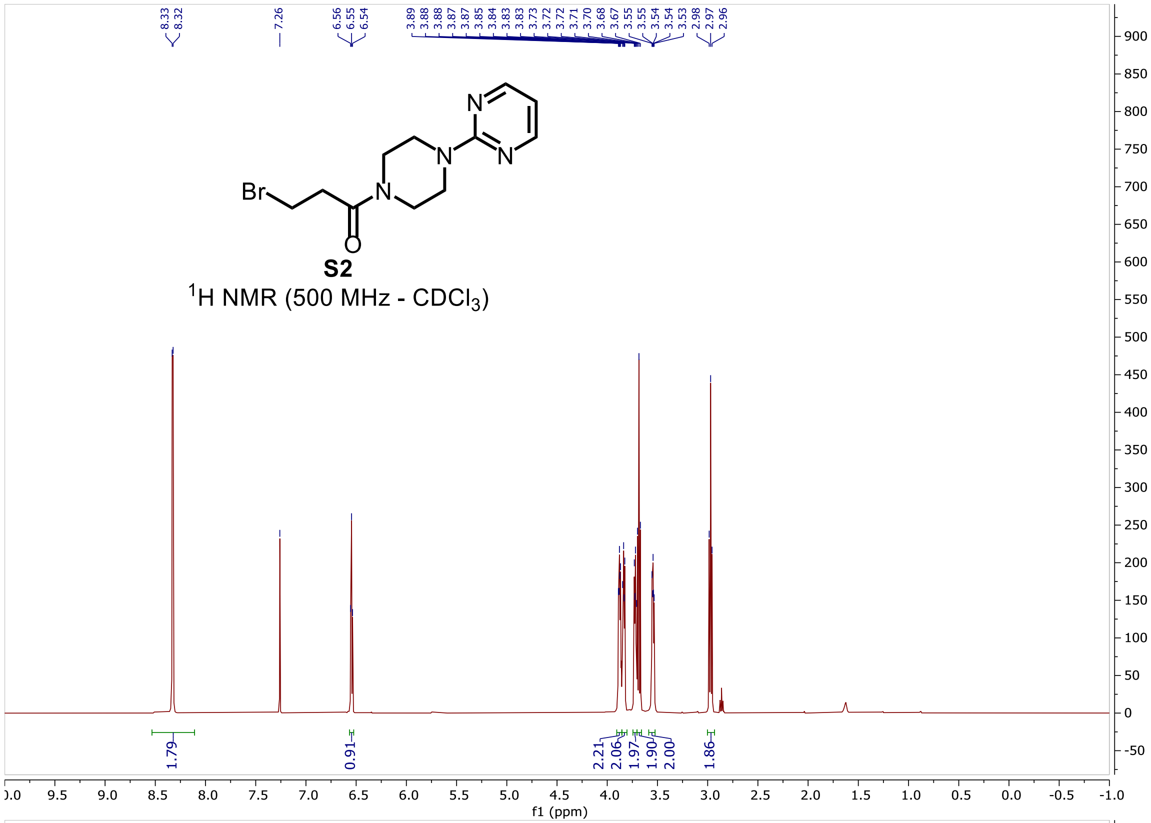
S2
^{1}H NMR (500 MHz - CDCl$_3$)
f1 (ppm)

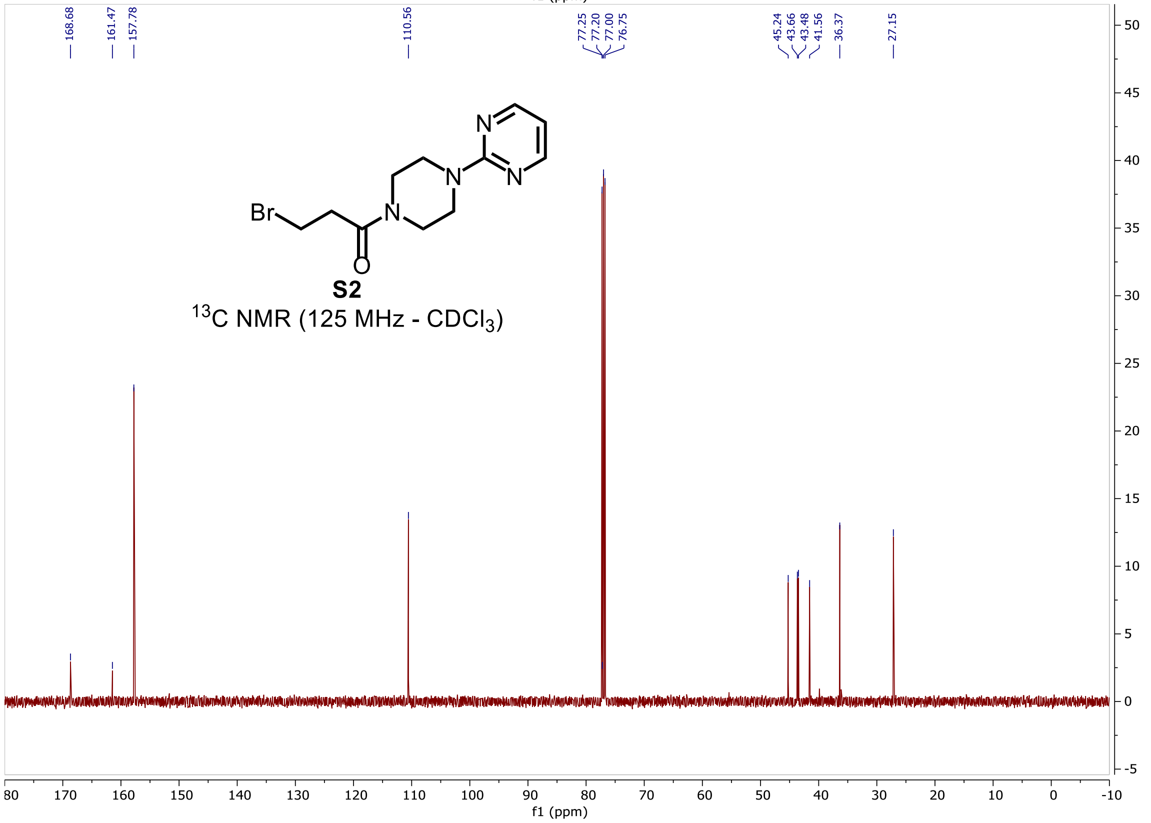
S2
^{13}C NMR (125 MHz - CDCl$_3$)
f1 (ppm)

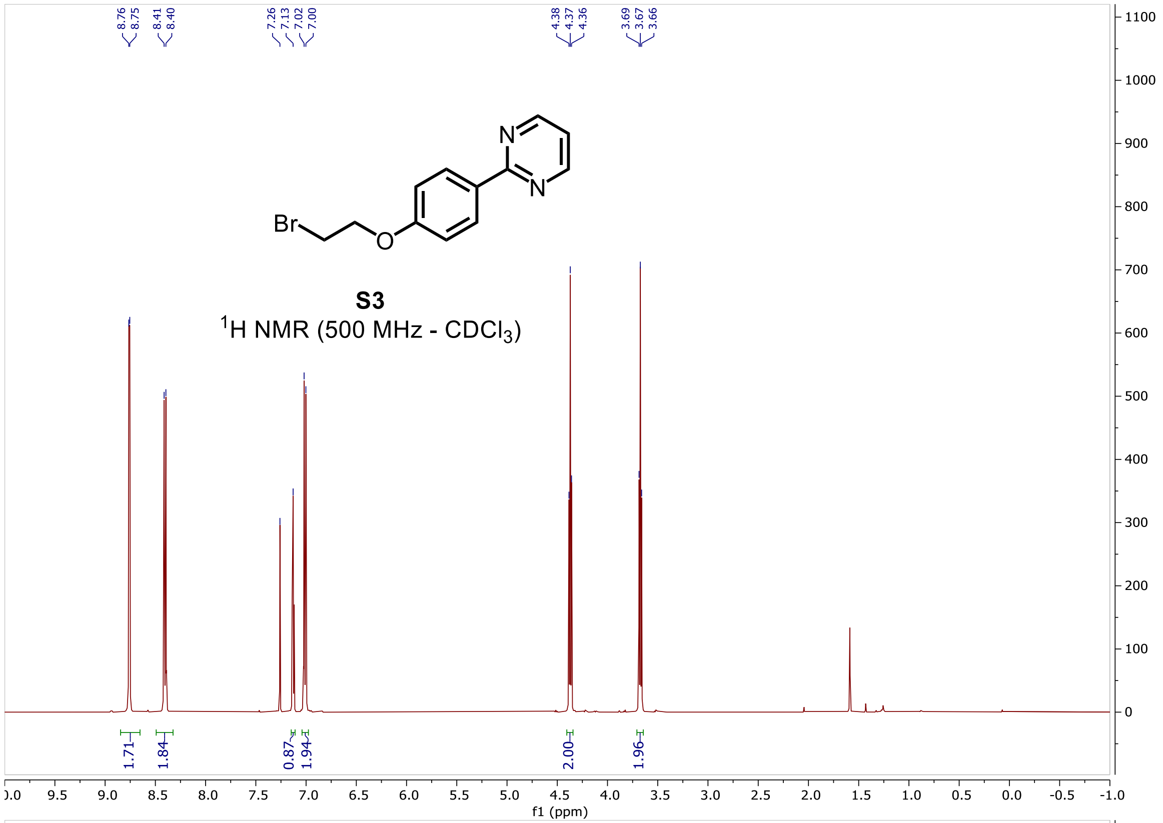
S3
1H NMR (500 MHz - CDCl3)
f1 (ppm)

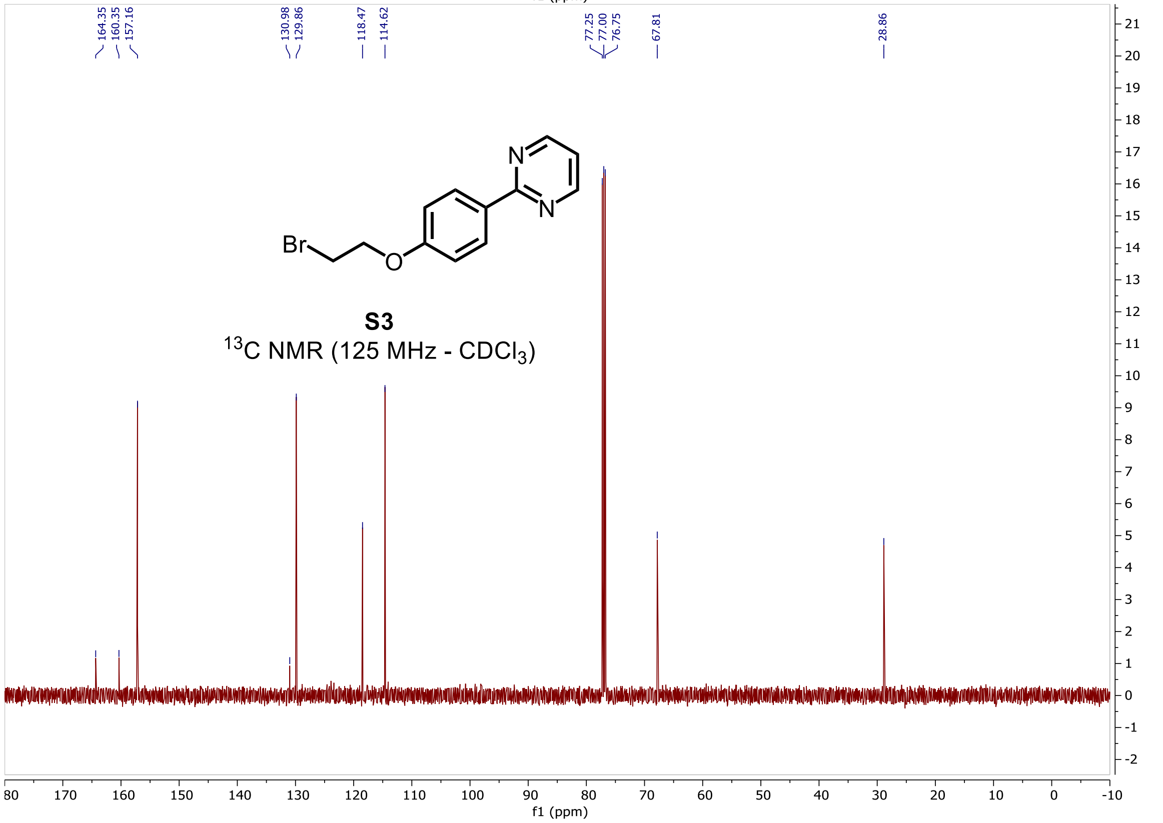
S3
13C NMR (125 MHz - CDCl3)
f1 (ppm)

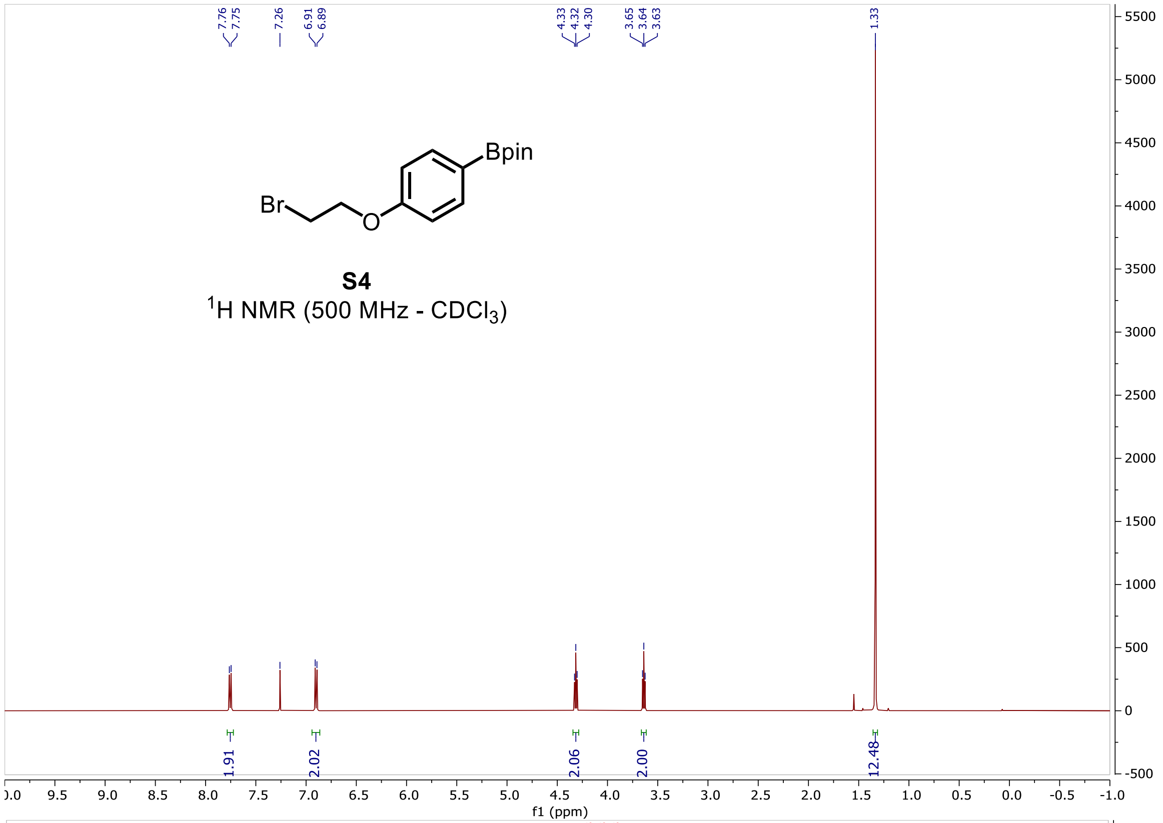
7.76
7.75
7.26
6.91
6.89
4.33
4.32
4.30
3.65
3.64
3.63
1.33
Bpin
Br
O
S4
1H NMR (500 MHz - CDCl3)
1.91
2.02
2.06
2.00
12.48
f1 (ppm)

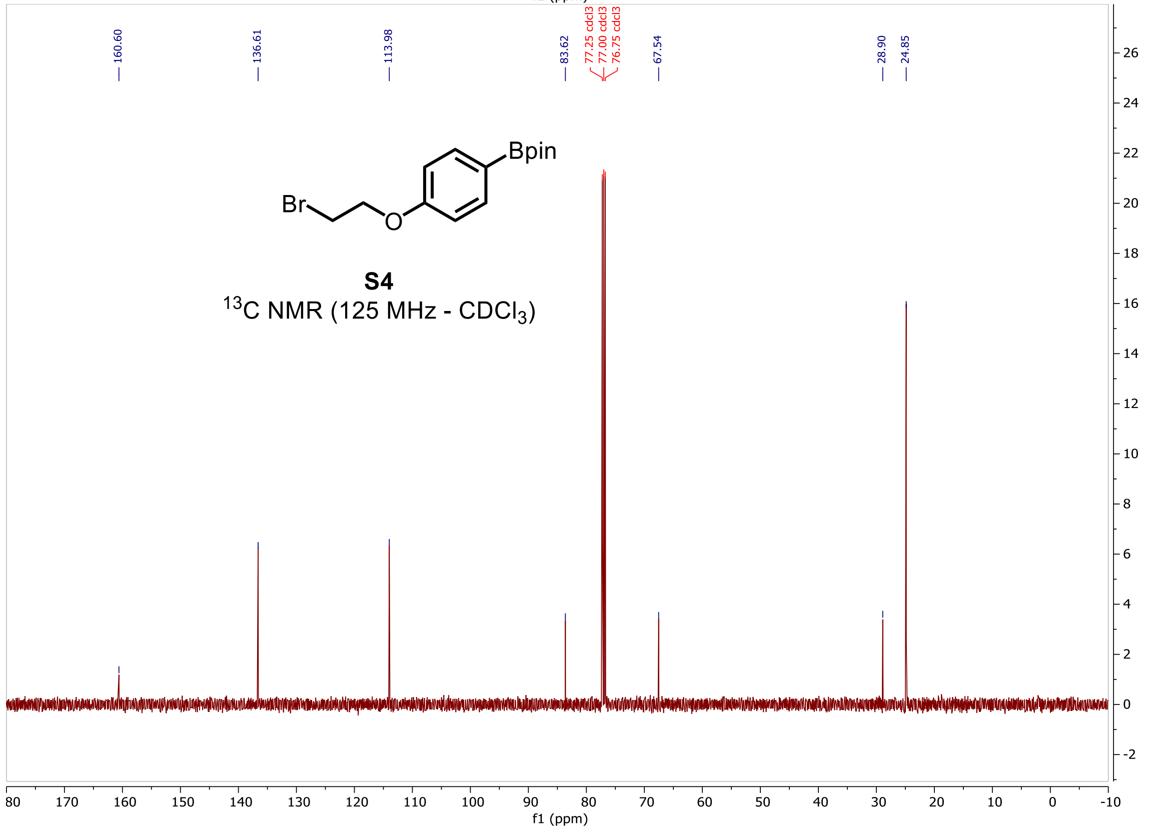
160.60
136.61
113.98
83.62
77.25 cdcl3
77.00 cdcl3
76.75 cdcl3
67.54
28.90
24.85
Bpin
Br
O
S4
13C NMR (125 MHz - CDCl3)
f1 (ppm)

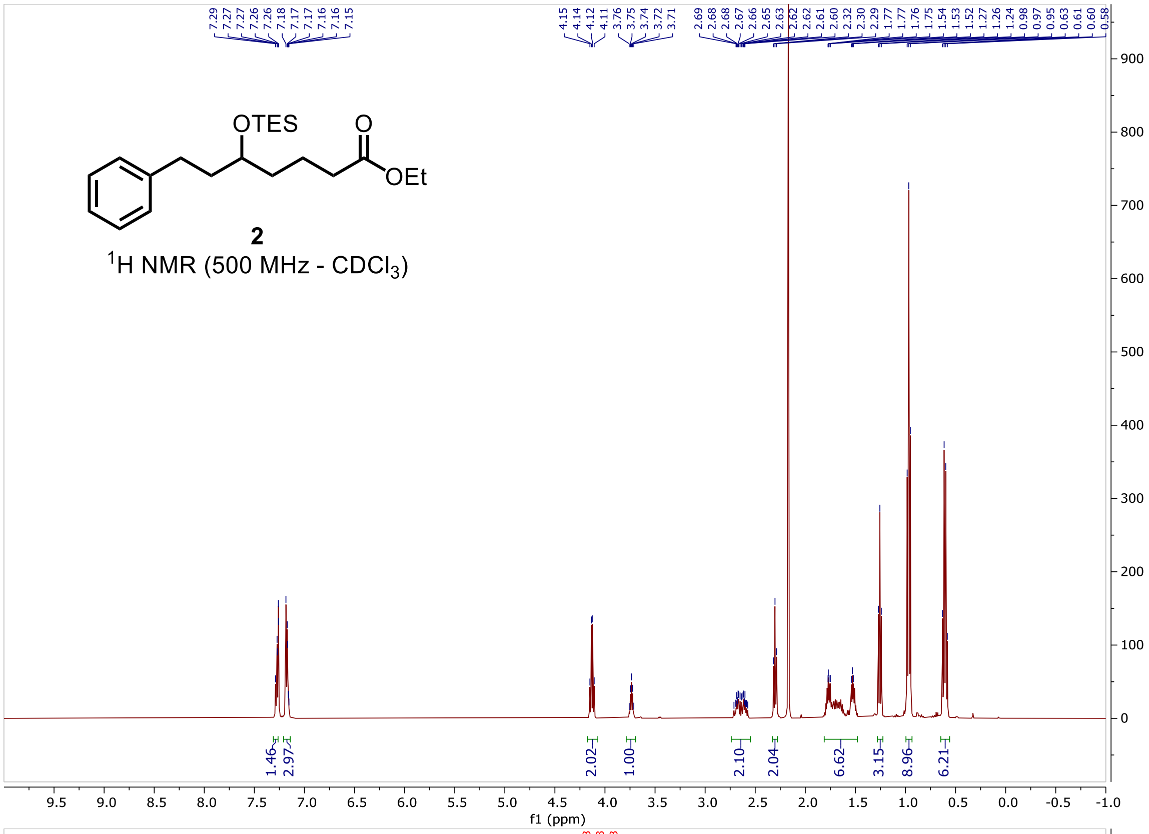

7.29
7.27
7.27
7.26
7.26
7.18
7.17
7.17
7.16
7.16
7.15
4.15
4.14
4.12
4.11
3.76
3.75
3.74
3.72
3.71
2.69
2.68
2.68
2.67
2.66
2.65
2.63
2.62
2.62
2.61
2.60
2.32
2.30
2.29
1.77
1.77
1.76
1.75
1.54
1.53
1.52
1.27
1.26
1.24
0.98
0.97
0.95
0.63
0.61
0.60
0.58
OTES
O
OEt
2
^{1}H NMR (500 MHz - $CDCl_3$)
1.46
2.97
2.02
1.00
2.10
2.04
6.62
3.15
8.96
6.21
f1 (ppm)

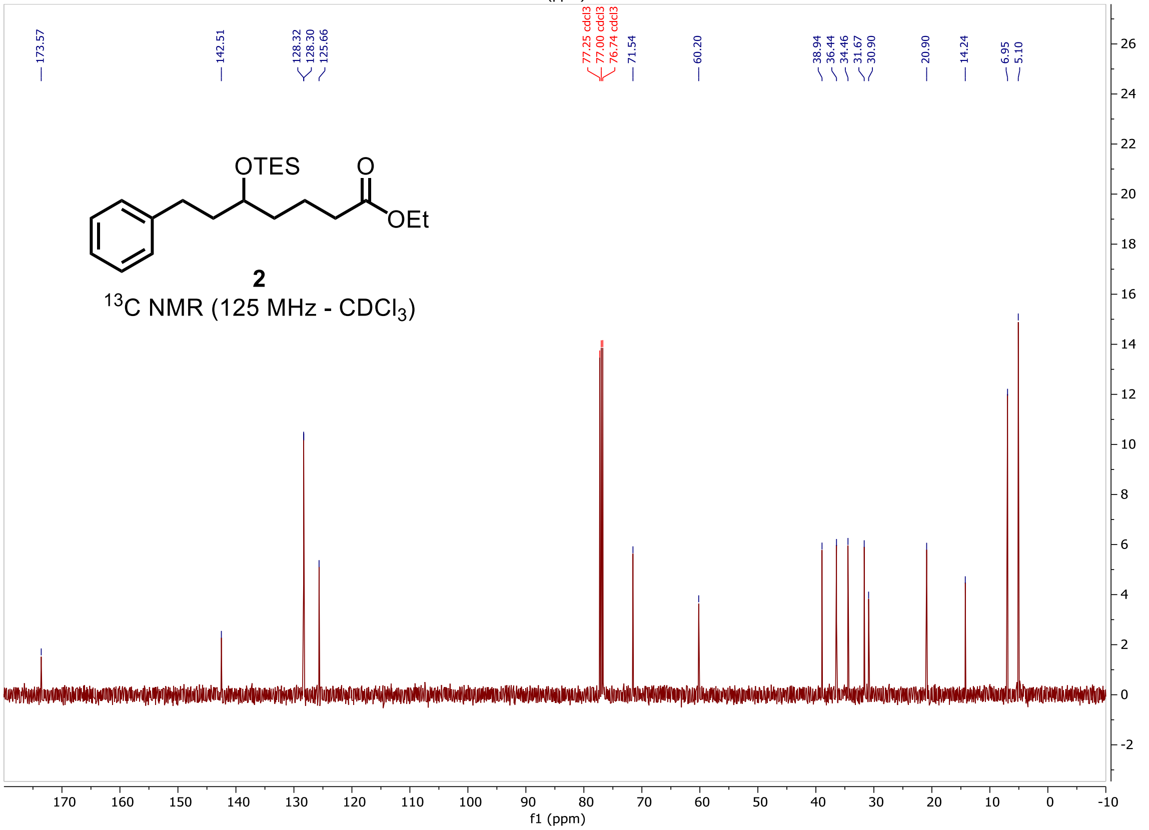

173.57
142.51
128.32
128.30
125.66
77.25 cdcl3
77.00 cdcl3
76.74 cdcl3
71.54
60.20
38.94
36.44
34.46
31.67
30.90
20.90
14.24
6.95
5.10
OTES
O
OEt
2
^{13}C NMR (125 MHz - $CDCl_3$)
f1 (ppm)

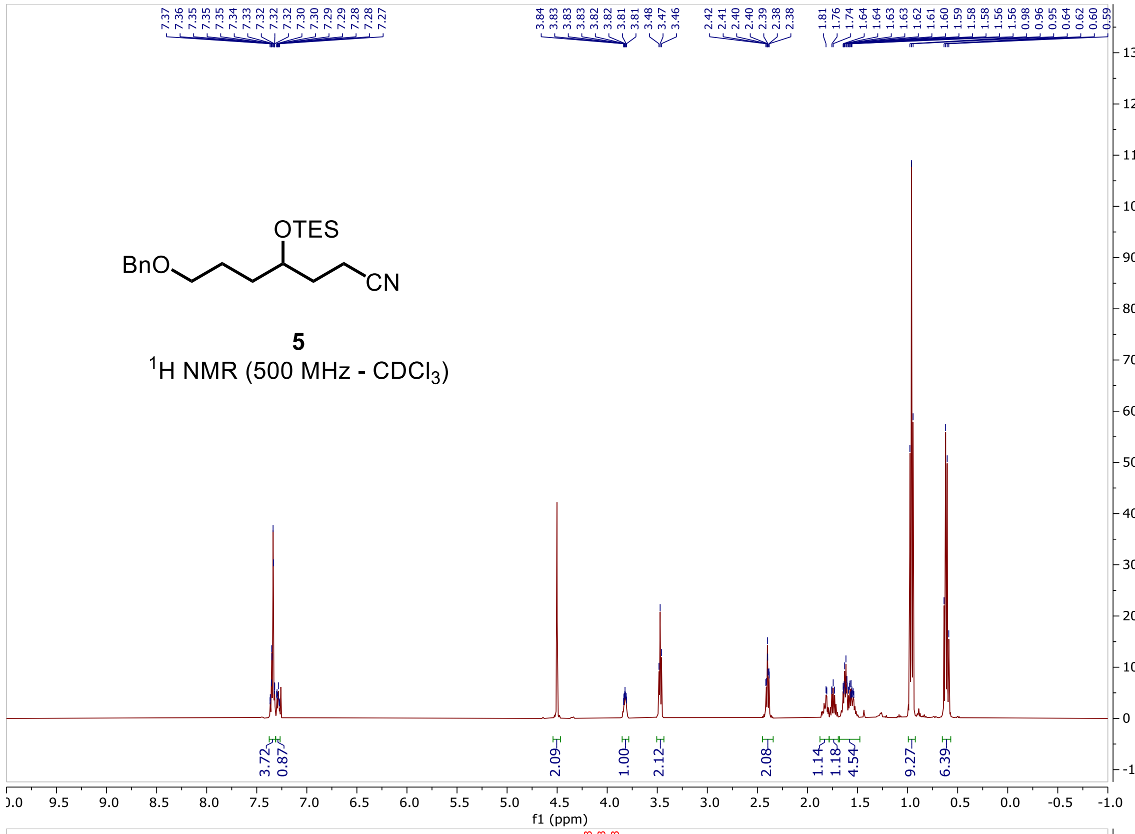
OTES
BnO
CN
5
1H NMR (500 MHz - CDCl3)
f1 (ppm)

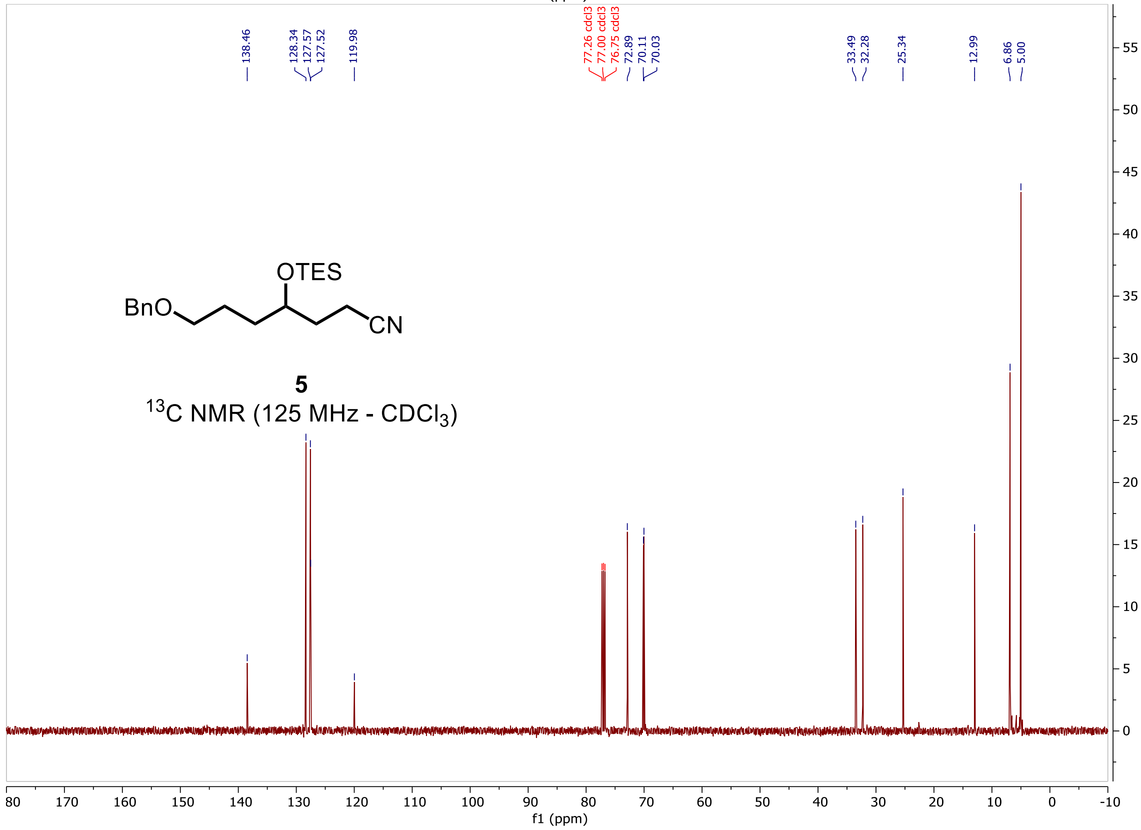
OTES
BnO
CN
5
13C NMR (125 MHz - CDCl3)
f1 (ppm)

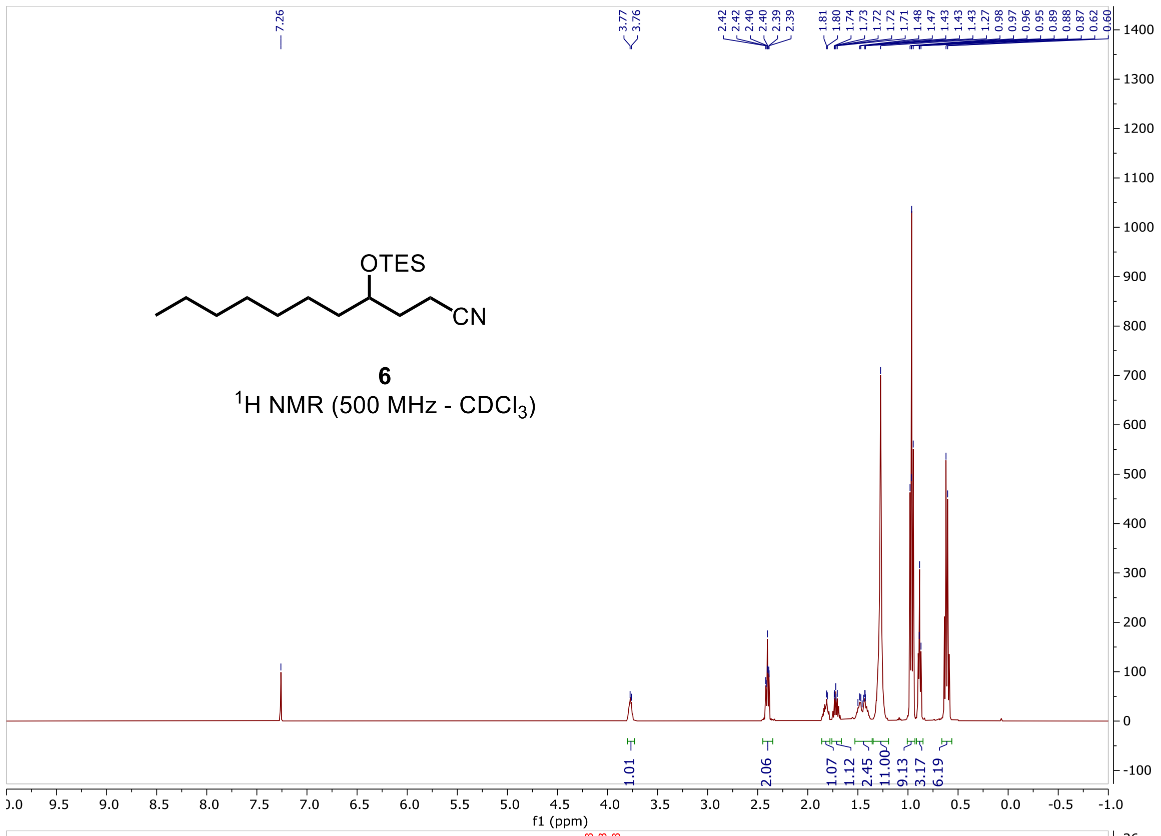
OTES
CN
6
1H NMR (500 MHz - CDCl3)
f1 (ppm)

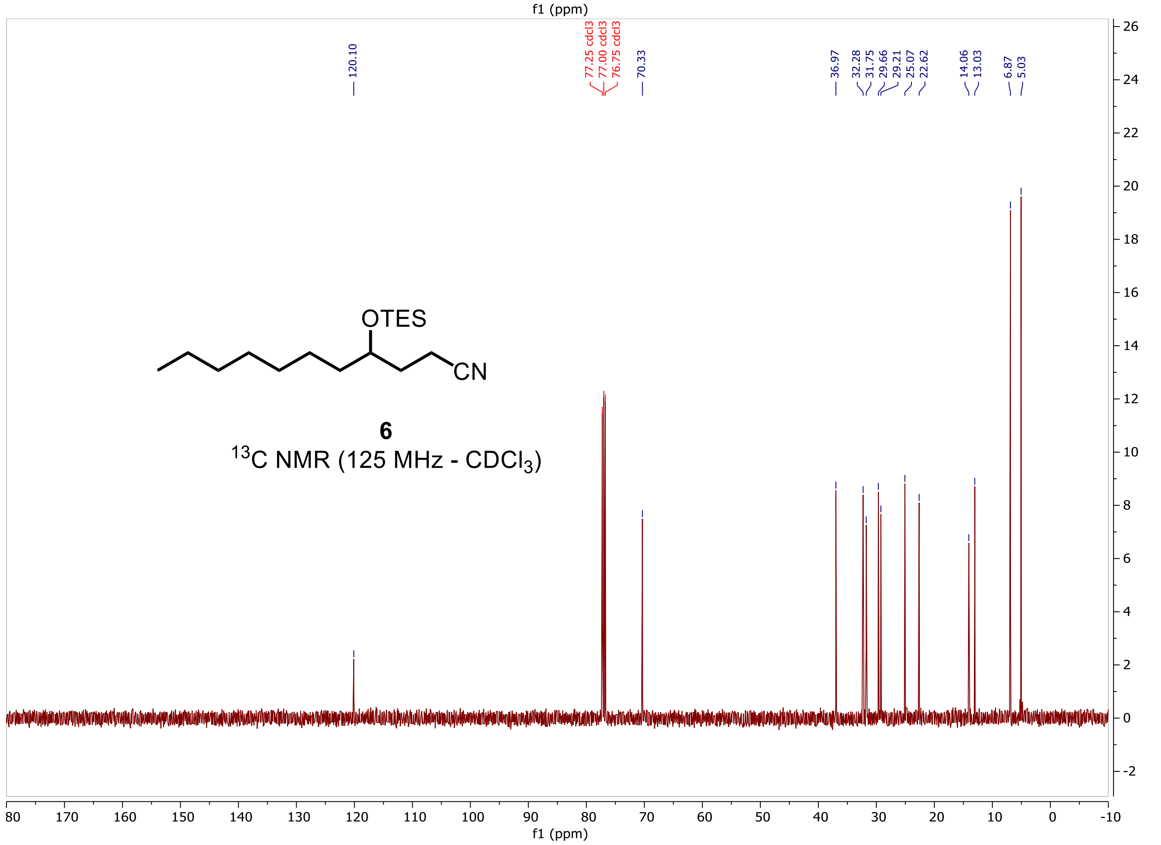
OTES
CN
6
13C NMR (125 MHz - CDCl3)
f1 (ppm)

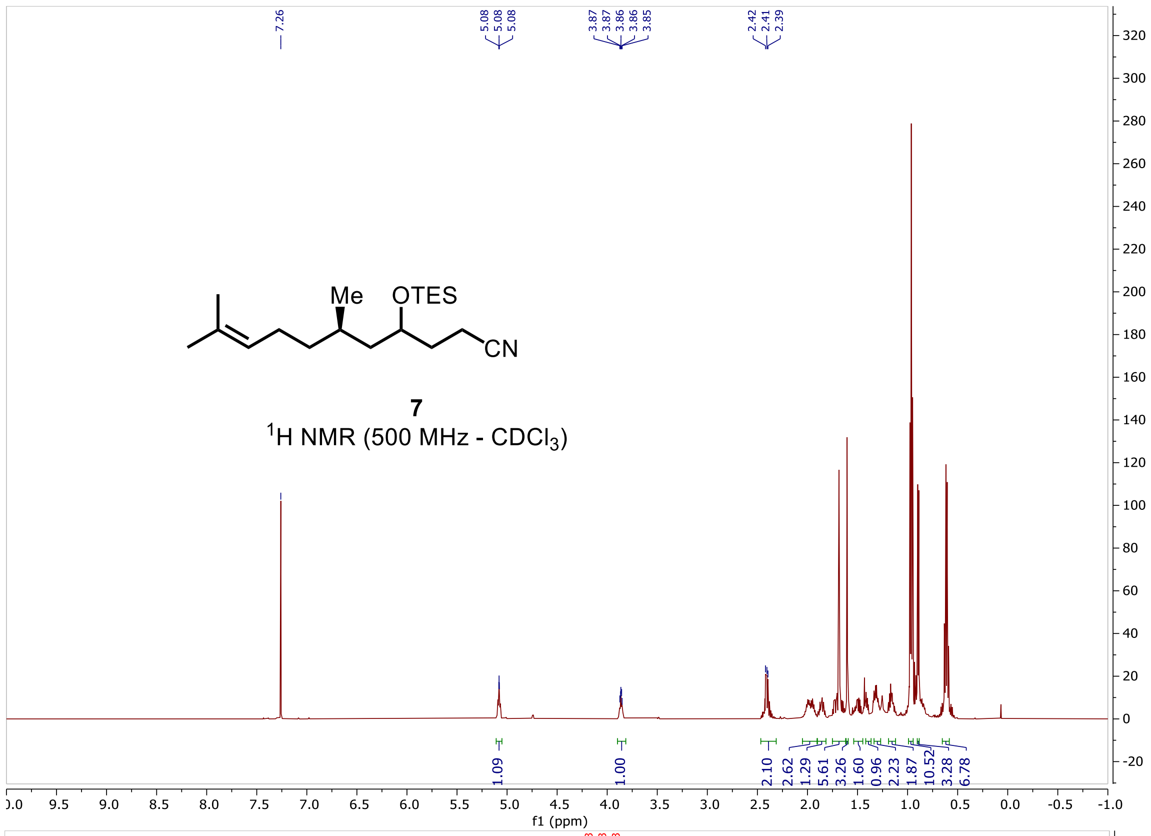
Me
OTES
CN
7
1H NMR (500 MHz - CDCl3)
f1 (ppm)

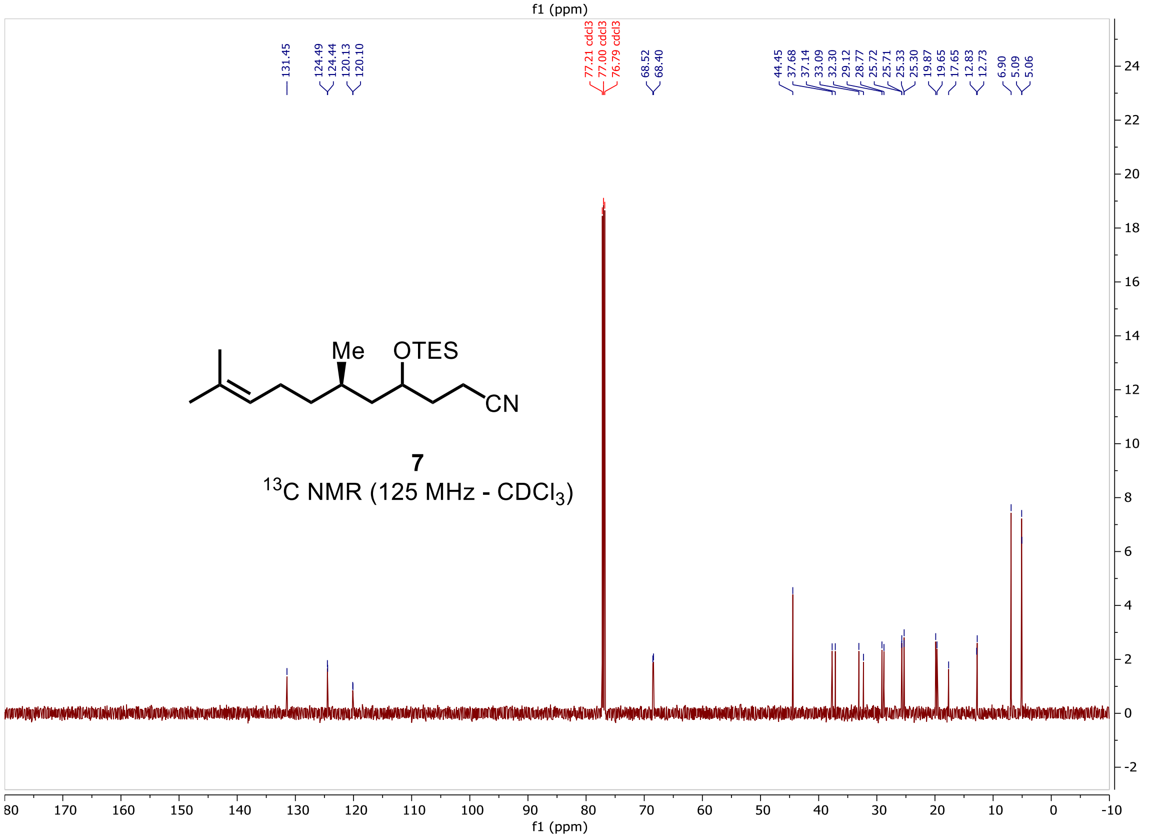
Me
OTES
CN
7
13C NMR (125 MHz - CDCl3)
f1 (ppm)

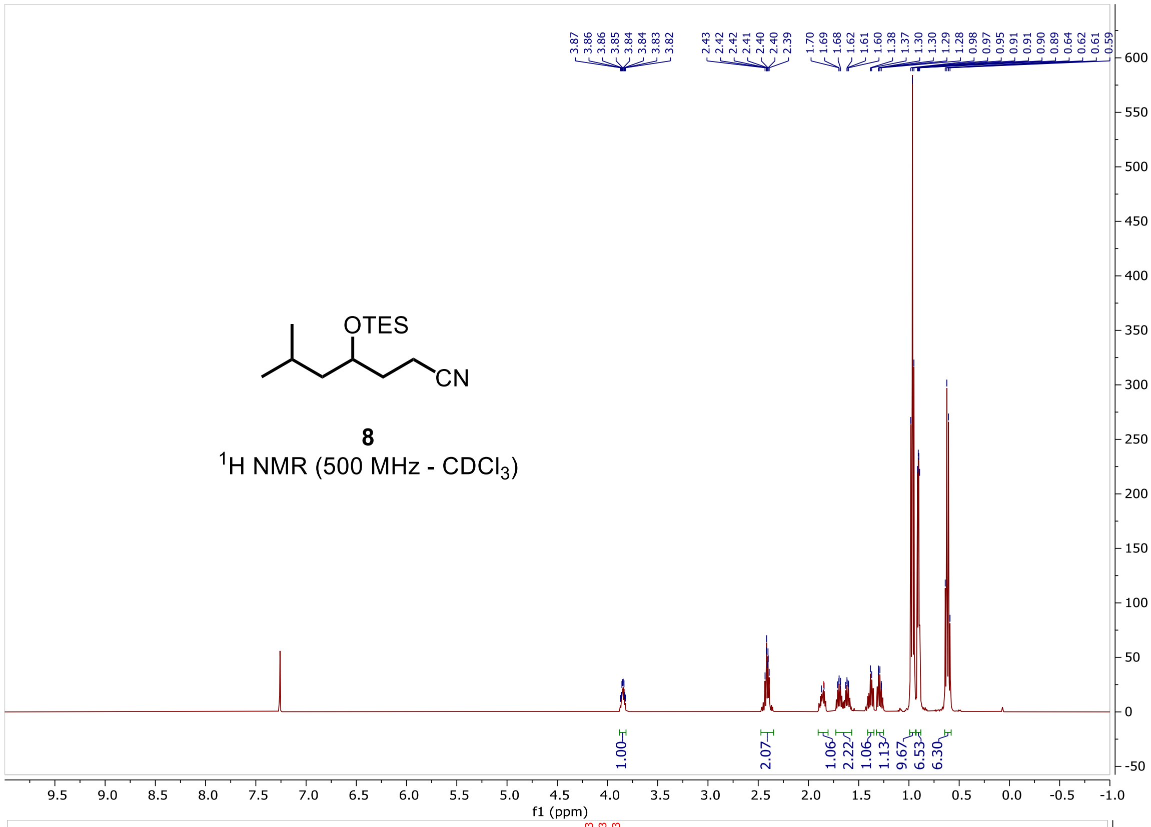
3.87
3.86
3.86
3.85
3.84
3.84
3.83
3.82
2.43
2.42
2.42
2.41
2.40
2.40
2.39
1.70
1.69
1.68
1.62
1.61
1.60
1.38
1.37
1.30
1.30
1.30
1.29
1.28
0.98
0.97
0.95
0.91
0.91
0.90
0.89
0.64
0.62
0.61
0.59
OTES
CN
8
1H NMR (500 MHz - CDCl3)
1.00
2.07
1.06
2.22
1.06
1.13
9.67
6.53
6.30
f1 (ppm)

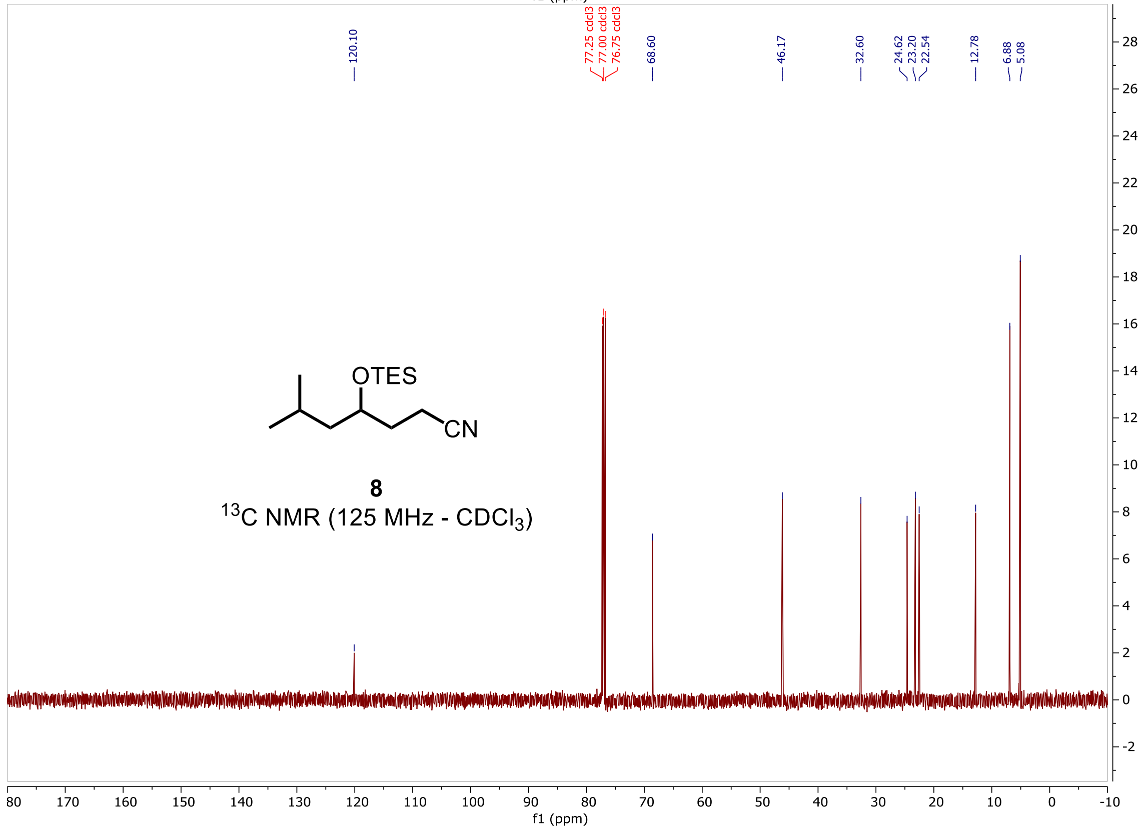
120.10
77.25 cdcl3
77.00 cdcl3
76.75 cdcl3
68.60
46.17
32.60
24.62
23.20
22.54
12.78
6.88
5.08
OTES
CN
8
13C NMR (125 MHz - CDCl3)
f1 (ppm)

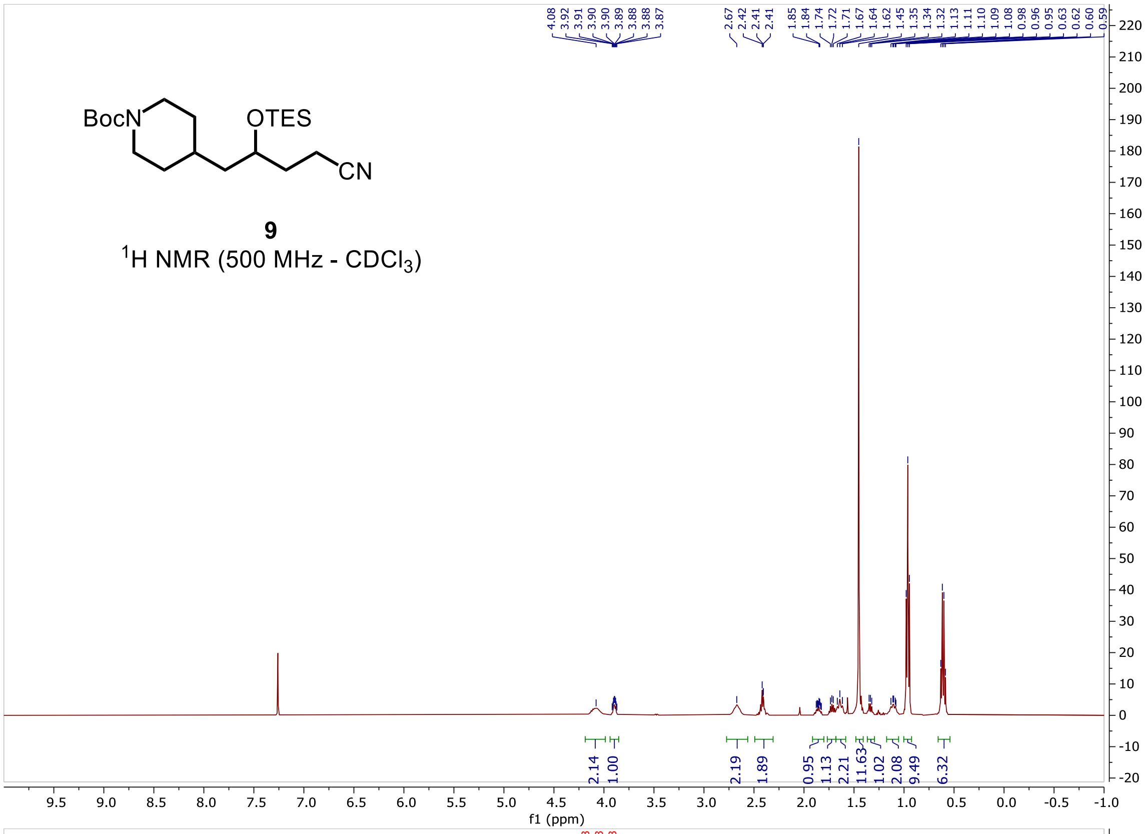

9

^{1}H NMR (500 MHz - $CDCl_3$)

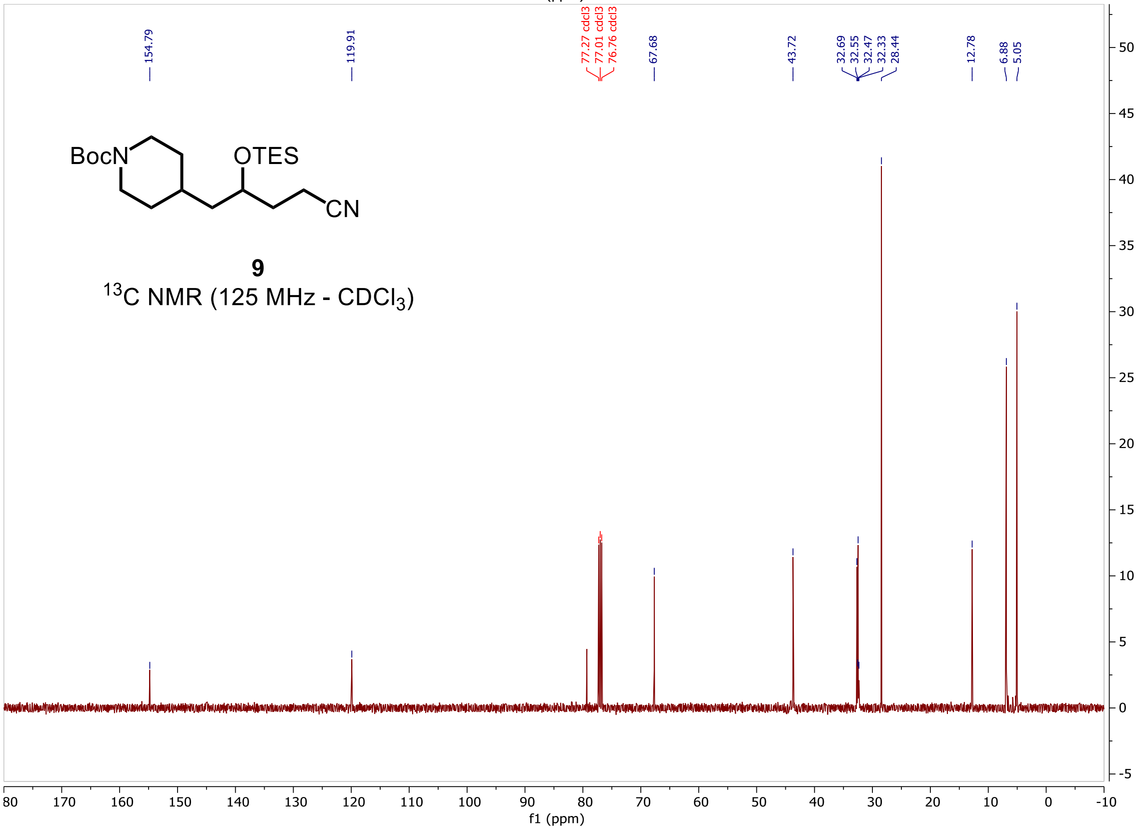

9

^{13}C NMR (125 MHz - $CDCl_3$)

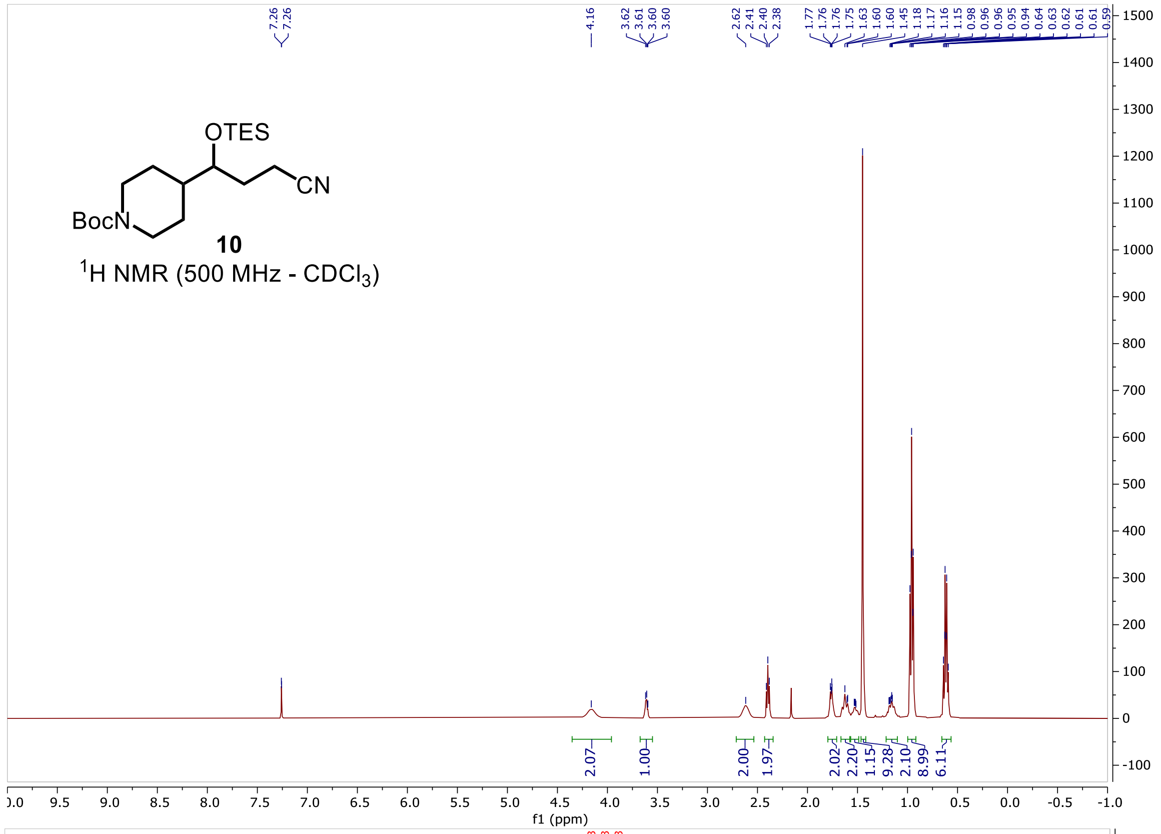
OTES
CN
BocN
10
1H NMR (500 MHz - CDCl3)
f1 (ppm)

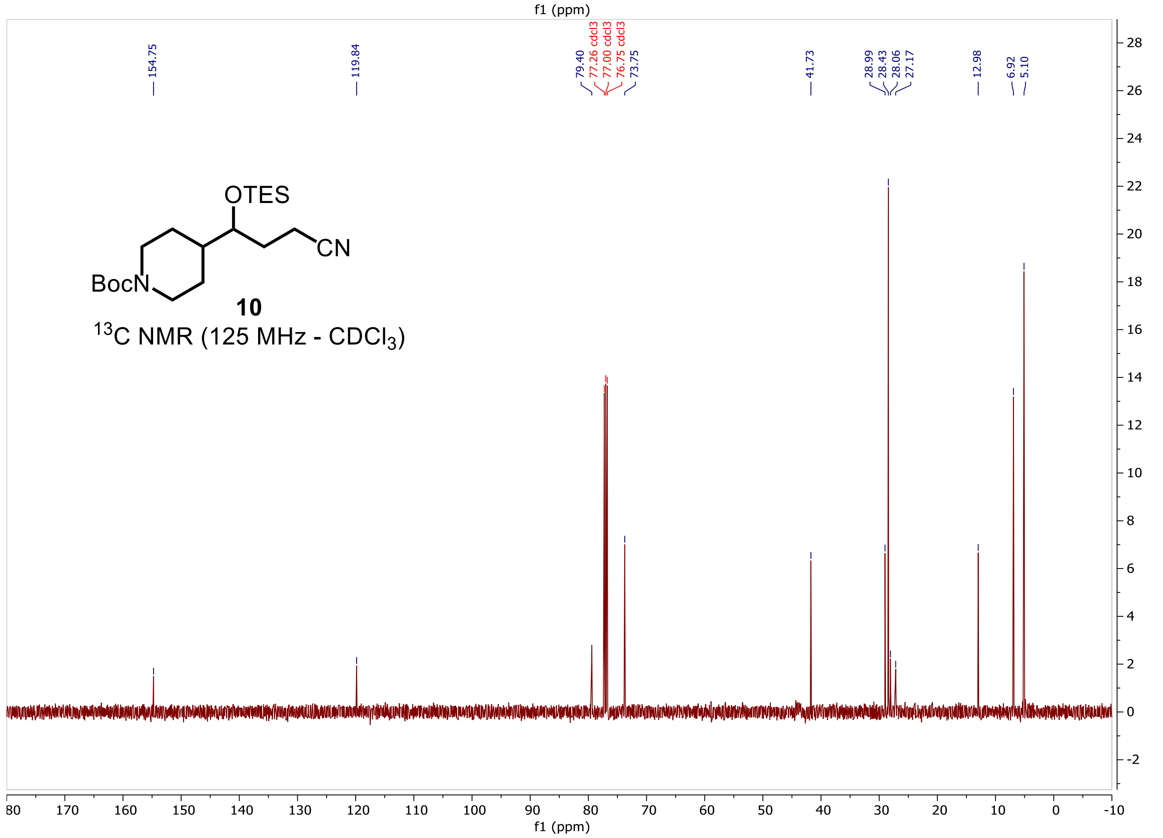
OTES
CN
BocN
10
13C NMR (125 MHz - CDCl3)
f1 (ppm)

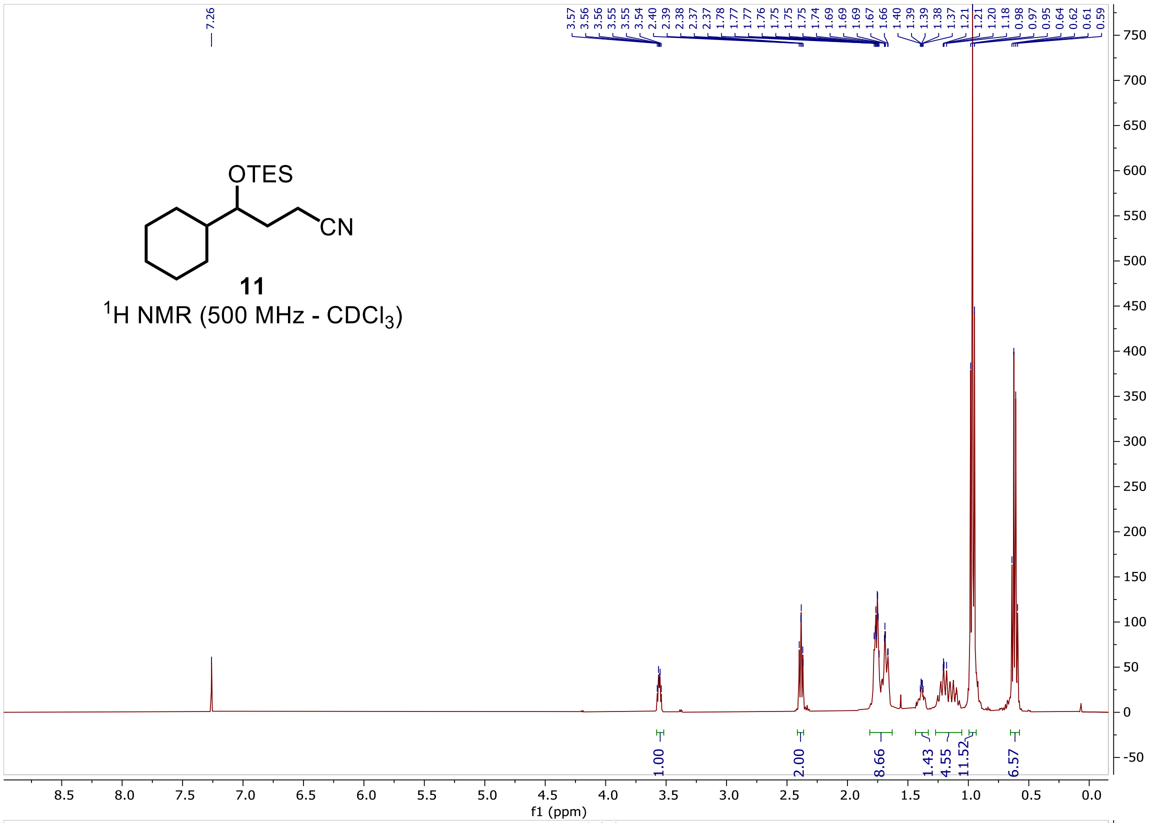
OTES
CN
11
^{1}H NMR (500 MHz - CDCl$_3$)
7.26
3.57 3.56 3.56 3.55 3.55 3.54 2.40 2.39 2.38 2.37 2.37 1.78 1.77 1.77 1.76 1.75 1.75 1.75 1.74 1.69 1.69 1.69 1.67 1.66 1.40 1.39 1.39 1.38 1.37 1.21 1.21 1.20 1.18 0.98 0.97 0.95 0.64 0.62 0.61 0.59
1.00
2.00
8.66
1.43
4.55
11.52
6.57
f1 (ppm)

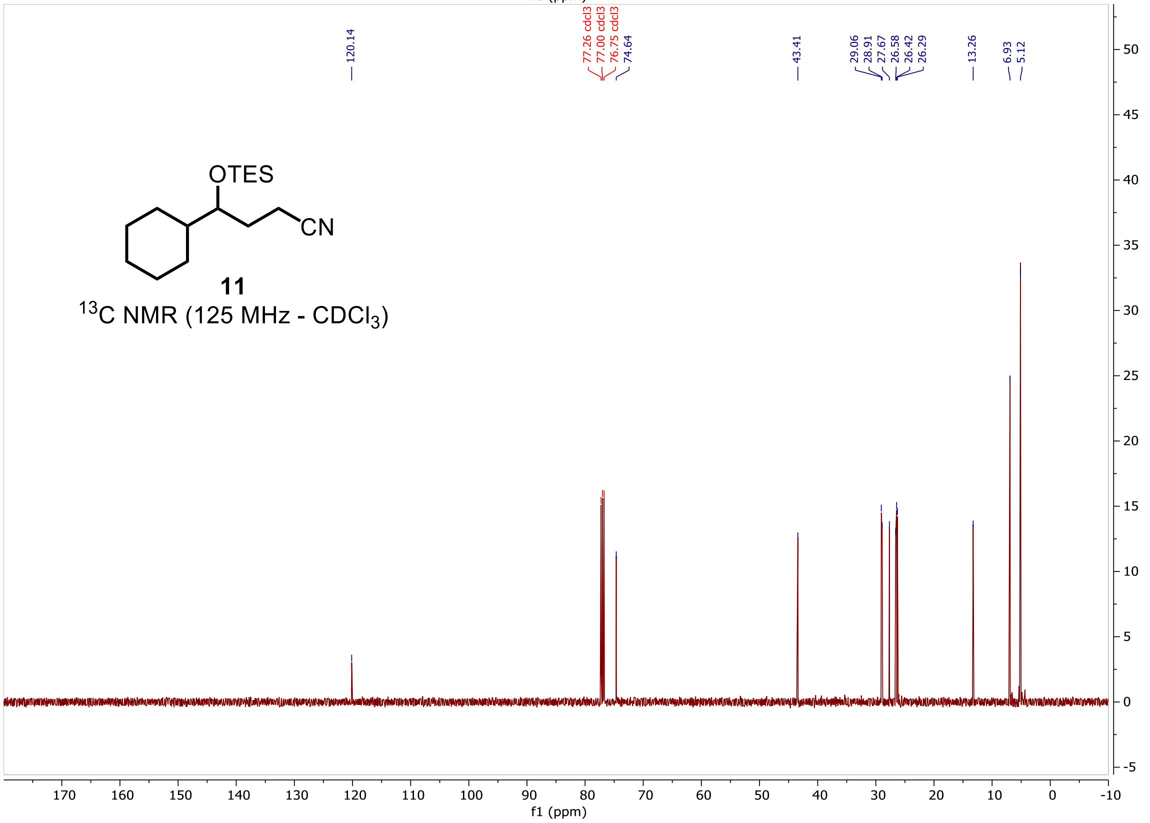
OTES
CN
11
^{13}C NMR (125 MHz - CDCl$_3$)
120.14
77.26 cdcl3
77.00 cdcl3
76.75 cdcl3
74.64
43.41
29.06 28.91 27.67 26.58 26.42 26.29
13.26
6.93
5.12
f1 (ppm)

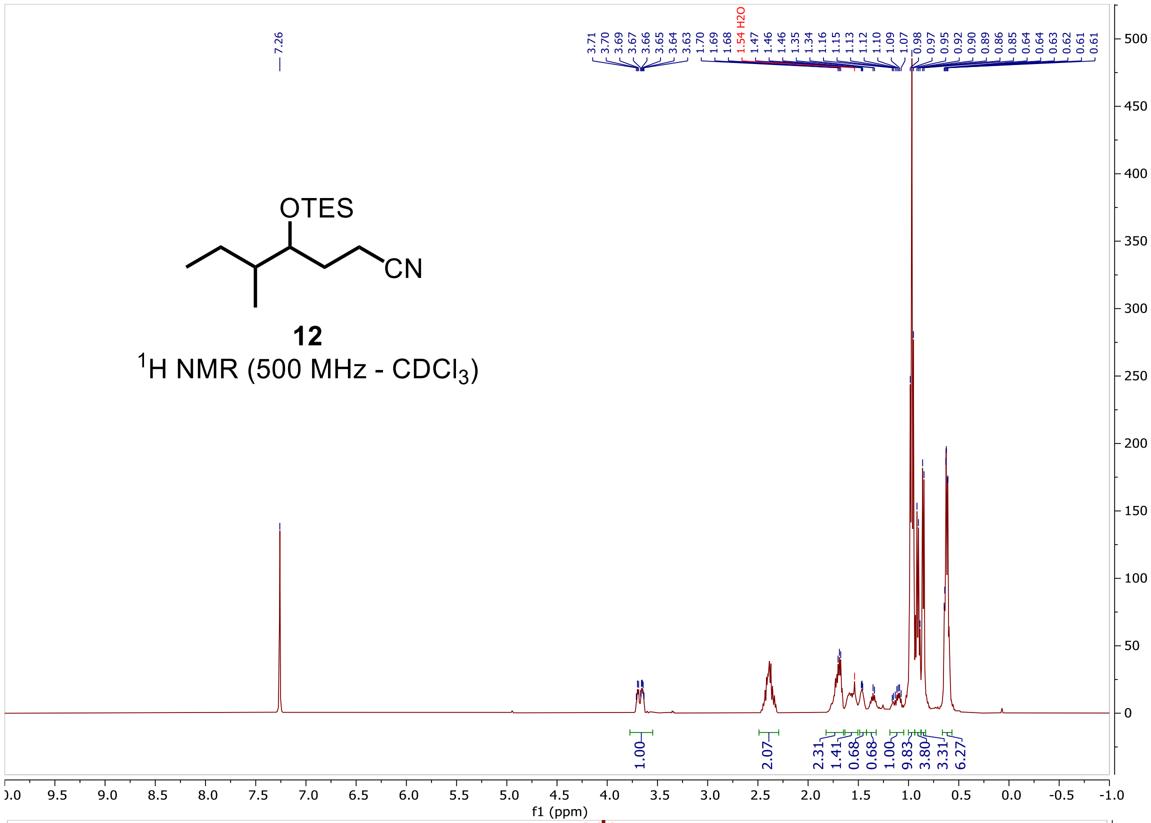
OTES
CN
12
^{1}H NMR (500 MHz - $CDCl_3$)
7.26
3.71 3.70 3.69 3.67 3.66 3.65 3.64 3.63
1.70 1.69 1.68 1.54 H2O 1.47 1.46 1.46 1.35 1.34 1.16 1.15 1.13 1.12 1.10 1.09 1.07 0.98 0.97 0.95 0.92 0.90 0.89 0.86 0.85 0.64 0.64 0.63 0.62 0.61 0.61
1.00 2.07 2.31 1.41 0.68 0.68 1.00 9.83 3.80 3.31 6.27
f1 (ppm)

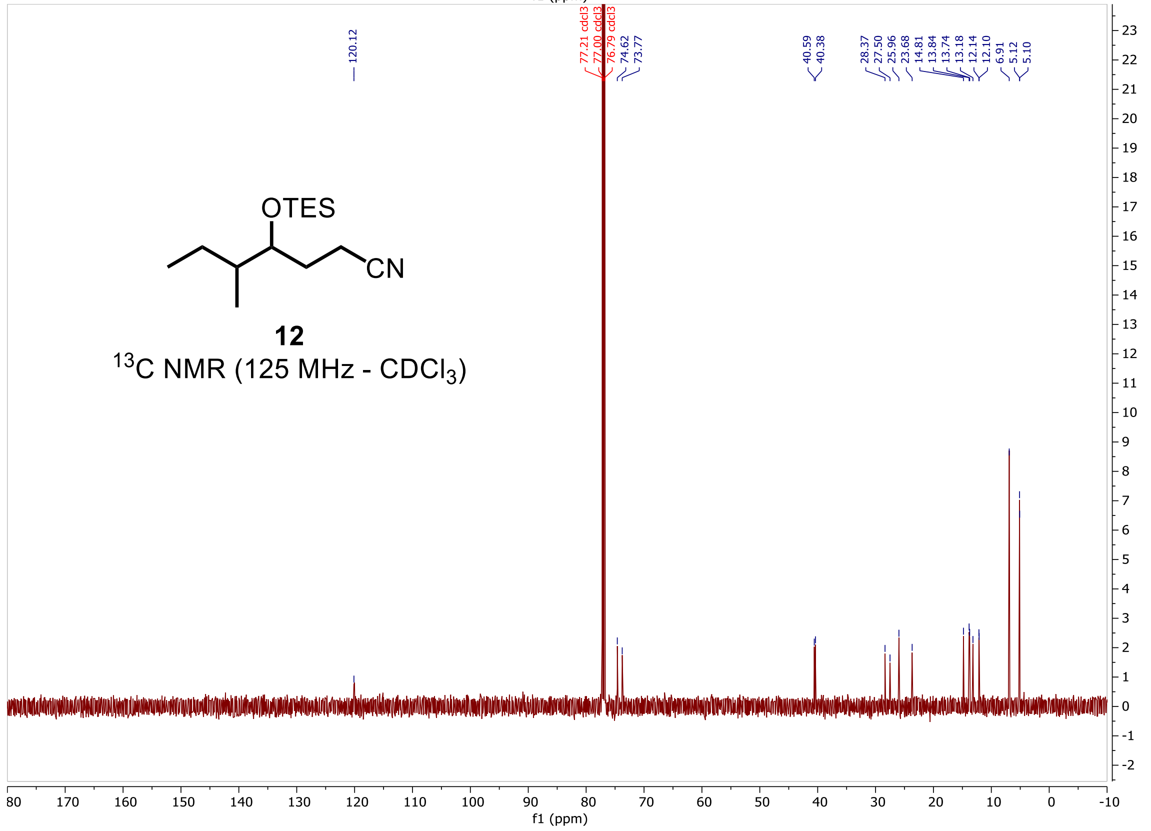
OTES
CN
12
^{13}C NMR (125 MHz - $CDCl_3$)
120.12
77.21 cdcl3 77.00 cdcl3 76.79 cdcl3 74.62 73.77
40.59 40.38
28.37 27.50 25.96 23.68 14.81 13.84 13.74 13.18 12.14 12.10 6.91 5.12 5.10
f1 (ppm)

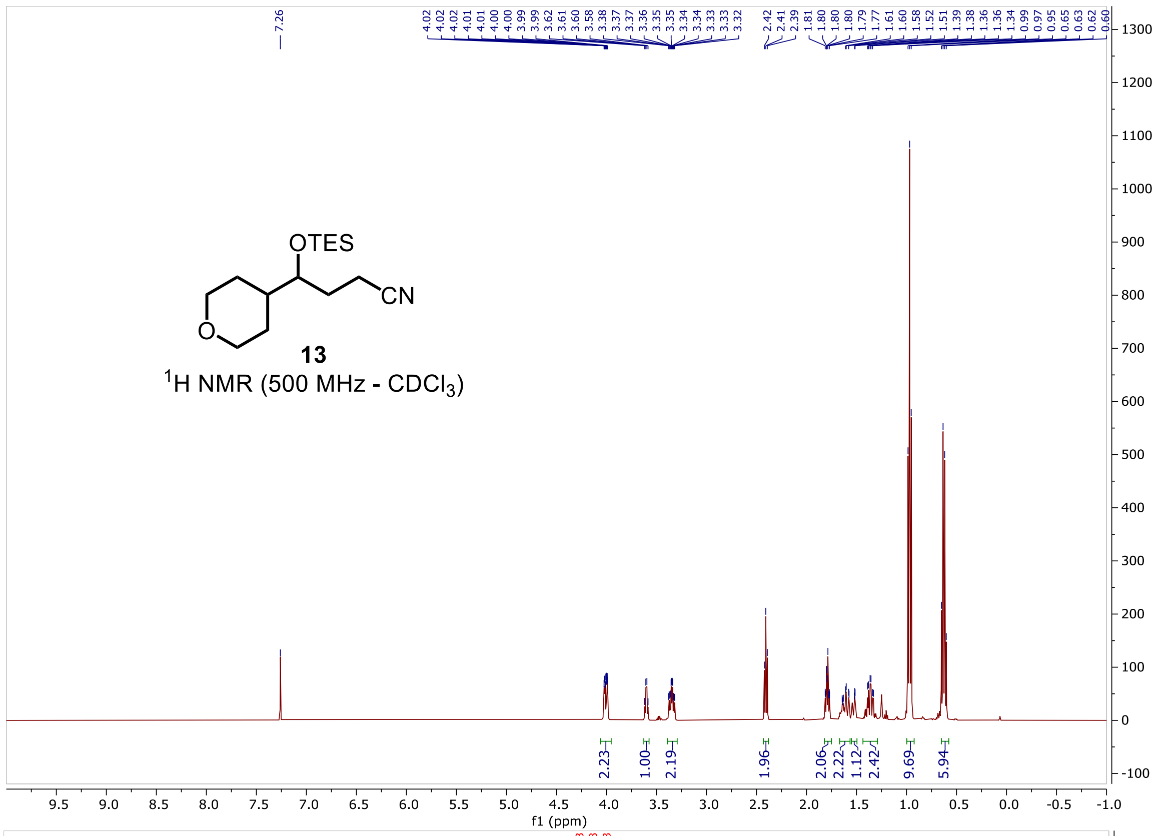
OTES
CN
O
13
^{1}H NMR (500 MHz - CDCl$_3$)
f1 (ppm)

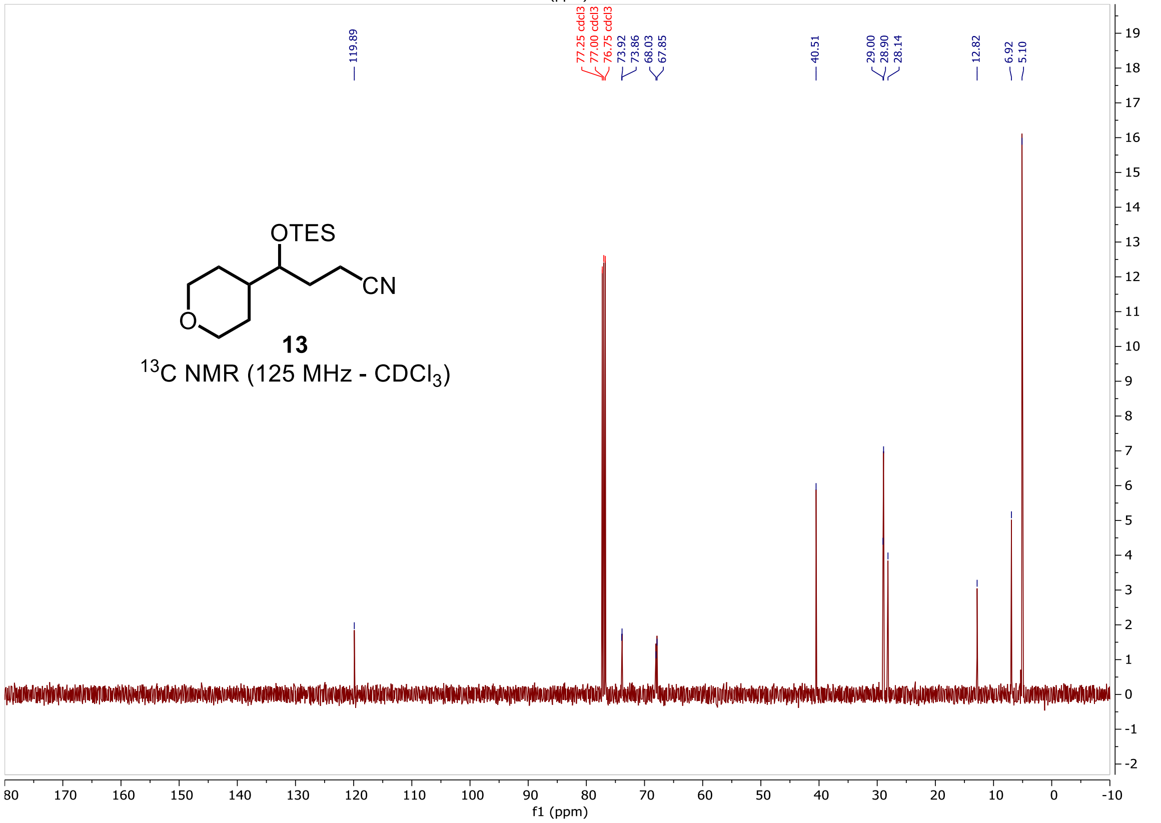
OTES
CN
O
13
^{13}C NMR (125 MHz - CDCl$_3$)
f1 (ppm)

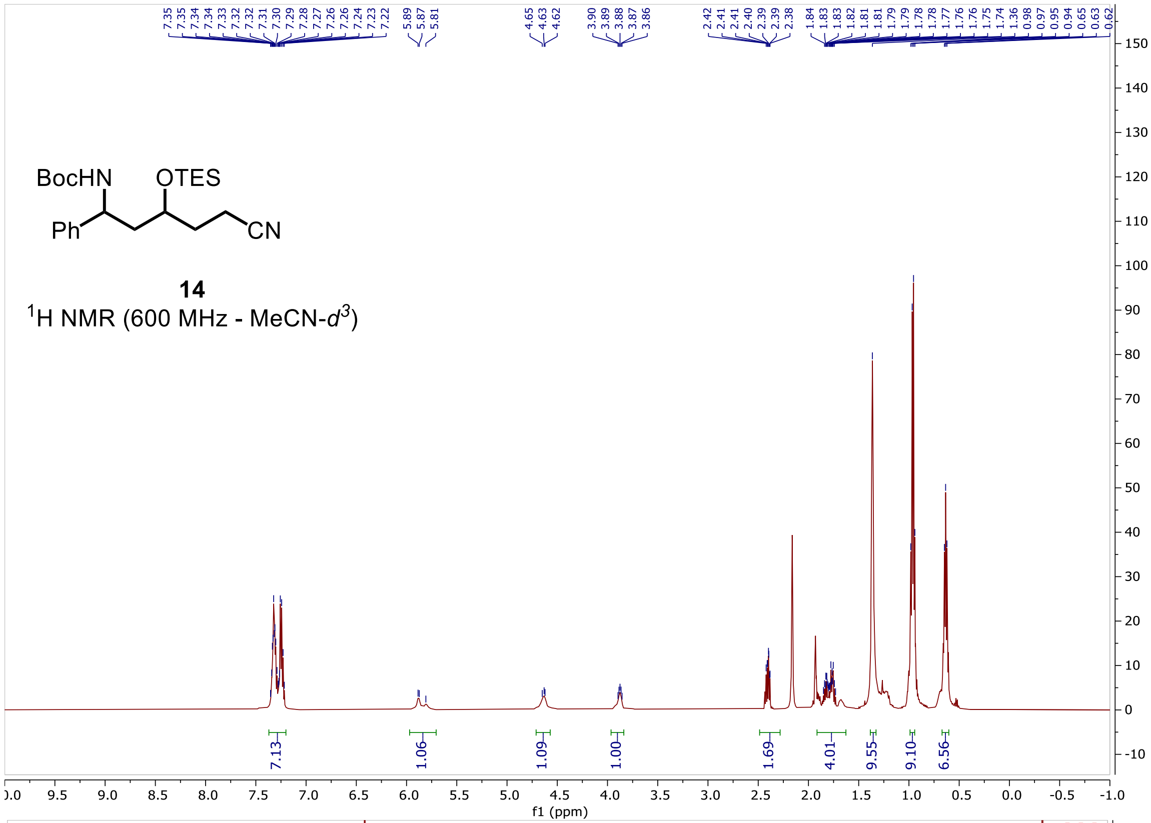
BocHN
OTES
Ph
CN
14
^{1}H NMR (600 MHz - MeCN-d^3)
f1 (ppm)

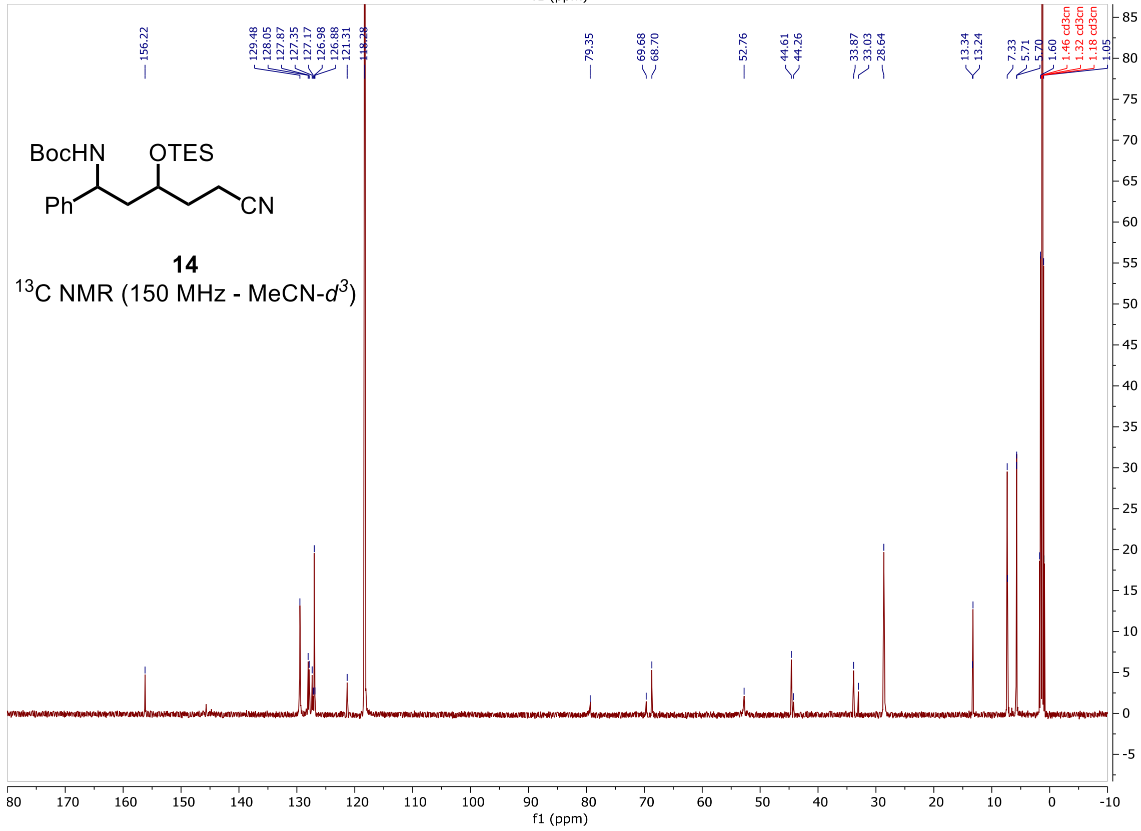
BocHN
OTES
Ph
CN
14
^{13}C NMR (150 MHz - MeCN-d^3)
f1 (ppm)

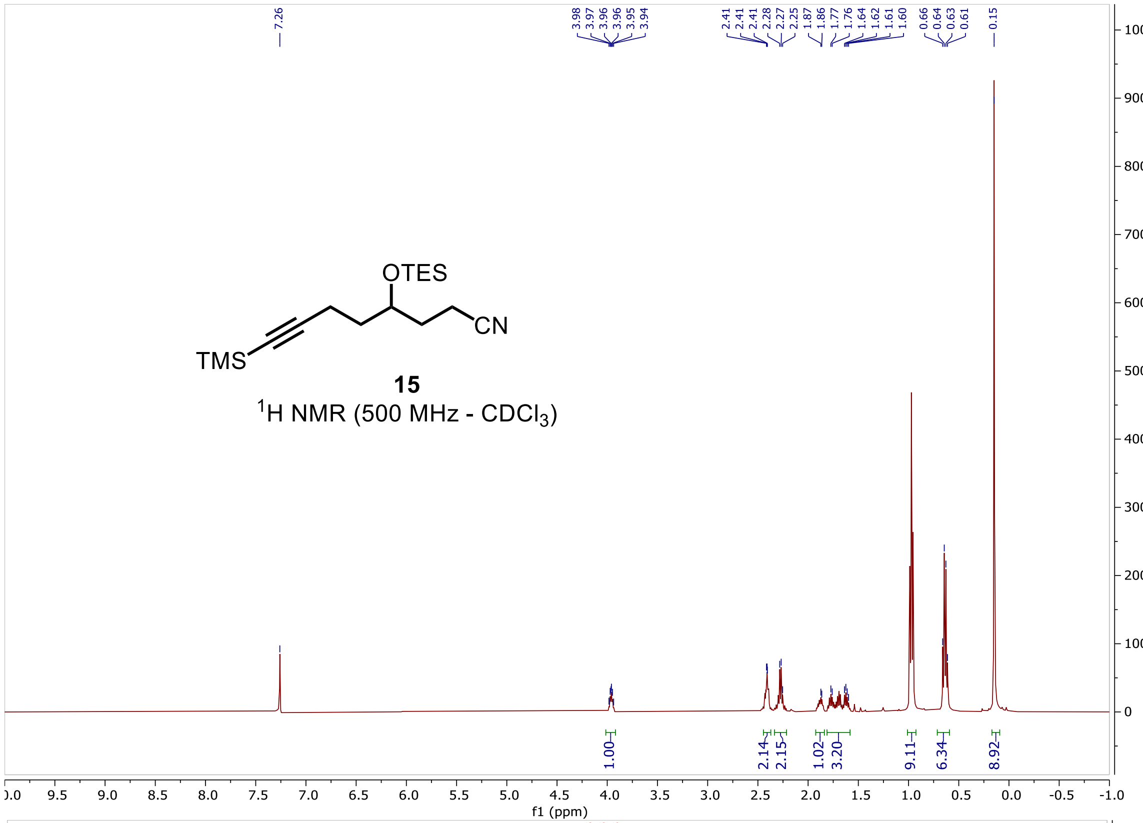

7.26
3.98 3.97 3.96 3.96 3.95 3.94
2.41 2.41 2.41 2.28 2.27 2.25 1.87 1.86 1.77 1.76 1.64 1.62 1.61 1.60
0.66 0.64 0.63 0.61
0.15
OTES
TMS
CN
15
1H NMR (500 MHz - CDCl3)
1.00
2.14
2.15
1.02
3.20
9.11
6.34
8.92
f1 (ppm)

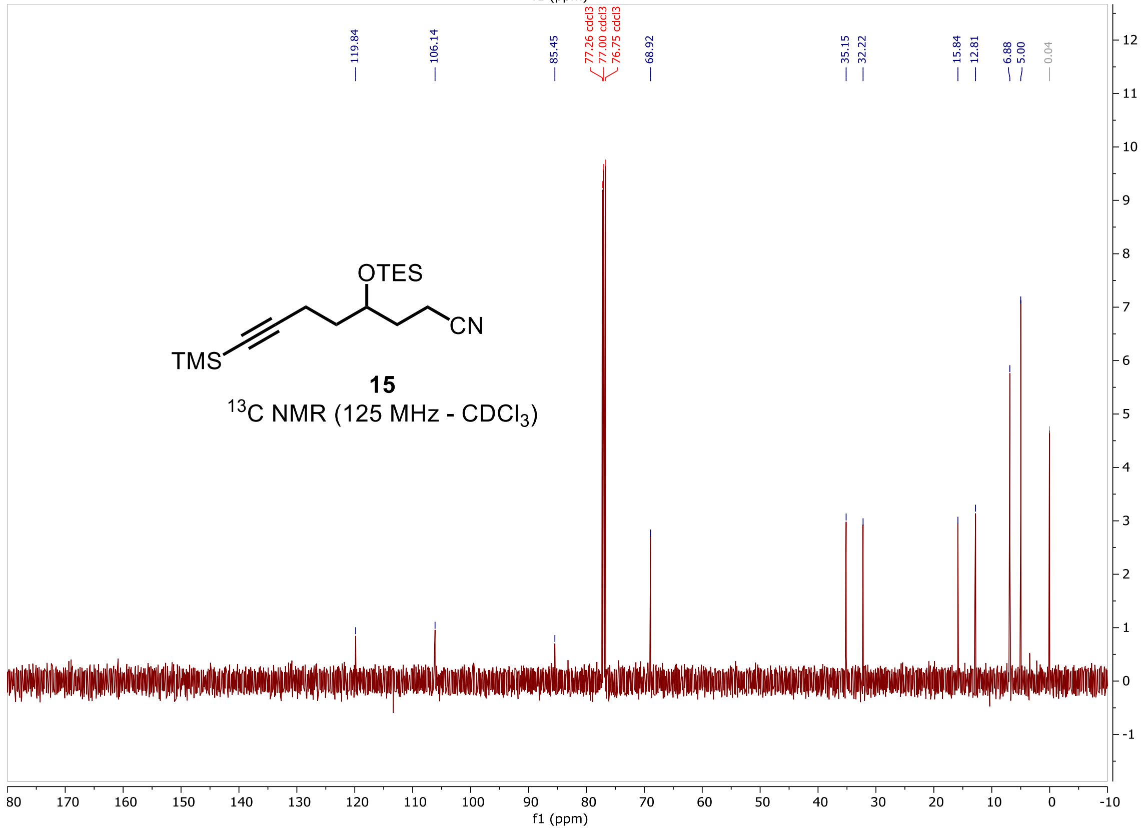

119.84
106.14
85.45
77.26 cdcl3
77.00 cdcl3
76.75 cdcl3
68.92
35.15
32.22
15.84
12.81
6.88
5.00
0.04
OTES
TMS
CN
15
13C NMR (125 MHz - CDCl3)
f1 (ppm)

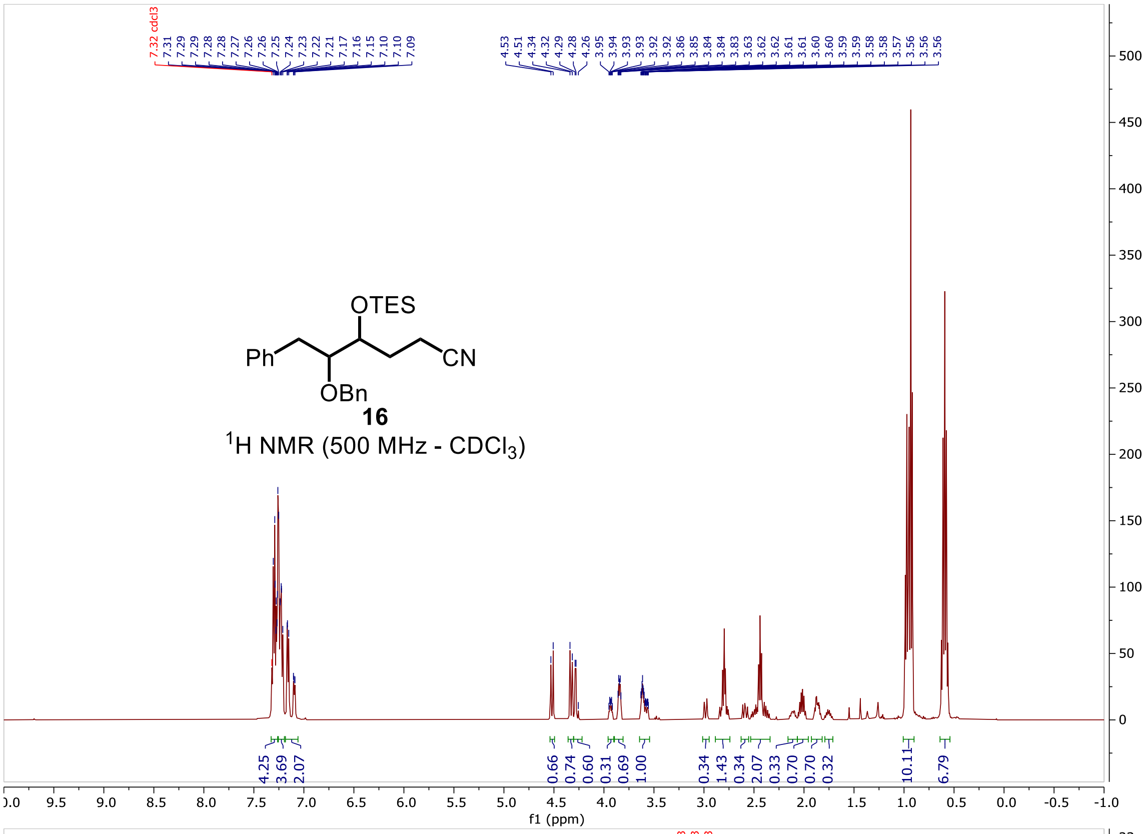
OTES
Ph
CN
OBn
16
^{1}H NMR (500 MHz - CDCl$_3$)
f1 (ppm)

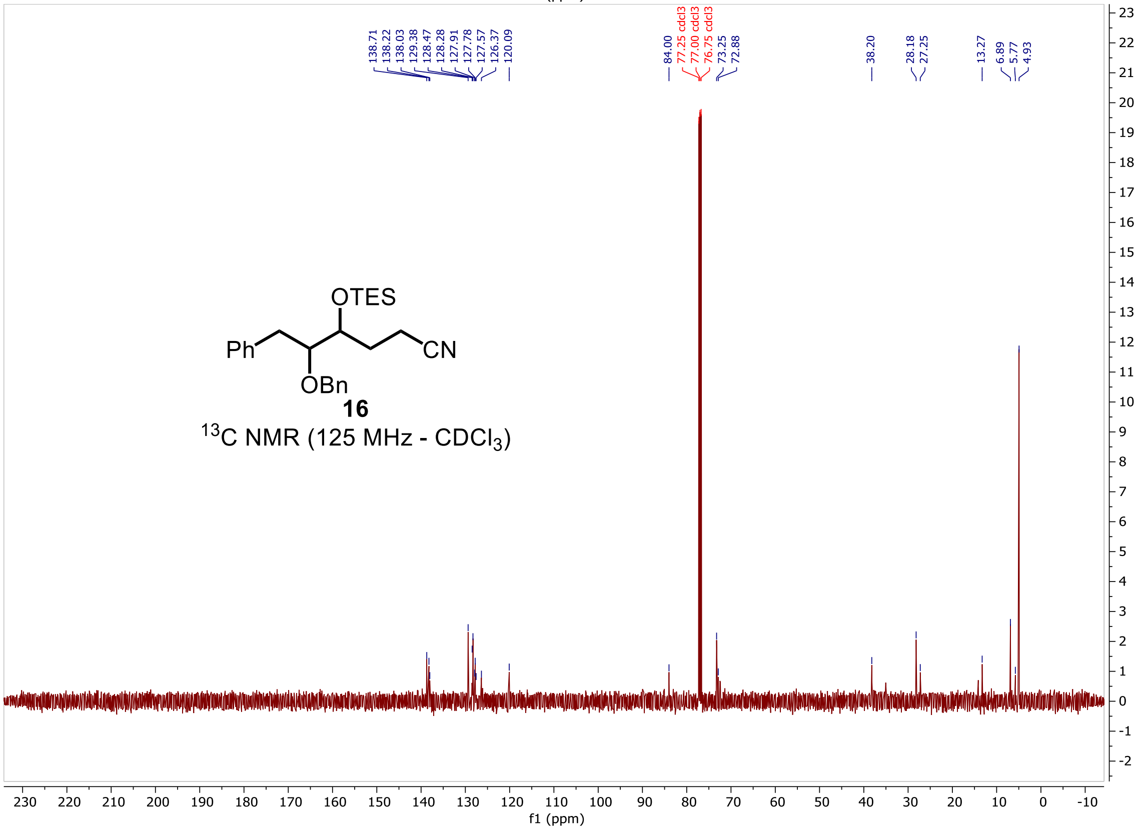
OTES
Ph
CN
OBn
16
^{13}C NMR (125 MHz - CDCl$_3$)
f1 (ppm)

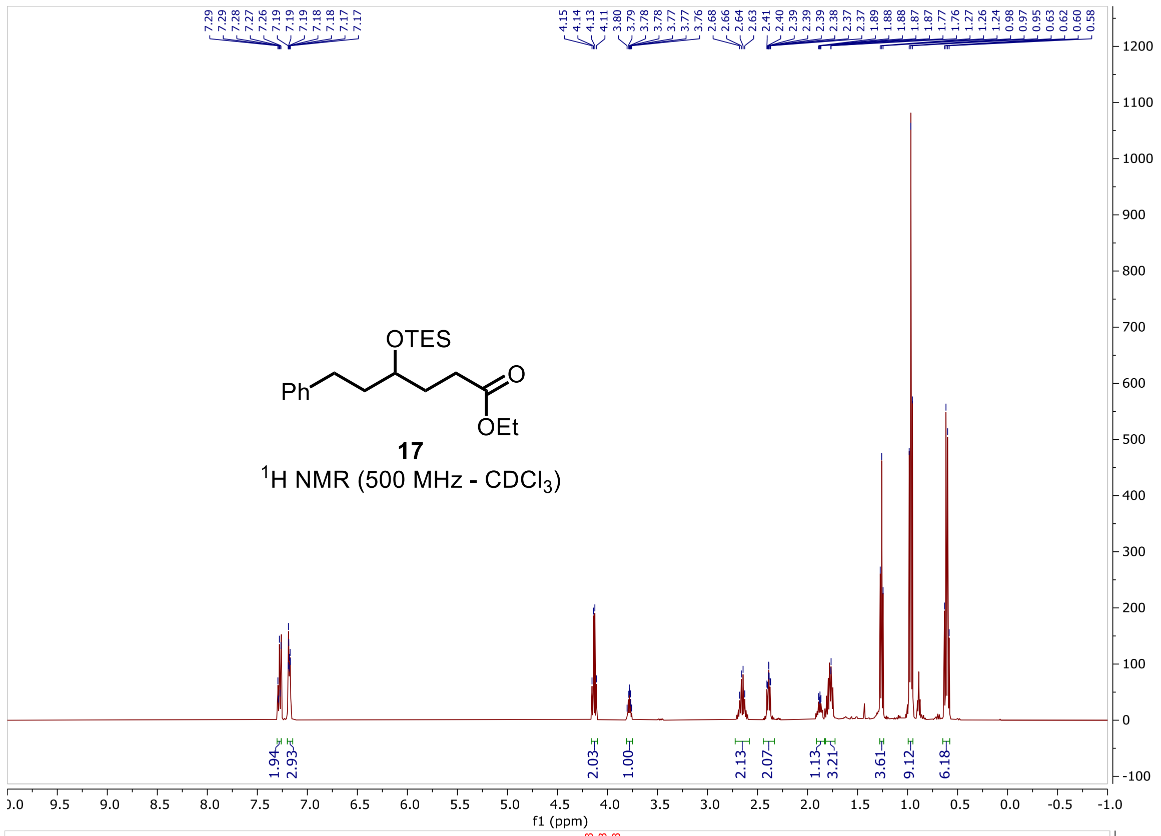
OTES
Ph
O
OEt
17
¹H NMR (500 MHz - CDCl₃)
f1 (ppm)

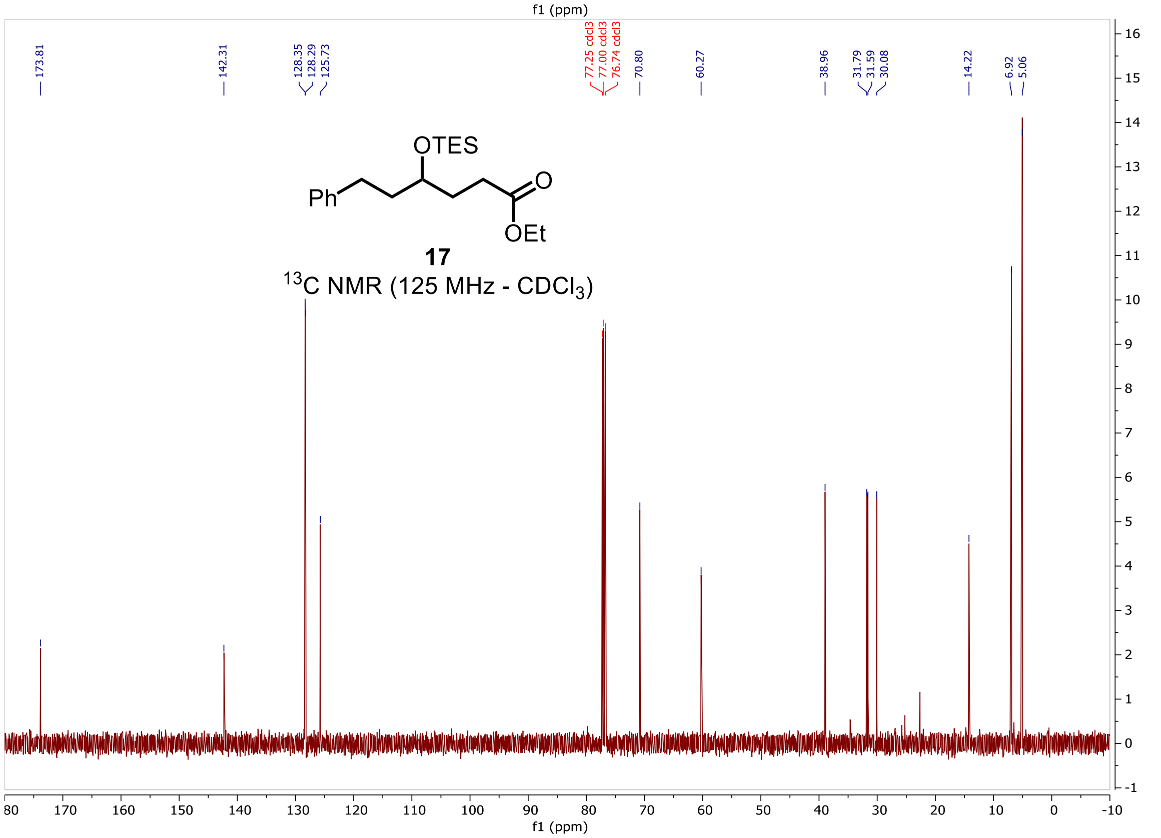
OTES
Ph
O
OEt
17
¹³C NMR (125 MHz - CDCl₃)
173.81
142.31
128.35
128.29
125.73
77.25 cdcl3
77.00 cdcl3
76.74 cdcl3
70.80
60.27
38.96
31.79
31.59
30.08
14.22
6.92
5.06
f1 (ppm)

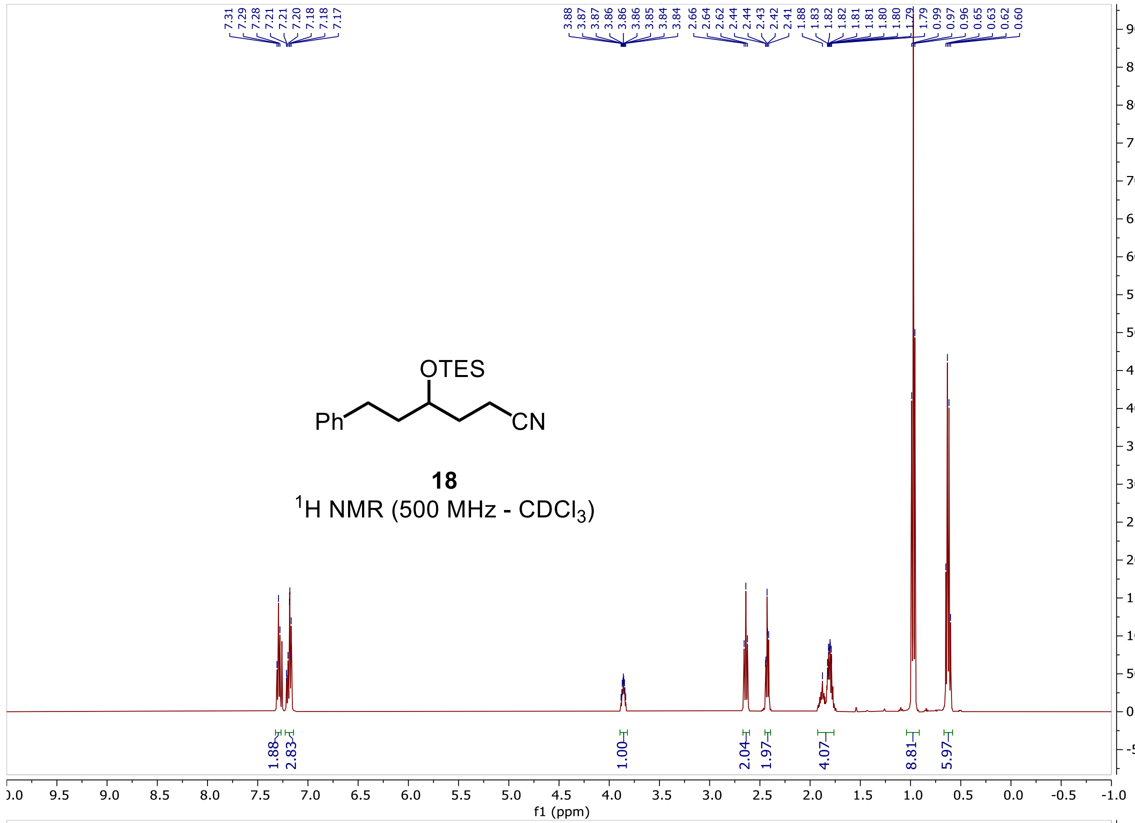
7.31 7.29 7.28 7.21 7.21 7.20 7.18 7.18 7.17
3.88 3.87 3.87 3.86 3.86 3.86 3.85 3.84 3.84
2.66 2.64 2.62 2.44 2.44 2.43 2.42 2.41
1.88 1.83 1.82 1.82 1.81 1.81 1.80 1.80 1.79 1.79
0.99 0.97 0.96 0.65 0.63 0.62 0.60
OTES
Ph
CN
18
1H NMR (500 MHz - CDCl3)
1.88 2.83 1.00 2.04 1.97 4.07 8.81 5.97
f1 (ppm)

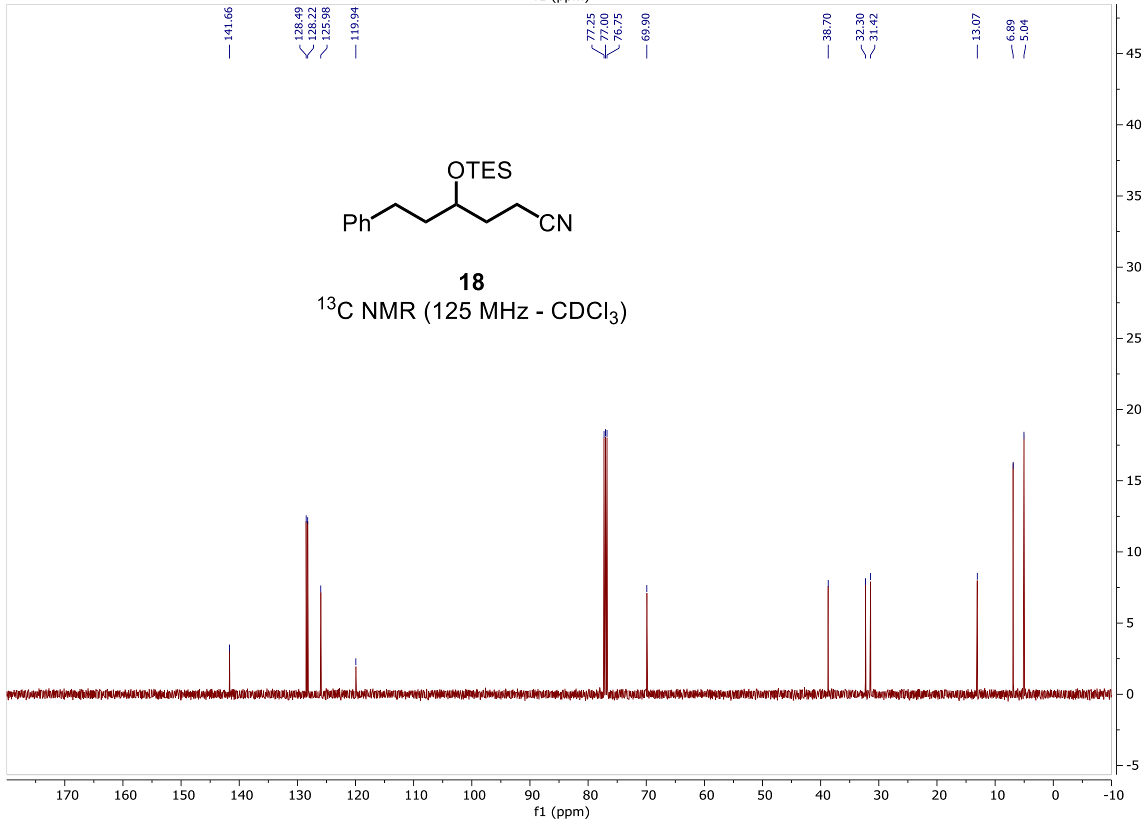
141.66
128.49 128.22 125.98
119.94
77.25 77.00 76.75
69.90
38.70
32.30 31.42
13.07
6.89 5.04
OTES
Ph
CN
18
13C NMR (125 MHz - CDCl3)
f1 (ppm)

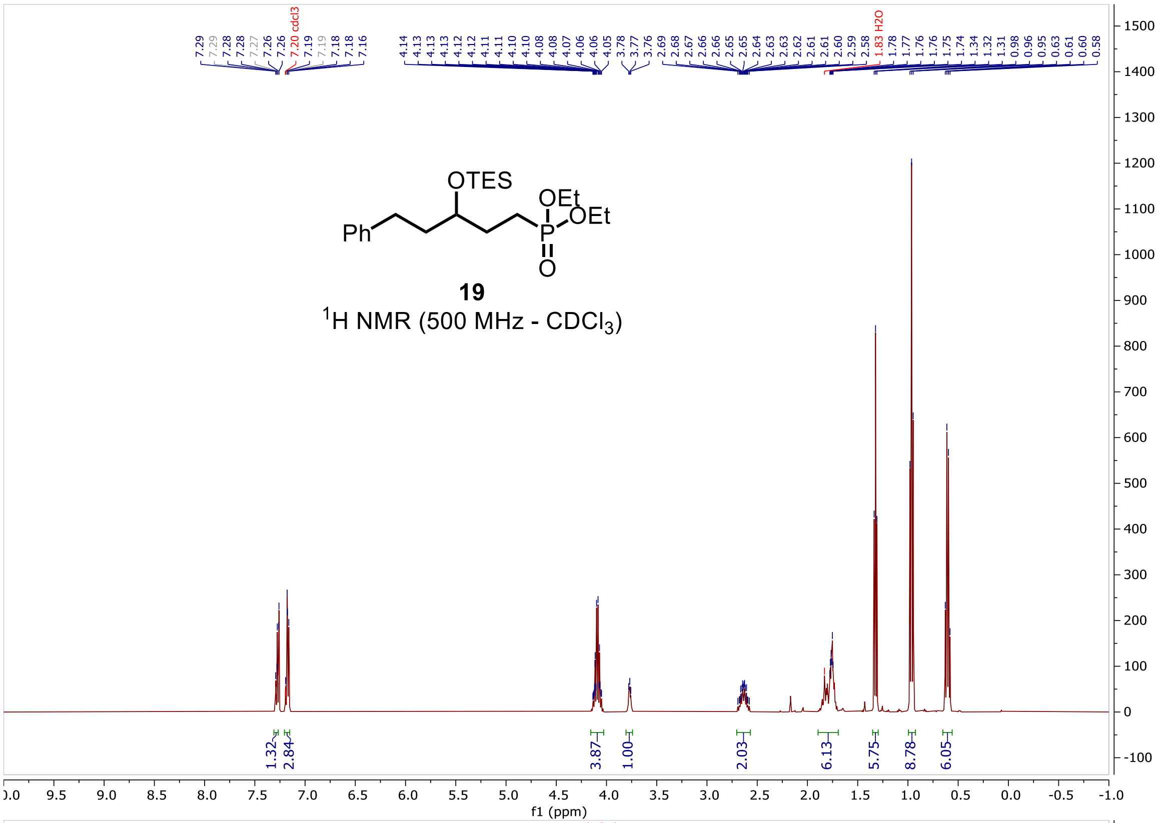
OTES
OEt
OEt
Ph
P
O
19
^{1}H NMR (500 MHz - CDCl$_3$)
f1 (ppm)

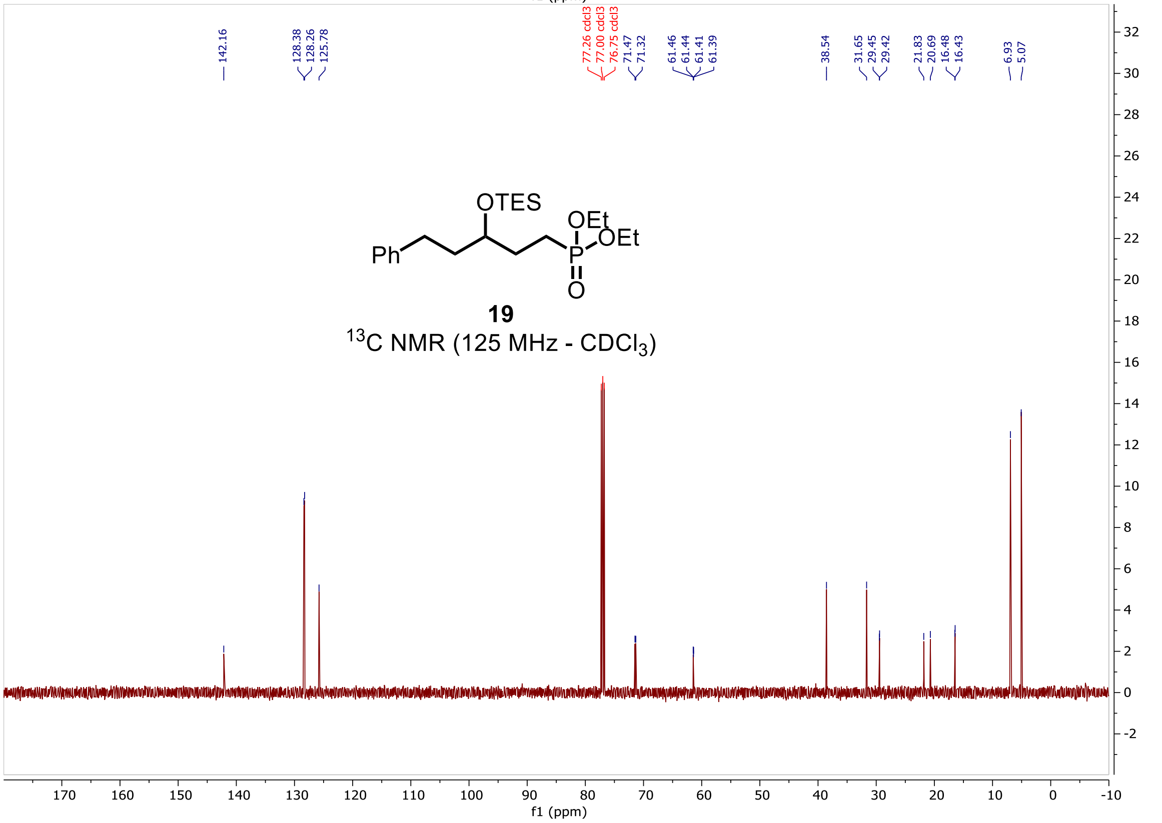
OTES
OEt
OEt
Ph
P
O
19
^{13}C NMR (125 MHz - CDCl$_3$)
f1 (ppm)

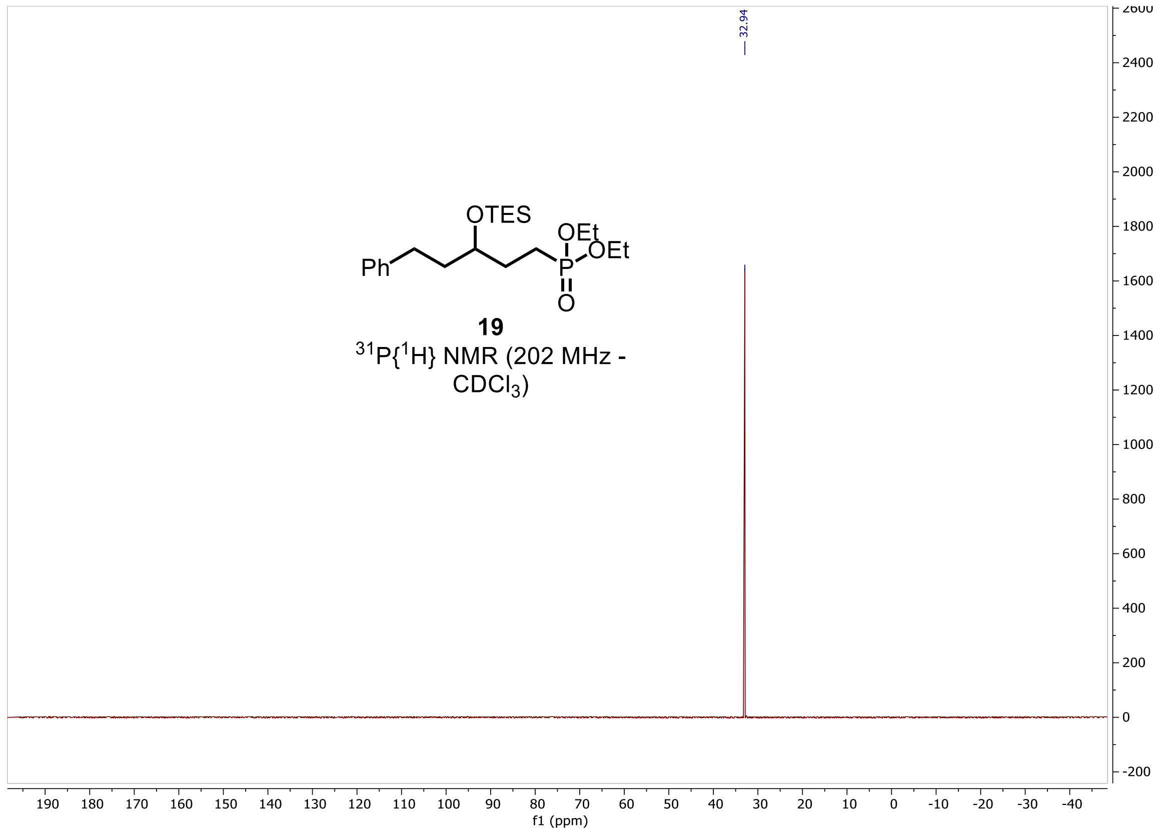

32.94
OTES
OEt
OEt
Ph
P
O
19
31P{1H} NMR (202 MHz - CDCl3)
2600
2400
2200
2000
1800
1600
1400
1200
1000
800
600
400
200
0
-200
190 180 170 160 150 140 130 120 110 100 90 80 70 60 50 40 30 20 10 0 -10 -20 -30 -40
f1 (ppm)

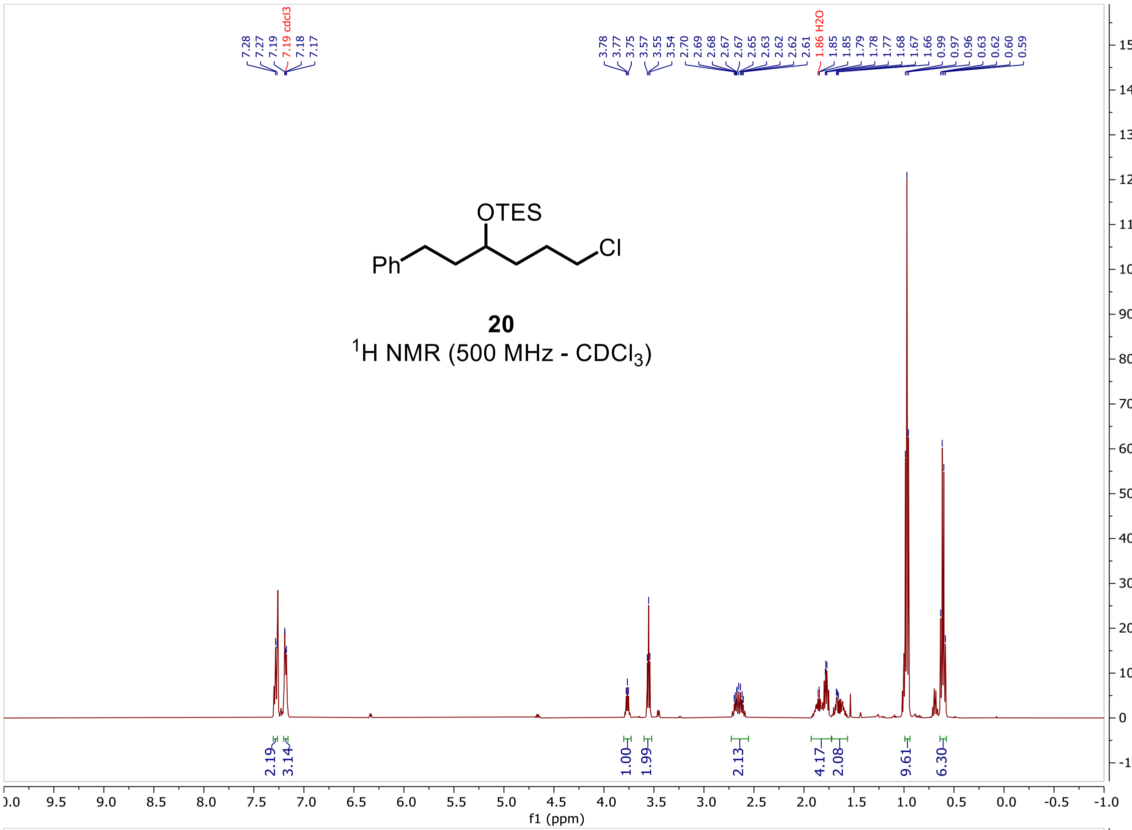
7.28
7.27
7.19
7.19 cdcl3
7.18
7.17
3.78
3.77
3.75
3.57
3.55
3.54
2.70
2.69
2.68
2.67
2.67
2.65
2.63
2.62
2.62
2.61
1.86 H2O
1.85
1.85
1.79
1.78
1.77
1.68
1.67
1.66
0.99
0.97
0.96
0.63
0.62
0.60
0.59
OTES
Ph
Cl
20
1H NMR (500 MHz - CDCl3)
2.19
3.14
1.00
1.99
2.13
4.17
2.08
9.61
6.30
f1 (ppm)

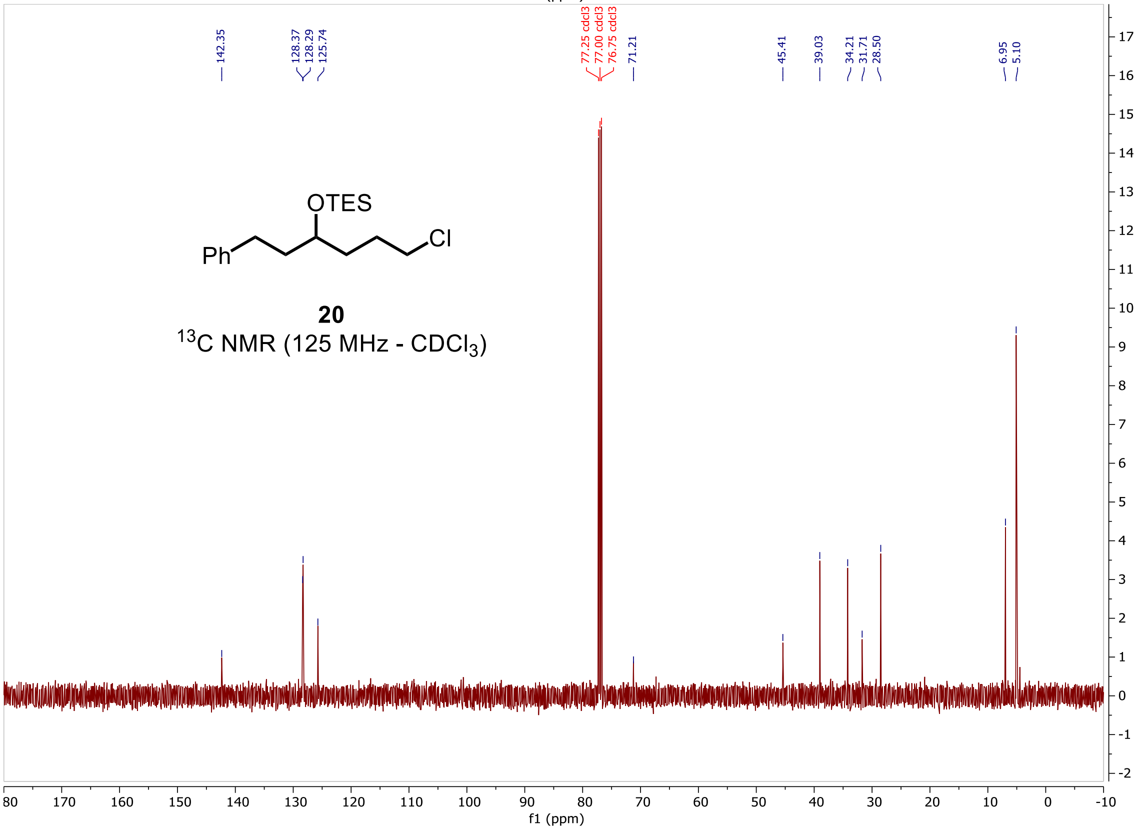
142.35
128.37
128.29
125.74
77.25 cdcl3
77.00 cdcl3
76.75 cdcl3
71.21
45.41
39.03
34.21
31.71
28.50
6.95
5.10
OTES
Ph
Cl
20
13C NMR (125 MHz - CDCl3)
f1 (ppm)

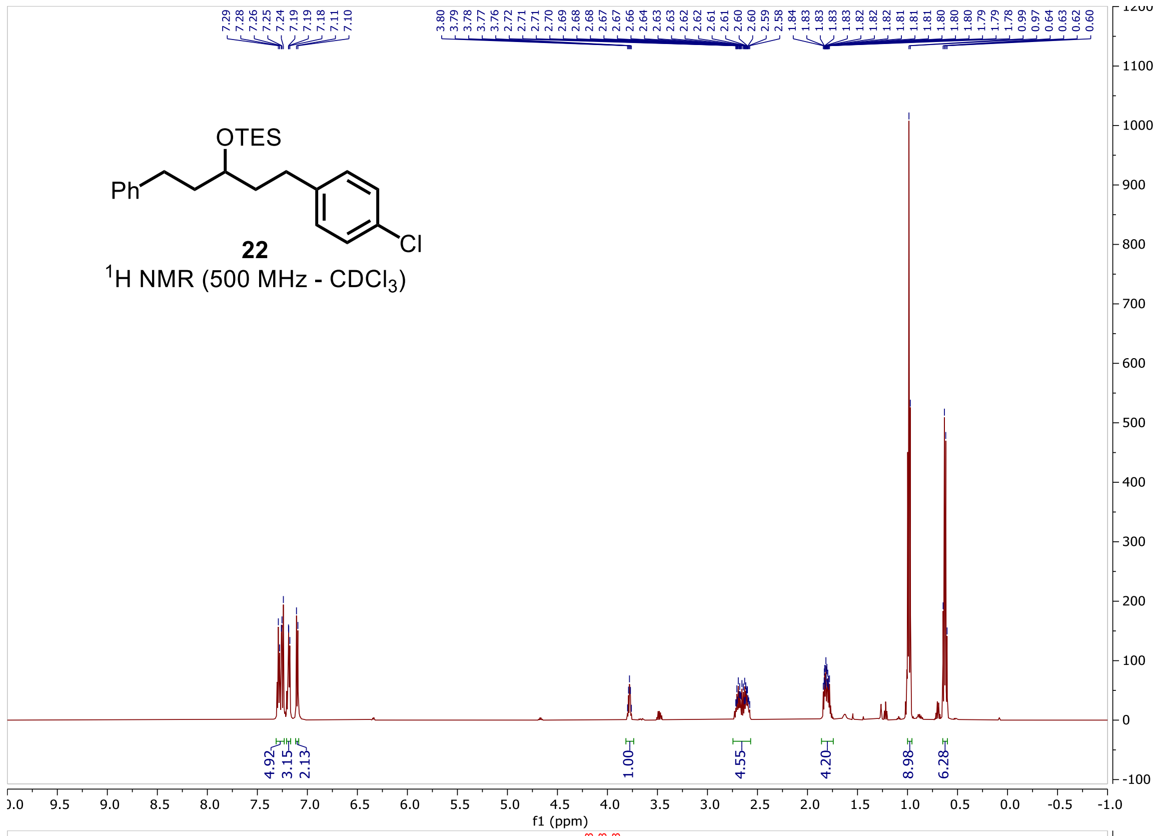
OTES
Ph
Cl
22
^{1}H NMR (500 MHz - CDCl$_3$)
f1 (ppm)

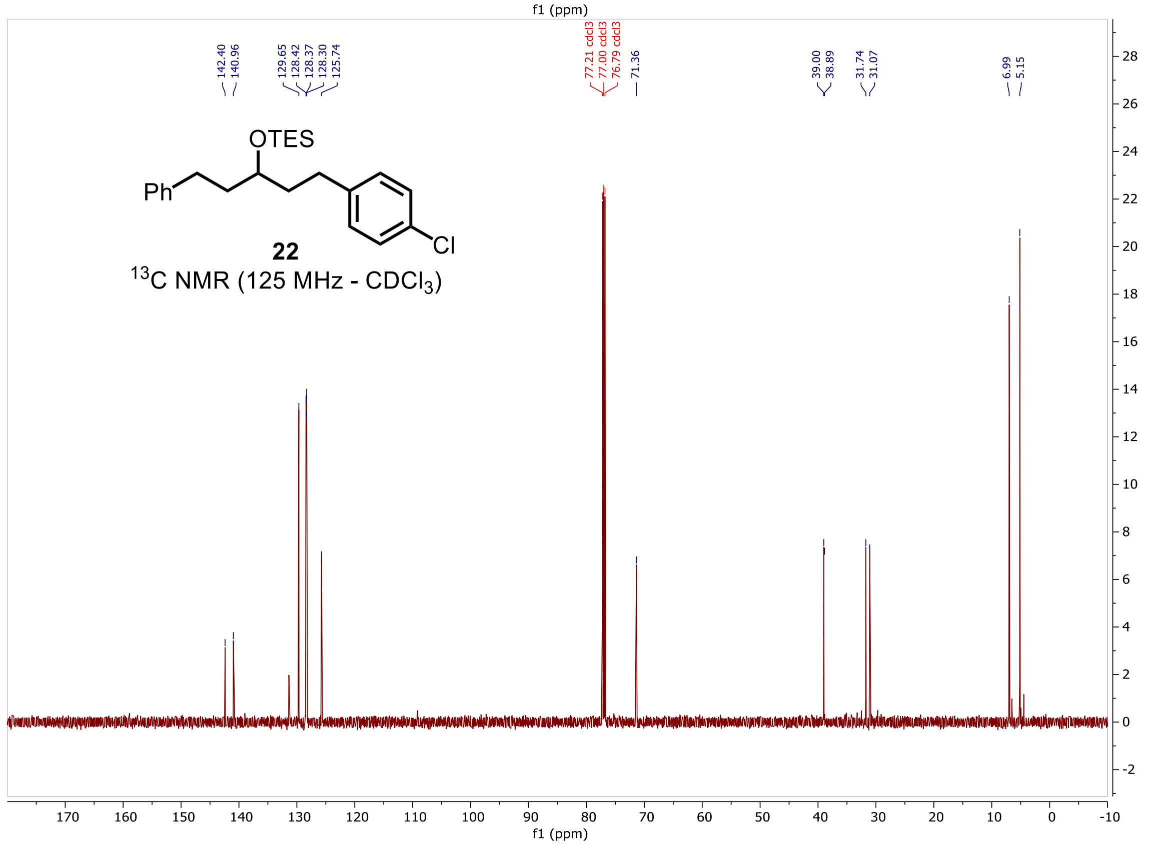
OTES
Ph
Cl
22
^{13}C NMR (125 MHz - CDCl$_3$)
f1 (ppm)

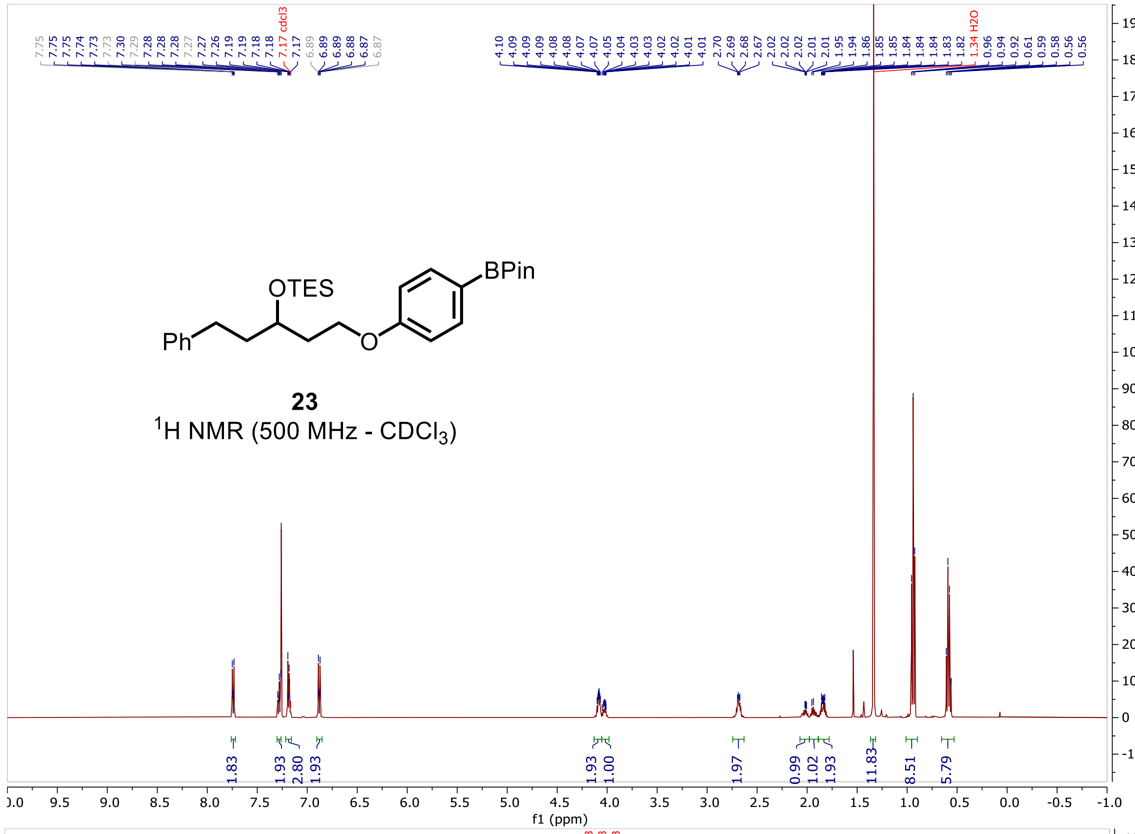
OTES
BPin
Ph
O
23
^{1}H NMR (500 MHz - $CDCl_3$)
f1 (ppm)

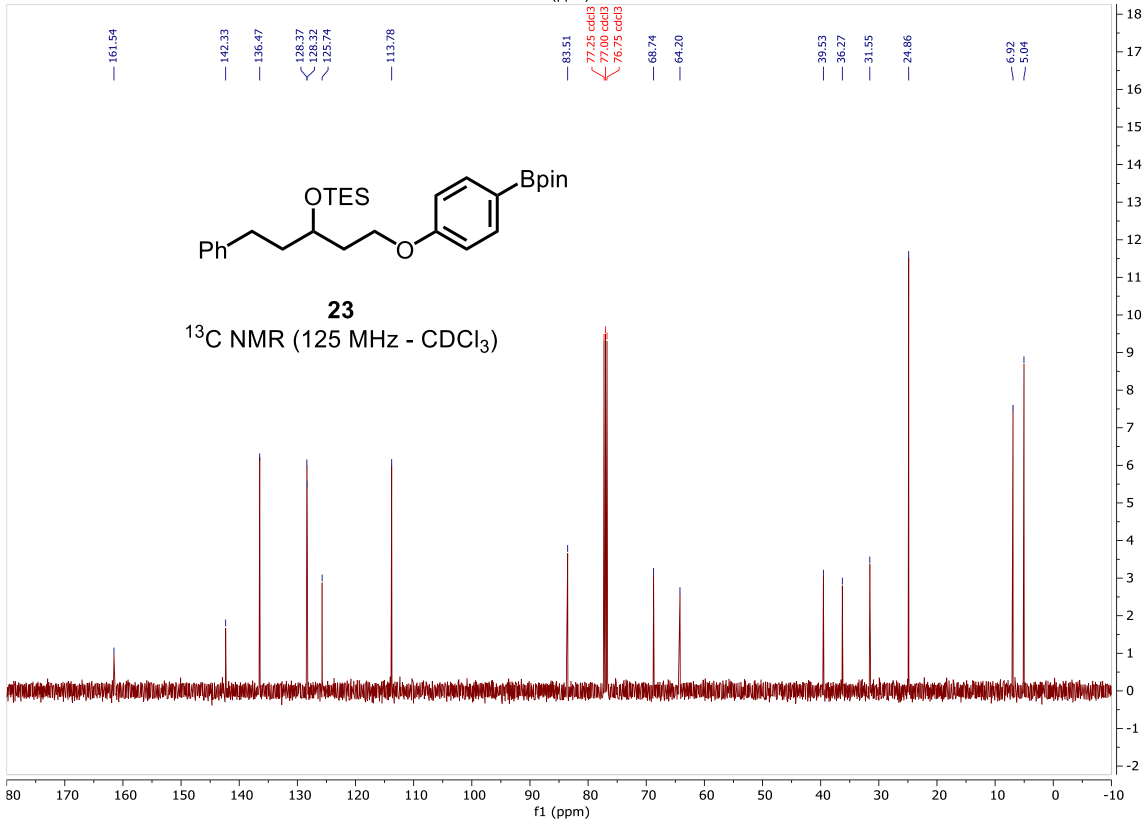
OTES
Bpin
Ph
O
23
^{13}C NMR (125 MHz - $CDCl_3$)
161.54
142.33
136.47
128.37
128.32
125.74
113.78
83.51
77.25 cdcl3
77.00 cdcl3
76.75 cdcl3
68.74
64.20
39.53
36.27
31.55
24.86
6.92
5.04
f1 (ppm)

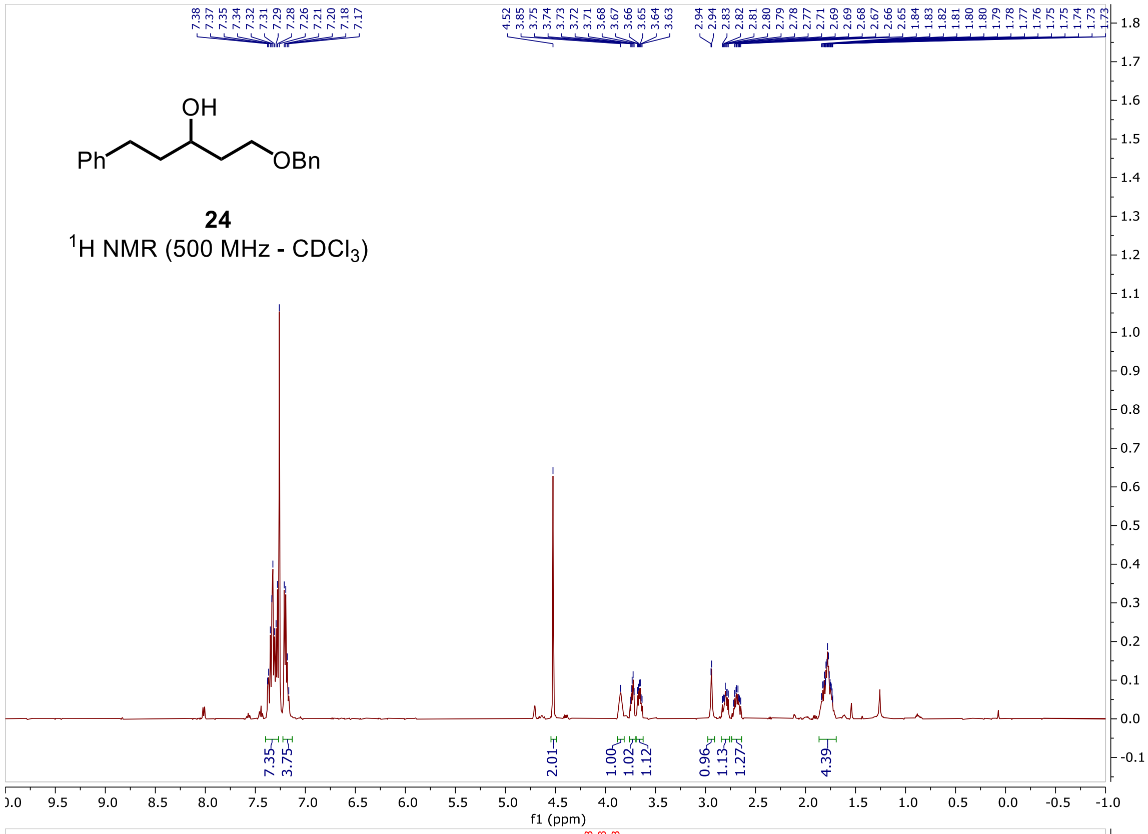
OH
Ph
OBn
24
1H NMR (500 MHz - CDCl3)
f1 (ppm)

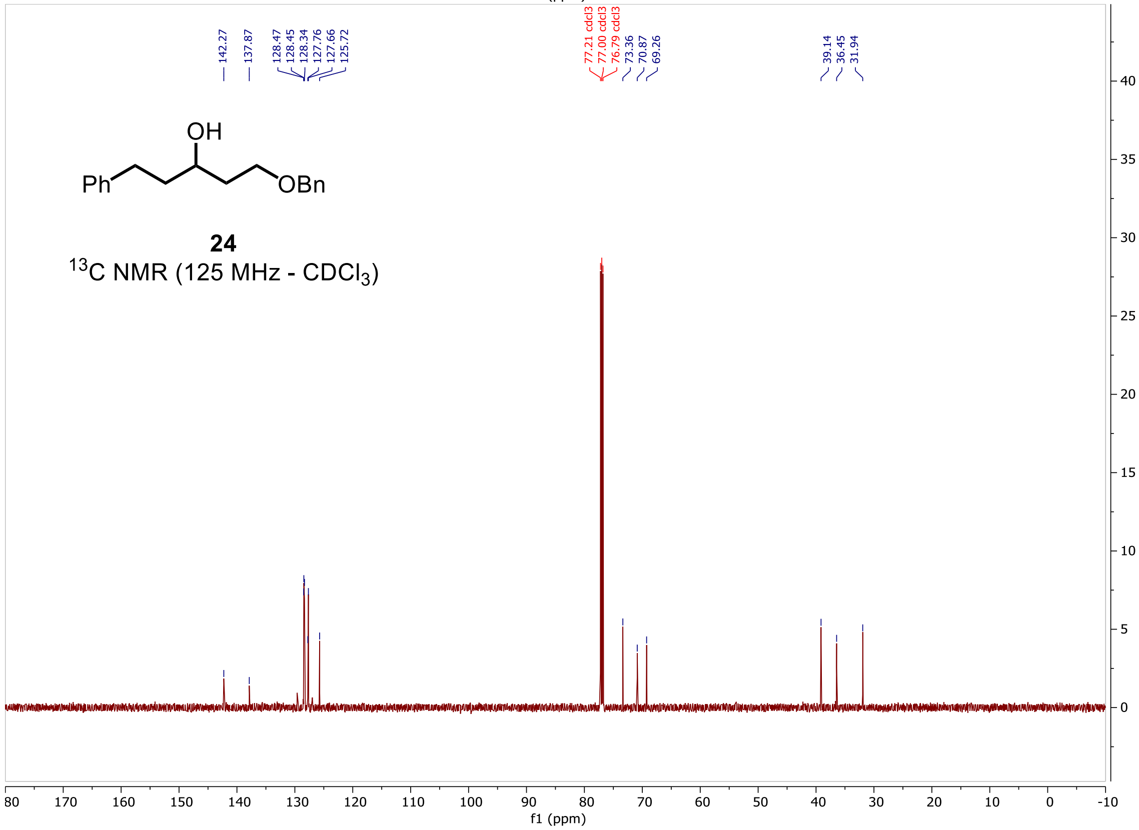
OH
Ph
OBn
24
13C NMR (125 MHz - CDCl3)
142.27
137.87
128.47
128.45
128.34
127.76
127.66
125.72
77.21 cdcl3
77.00 cdcl3
76.79 cdcl3
73.36
70.87
69.26
39.14
36.45
31.94
f1 (ppm)

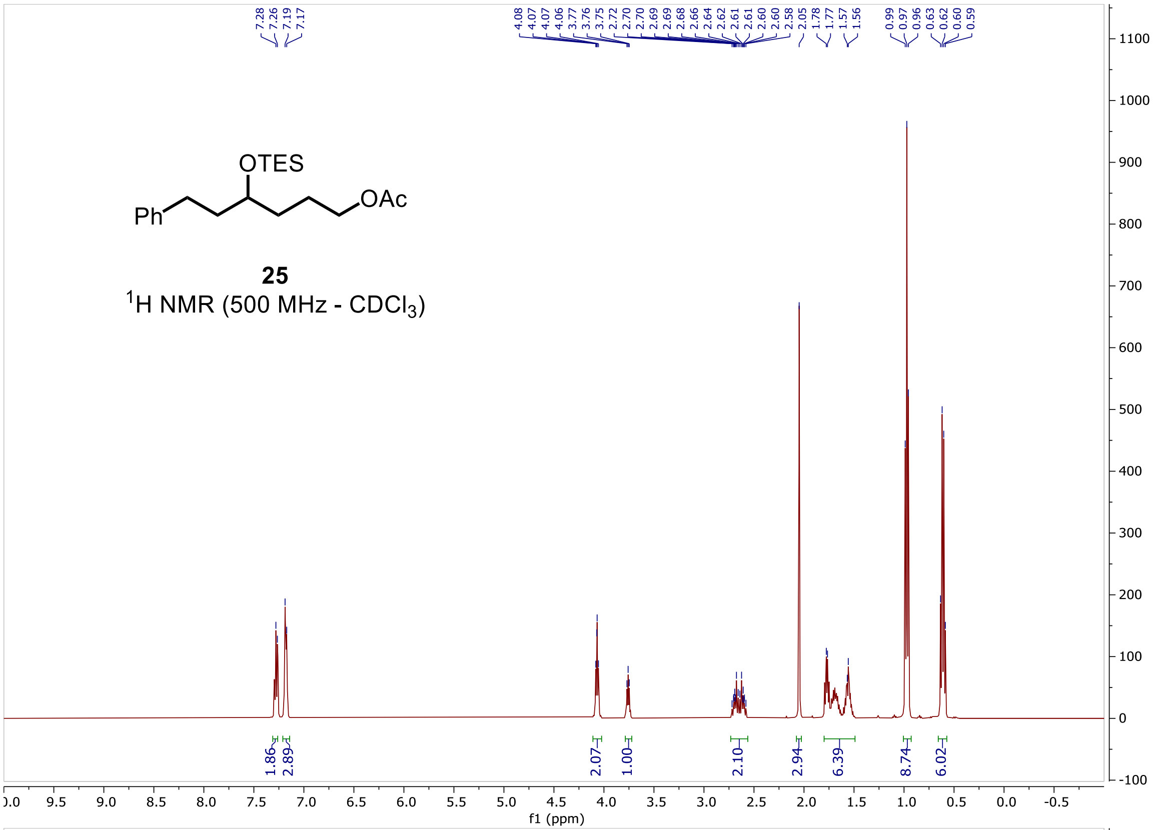
OTES
Ph
OAc
25
^{1}H NMR (500 MHz - CDCl$_3$)
7.28
7.26
7.19
7.17
4.08
4.07
4.07
4.06
3.77
3.76
3.75
2.72
2.70
2.70
2.69
2.69
2.68
2.66
2.64
2.62
2.61
2.61
2.60
2.60
2.58
2.05
1.78
1.77
1.57
1.56
0.99
0.97
0.96
0.63
0.62
0.60
0.59
1.86
2.89
2.07
1.00
2.10
2.94
6.39
8.74
6.02
f1 (ppm)

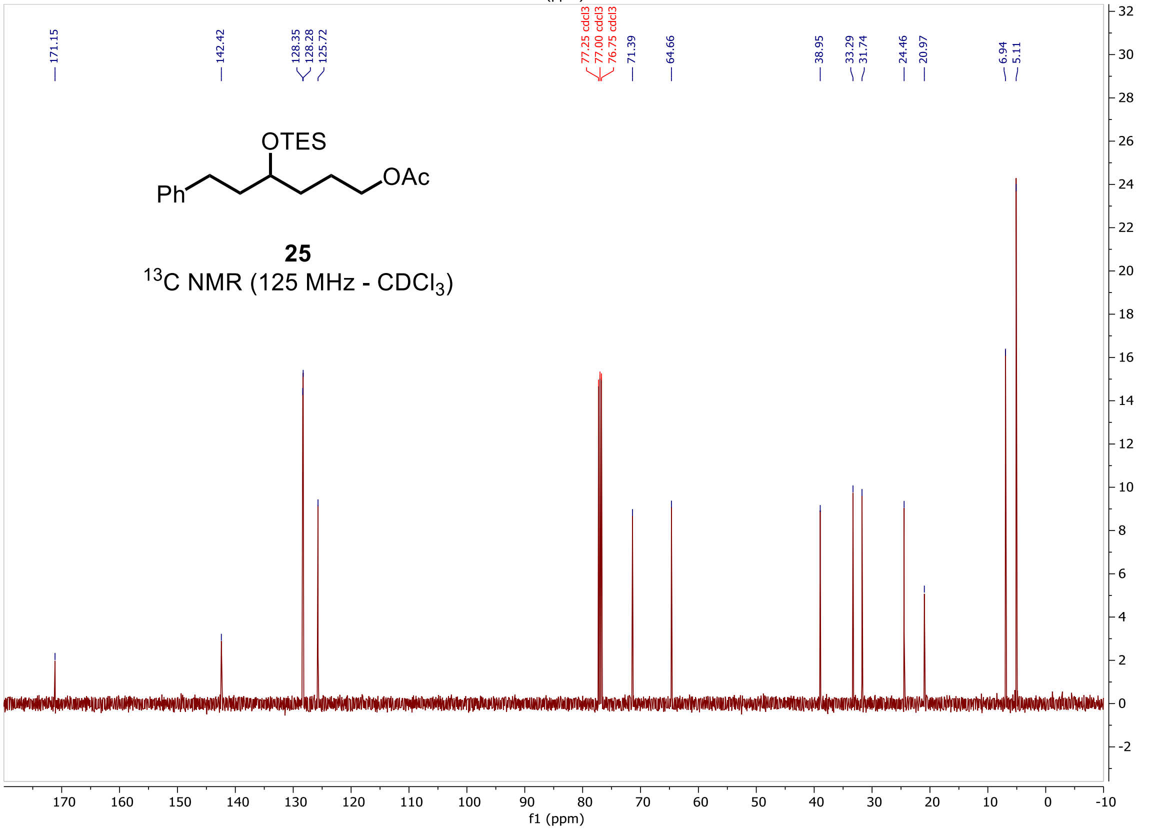
OTES
Ph
OAc
25
^{13}C NMR (125 MHz - CDCl$_3$)
171.15
142.42
128.35
128.28
125.72
77.25 cdcl3
77.00 cdcl3
76.75 cdcl3
71.39
64.66
38.95
33.29
31.74
24.46
20.97
6.94
5.11
f1 (ppm)

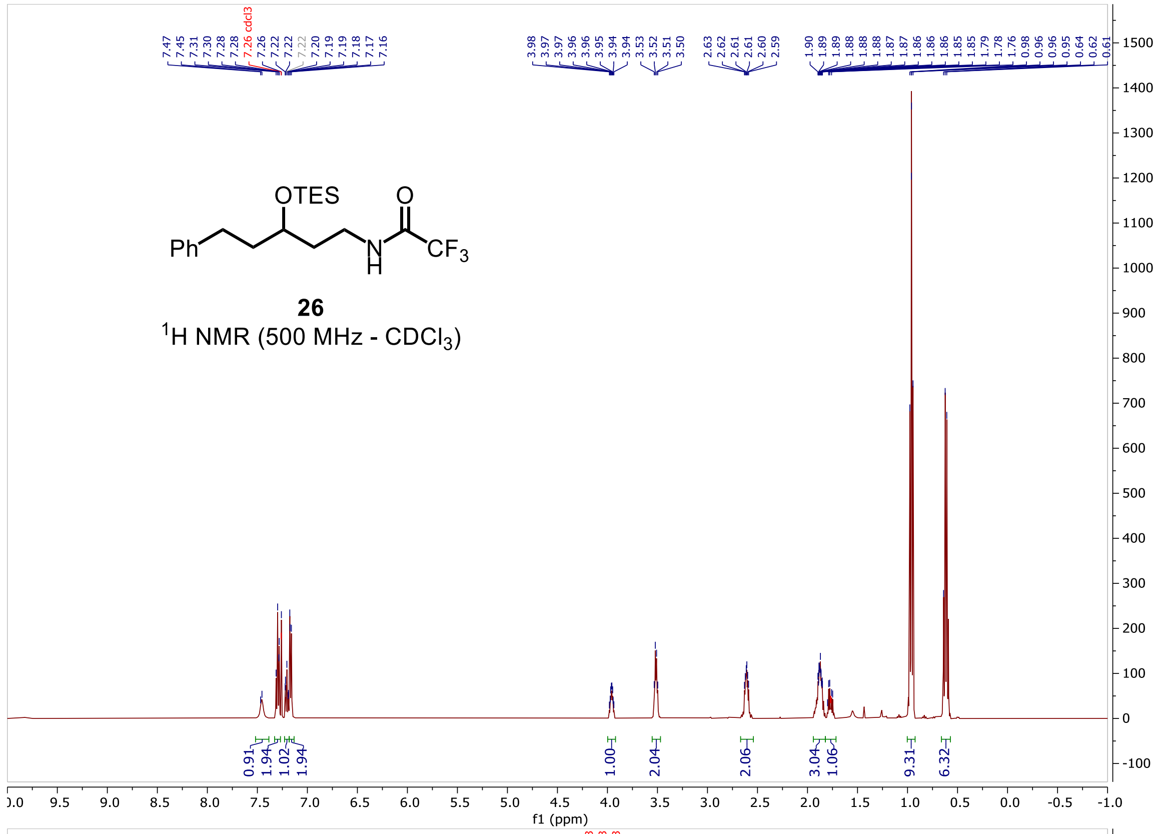
OTES
O
Ph
N
H
CF3
26
1H NMR (500 MHz - CDCl3)
f1 (ppm)

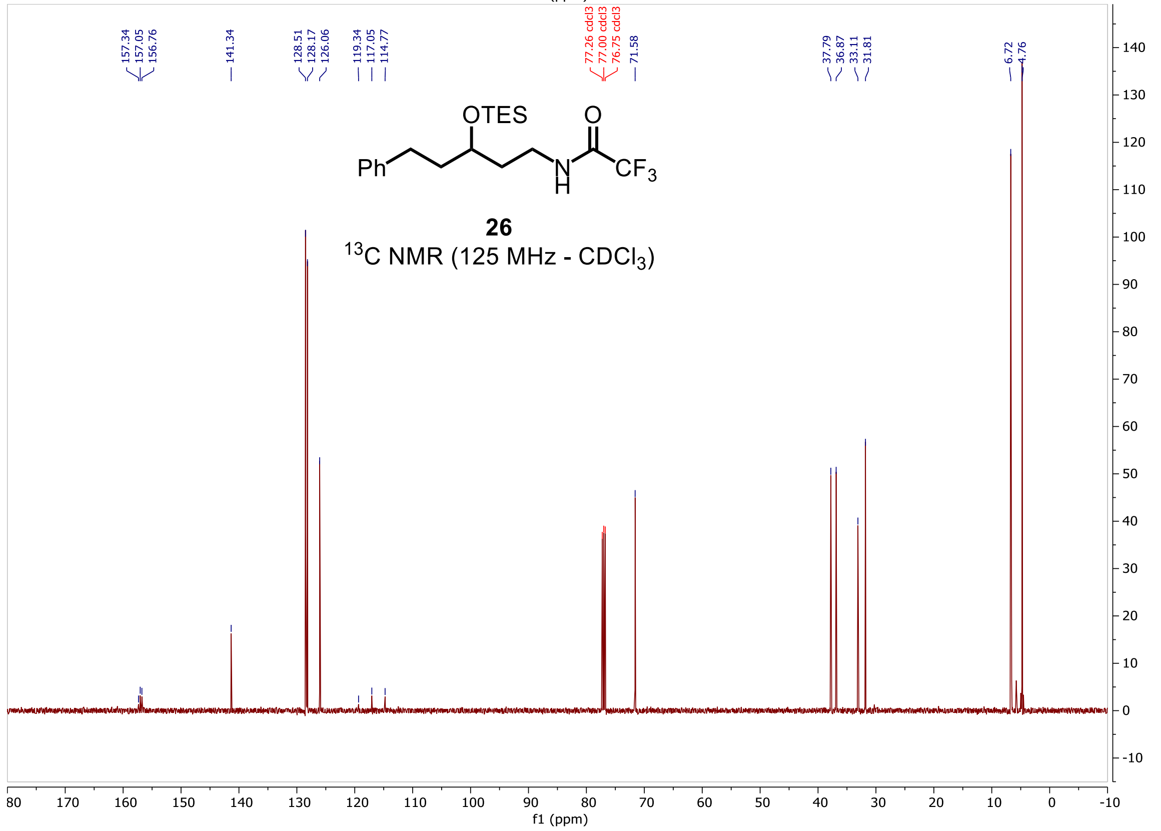
OTES
O
Ph
N
H
CF3
26
13C NMR (125 MHz - CDCl3)
f1 (ppm)

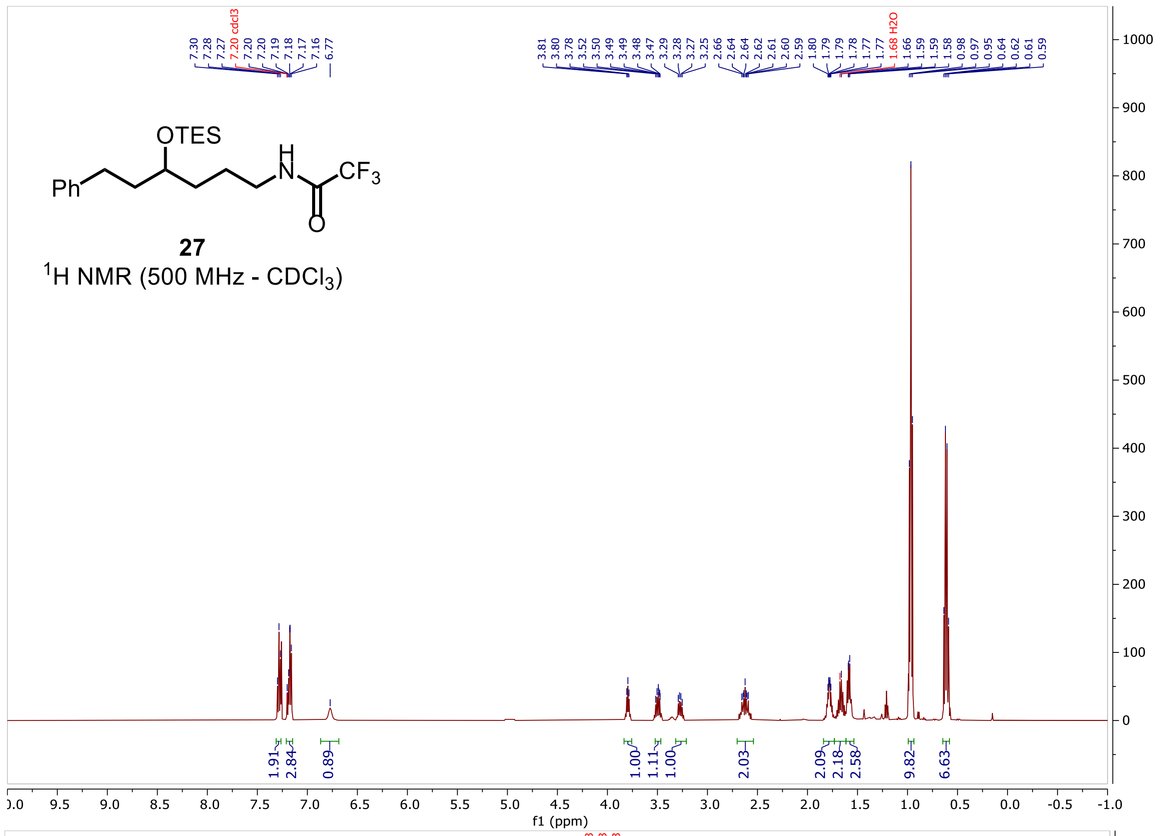
7.30 7.28 7.27 7.20 cdcl3 7.20 7.20 7.19 7.18 7.17 7.16 6.77
3.81 3.80 3.78 3.52 3.50 3.49 3.49 3.48 3.47 3.29 3.28 3.27 3.25 2.66 2.64 2.64 2.62 2.61 2.60 2.59 1.80 1.79 1.79 1.78 1.77 1.77 1.68 H2O 1.66 1.59 1.59 1.58 0.98 0.97 0.95 0.64 0.62 0.61 0.59
OTES
Ph
H
N
CF3
O
27
1H NMR (500 MHz - CDCl3)
1.91 2.84 0.89 1.00 1.11 1.00 2.03 2.09 2.18 2.58 9.82 6.63
f1 (ppm)

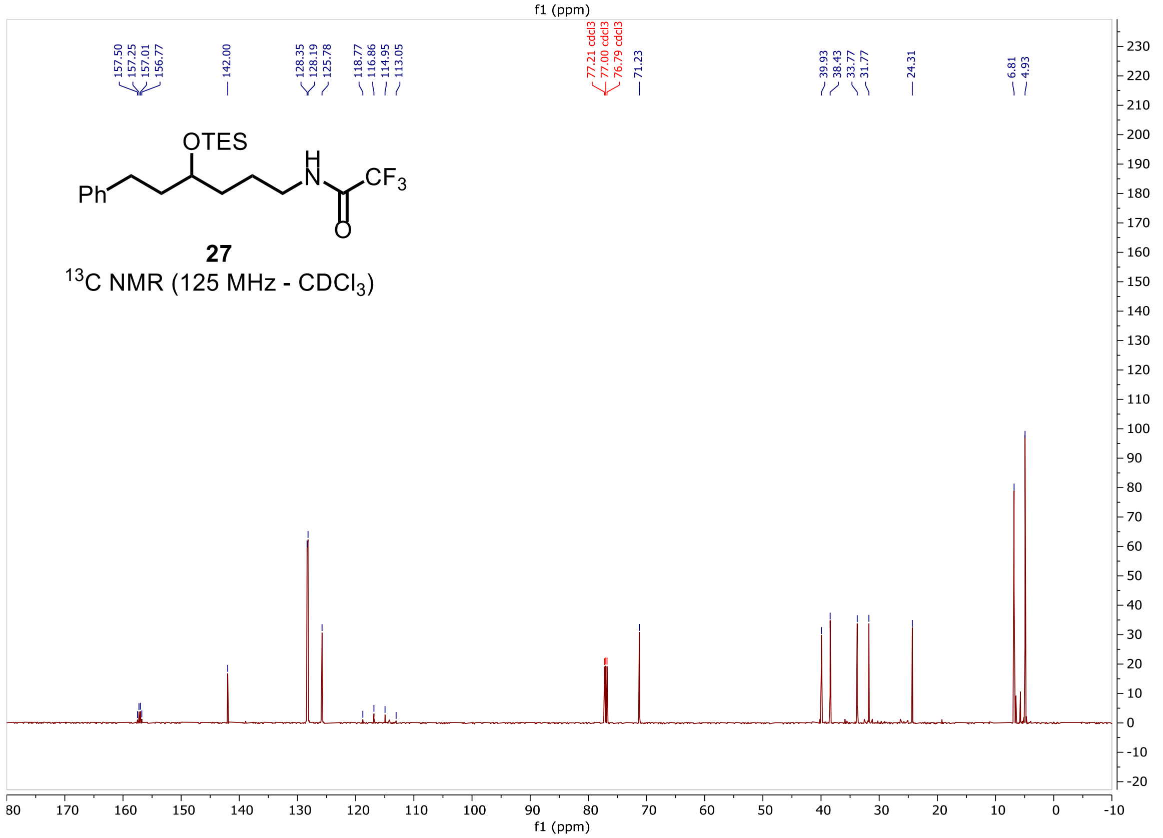
157.50 157.25 157.01 156.77 142.00 128.35 128.19 125.78 118.77 116.86 114.95 113.05
77.21 cdcl3 77.00 cdcl3 76.79 cdcl3 71.23
39.93 38.43 33.77 31.77 24.31 6.81 4.93
OTES
Ph
H
N
CF3
O
27
13C NMR (125 MHz - CDCl3)
f1 (ppm)

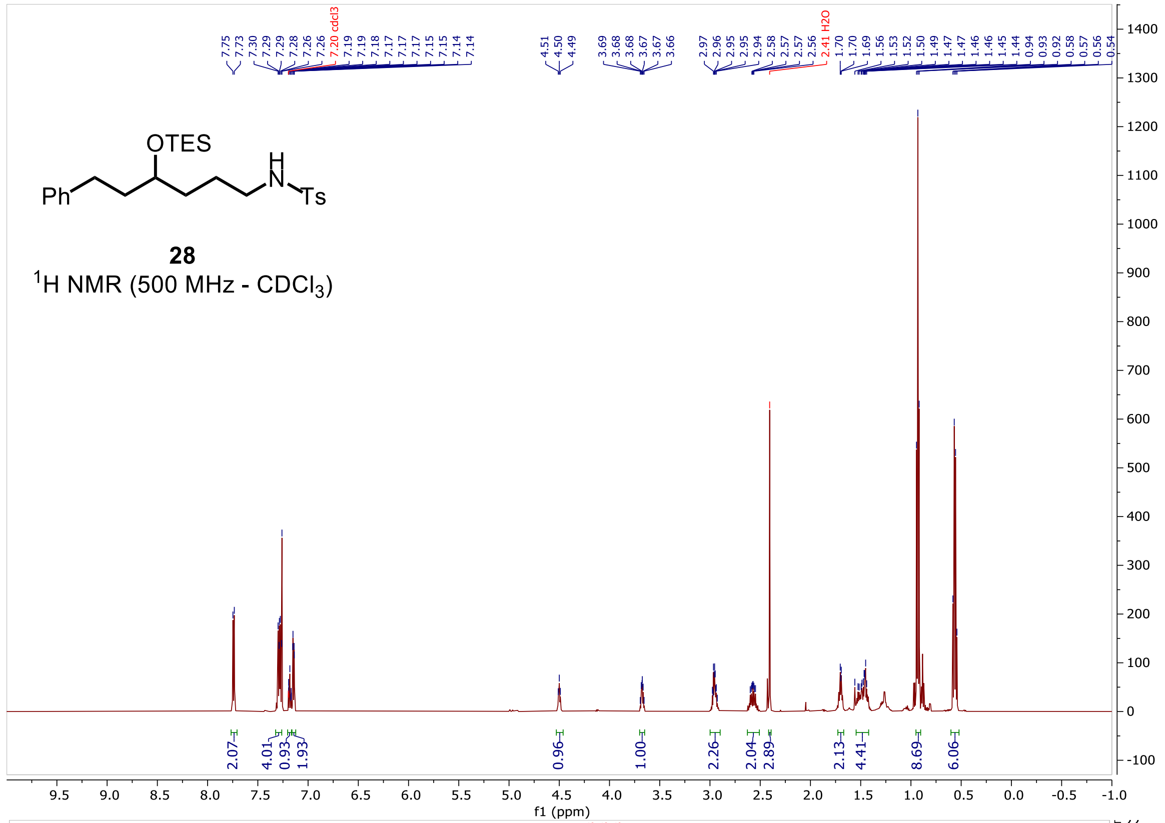
OTES
Ph
H
N
Ts
28
¹H NMR (500 MHz - CDCl3)
f1 (ppm)

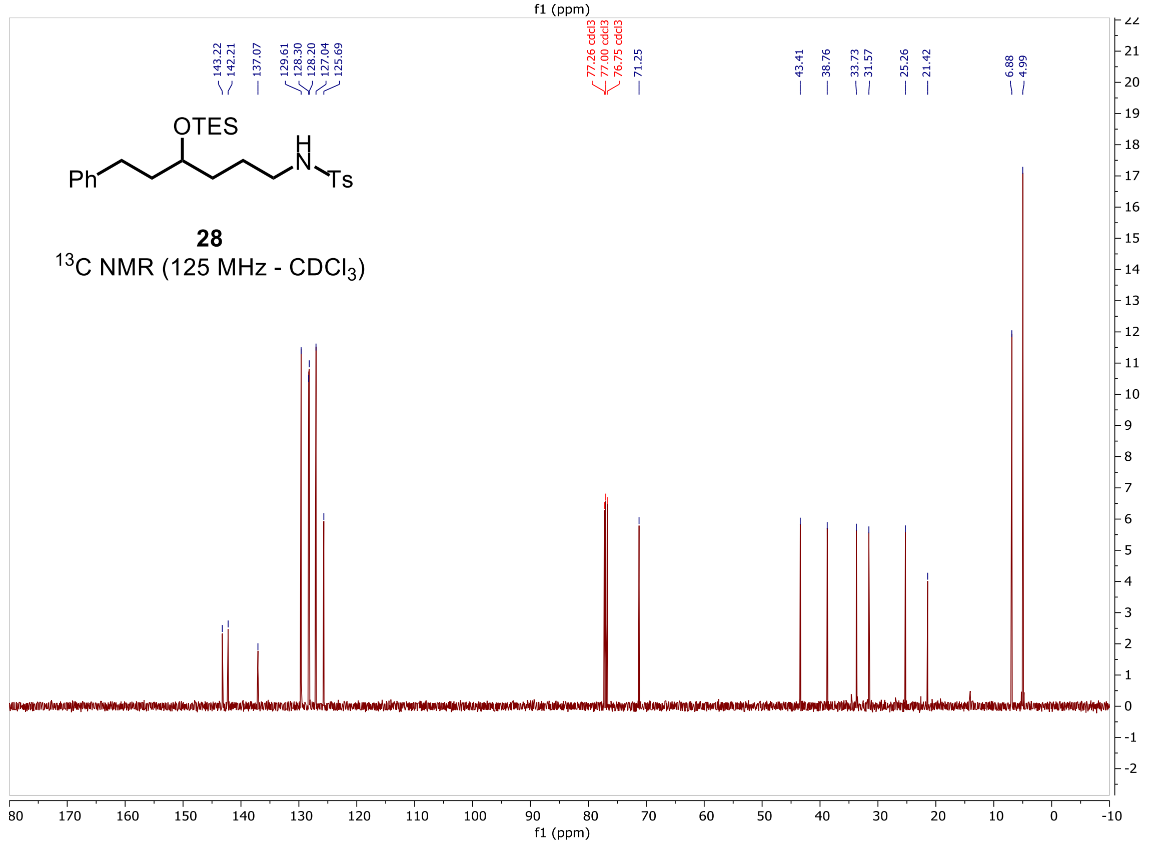
OTES
Ph
H
N
Ts
28
¹³C NMR (125 MHz - CDCl3)
f1 (ppm)

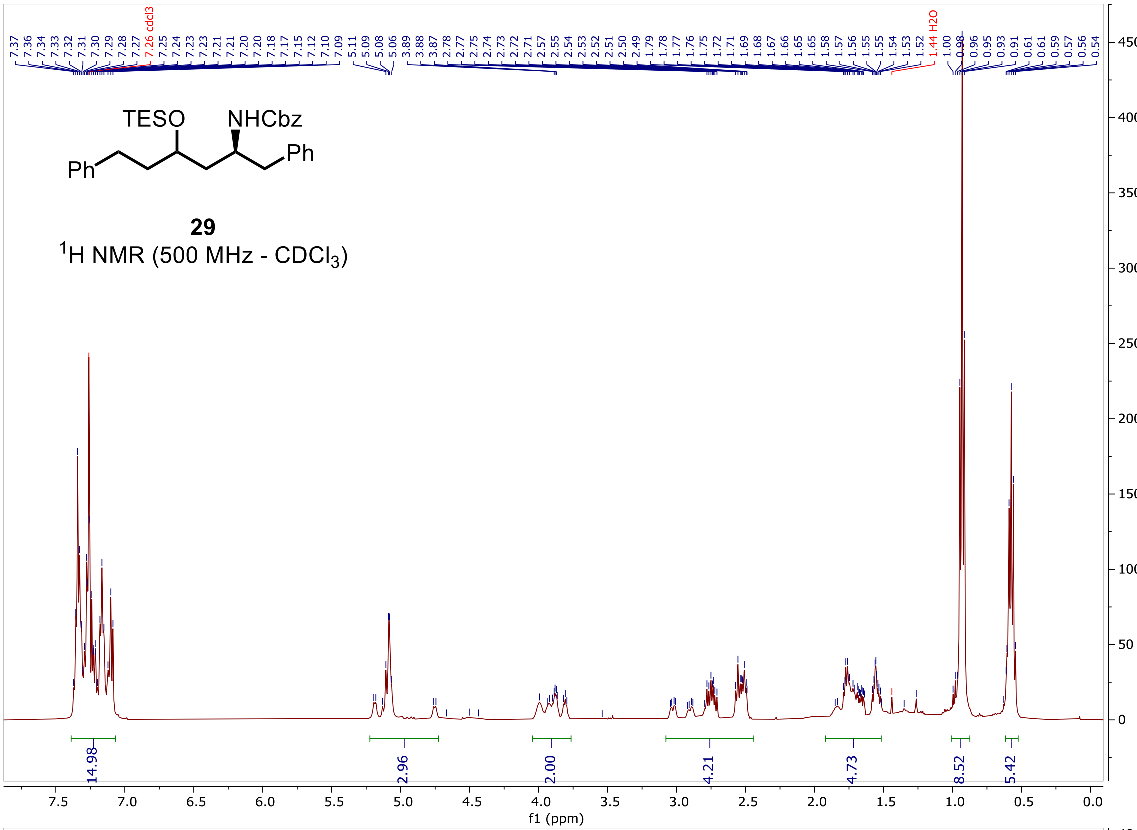
TESO
NHCbz
Ph
Ph
29
1H NMR (500 MHz - CDCl3)
7.26 cdcl3
1.44 H2O
14.98
2.96
2.00
4.21
4.73
8.52
5.42
f1 (ppm)

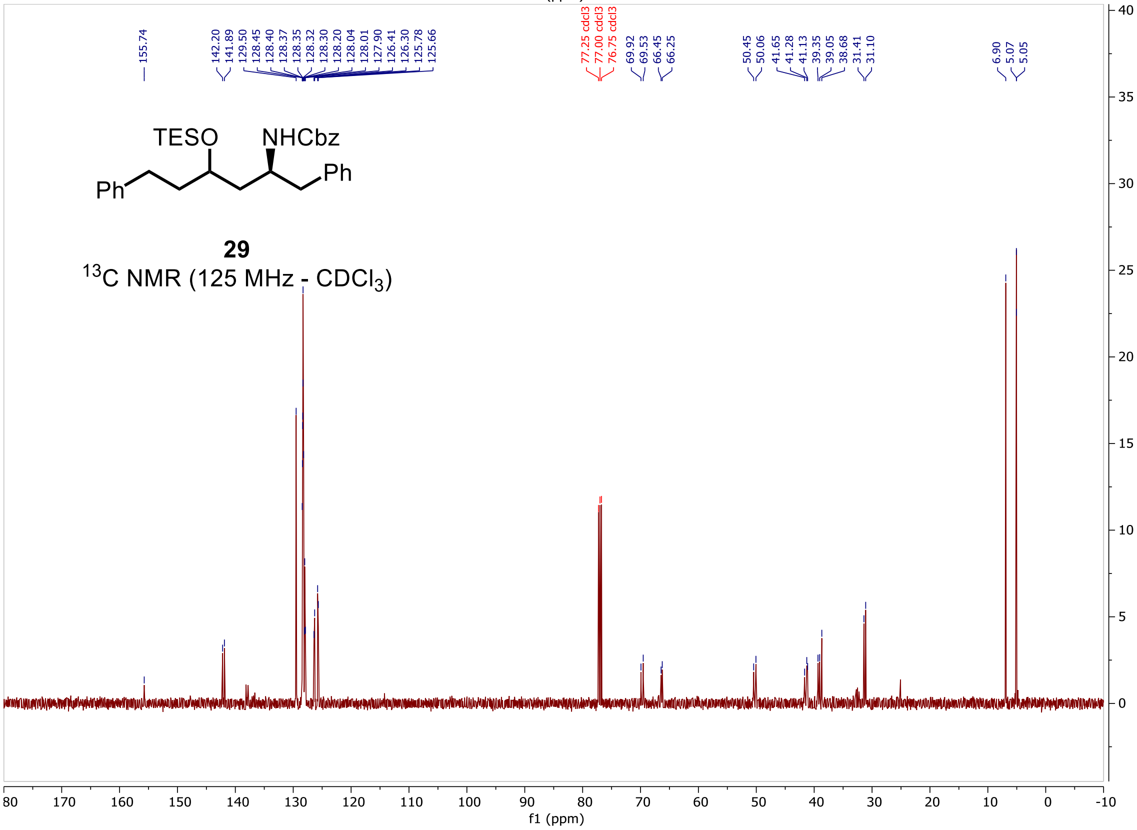
155.74
142.20
141.89
129.50
128.45
128.40
128.37
128.35
128.32
128.30
128.20
128.04
128.01
127.90
126.41
126.30
125.78
125.66
77.25 cdcl3
77.00 cdcl3
76.75 cdcl3
69.92
69.53
66.45
66.25
50.45
50.06
41.65
41.28
41.13
39.35
39.05
38.68
31.41
31.10
6.90
5.07
5.05
TESO
NHCbz
Ph
Ph
29
13C NMR (125 MHz - CDCl3)
f1 (ppm)

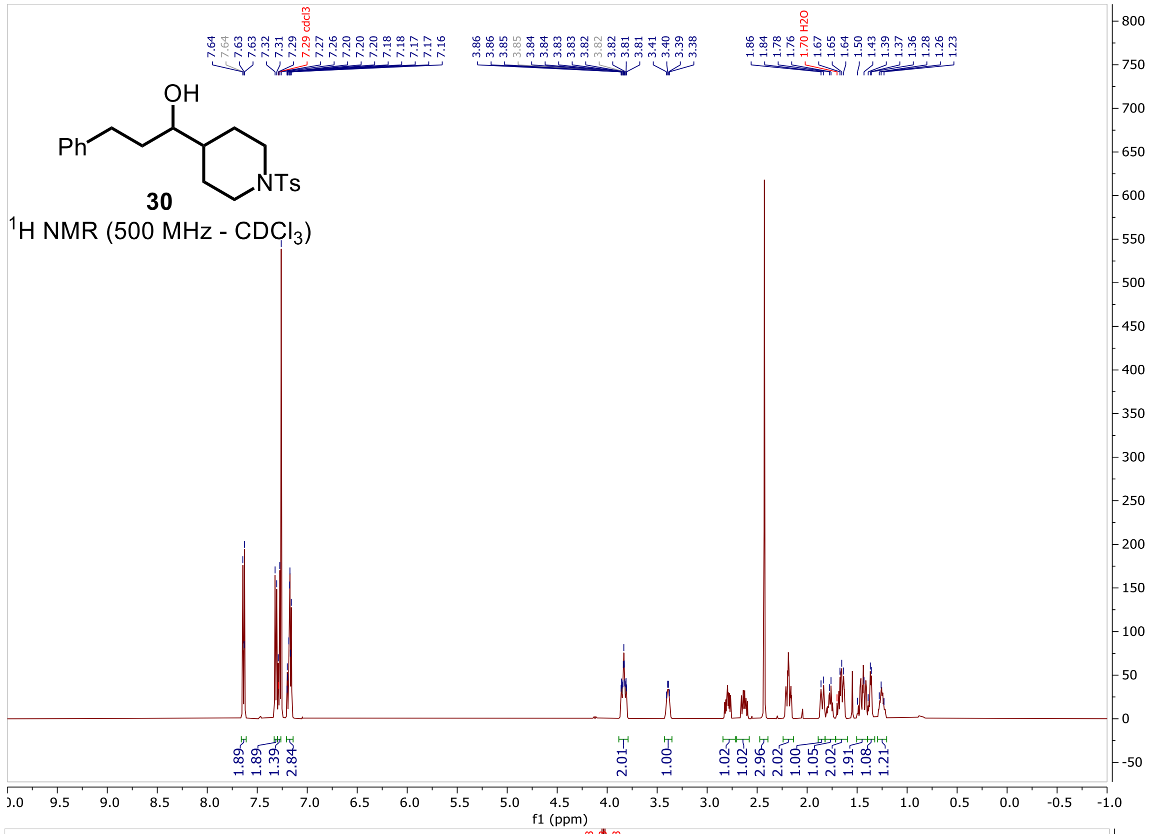

30

^1H NMR (500 MHz - $CDCl_3$)

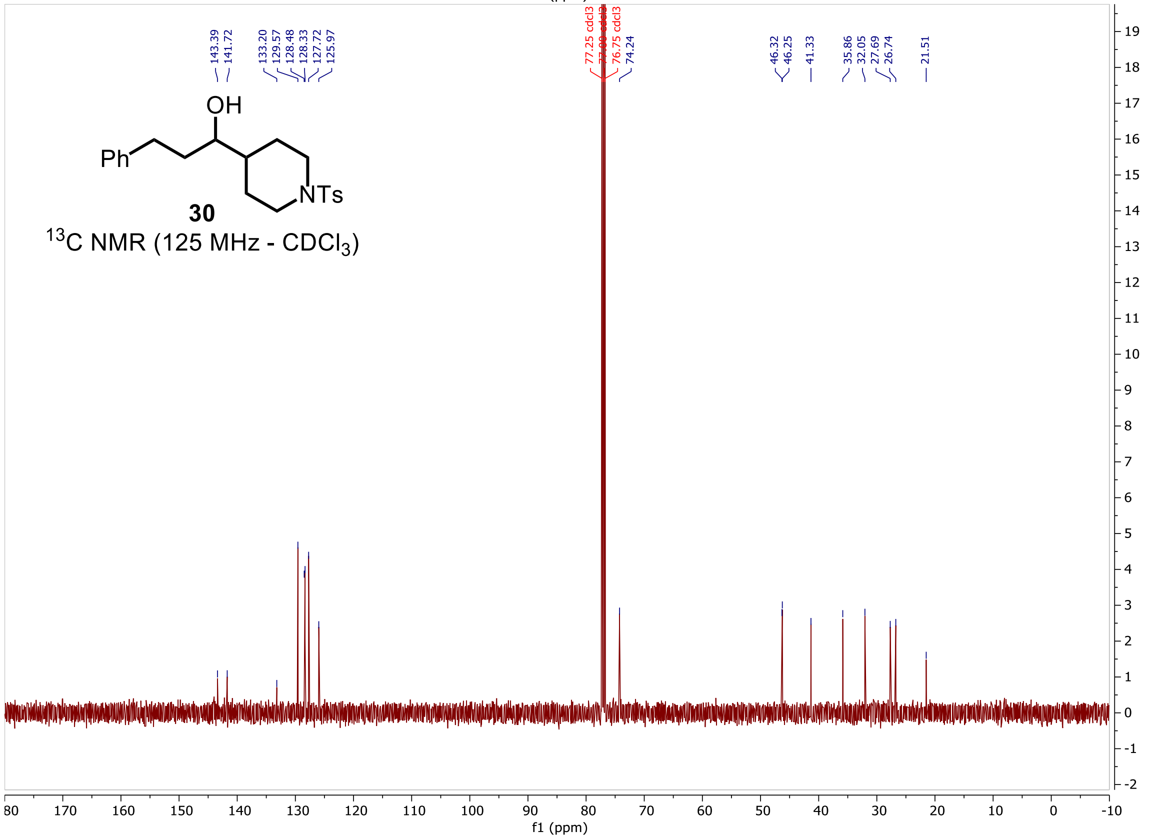

30

^{13}C NMR (125 MHz - $CDCl_3$)

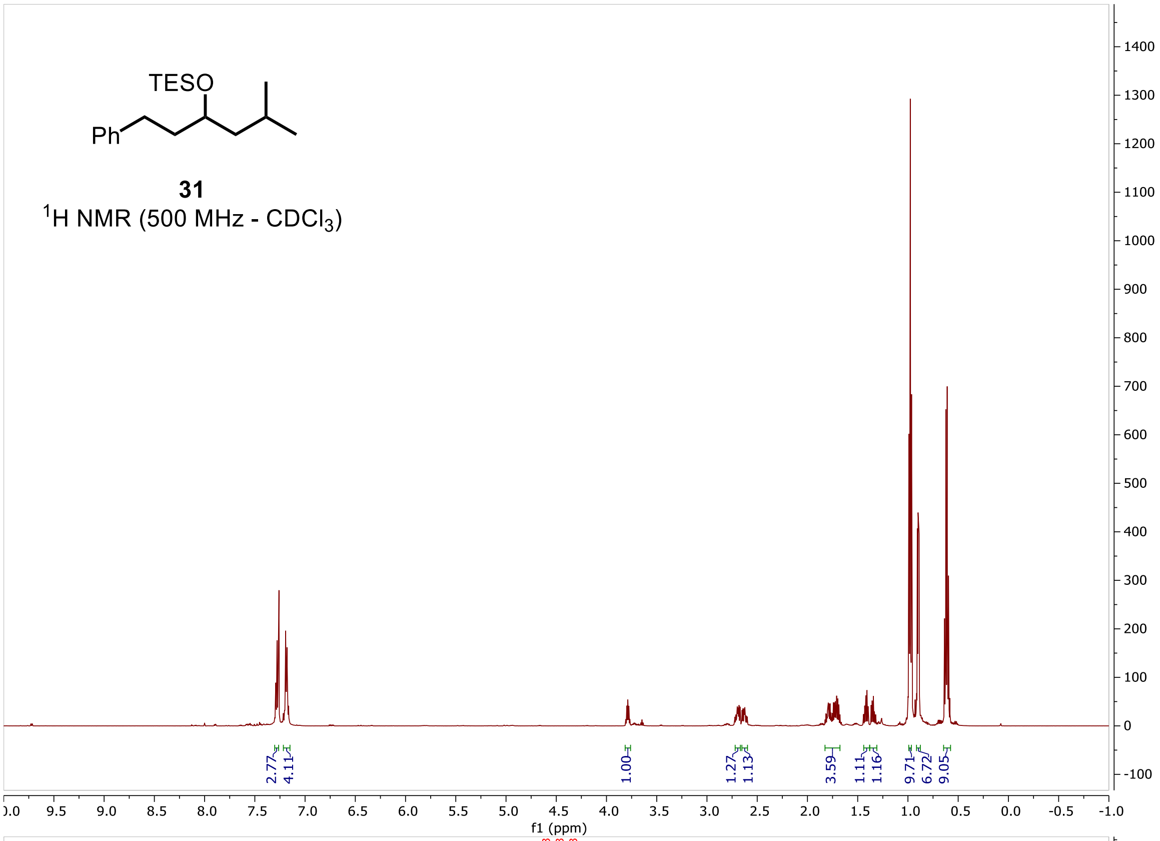
TESO
Ph
31
^{1}H NMR (500 MHz - CDCl$_3$)
2.77
4.11
1.00
1.27
1.13
3.59
1.11
1.16
9.71
6.72
9.05
f1 (ppm)

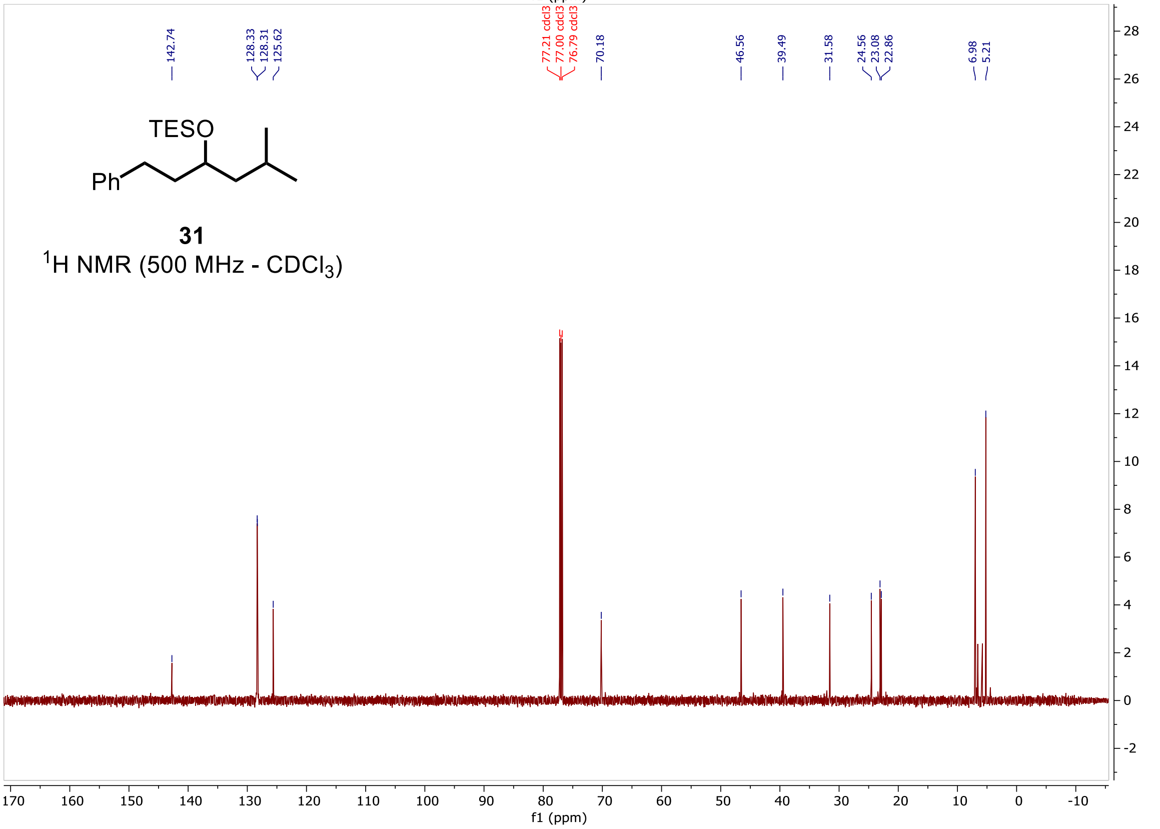
142.74
128.33
128.31
125.62
77.21 cdcl3
77.00 cdcl3
76.79 cdcl3
70.18
46.56
39.49
31.58
24.56
23.08
22.86
6.98
5.21
TESO
Ph
31
^{1}H NMR (500 MHz - CDCl$_3$)
f1 (ppm)

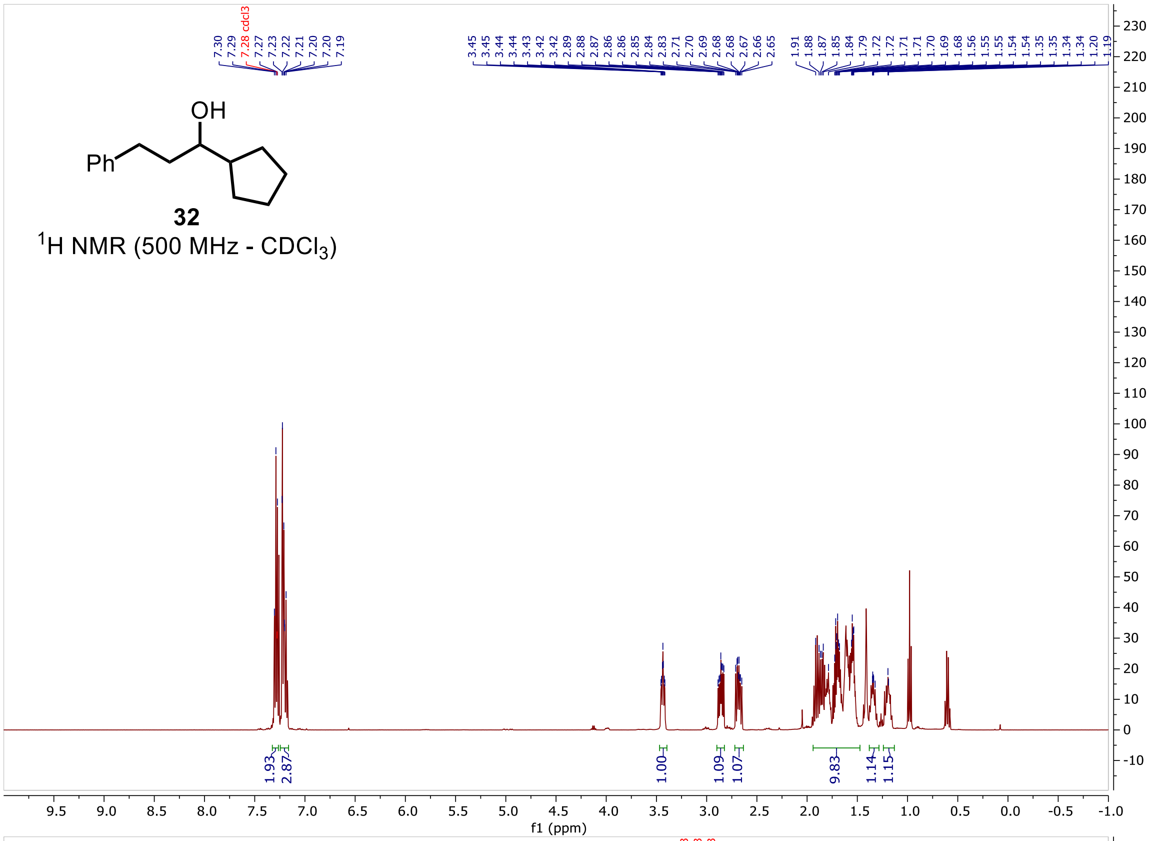
OH
Ph
32
^{1}H NMR (500 MHz - $CDCl_3$)
f1 (ppm)

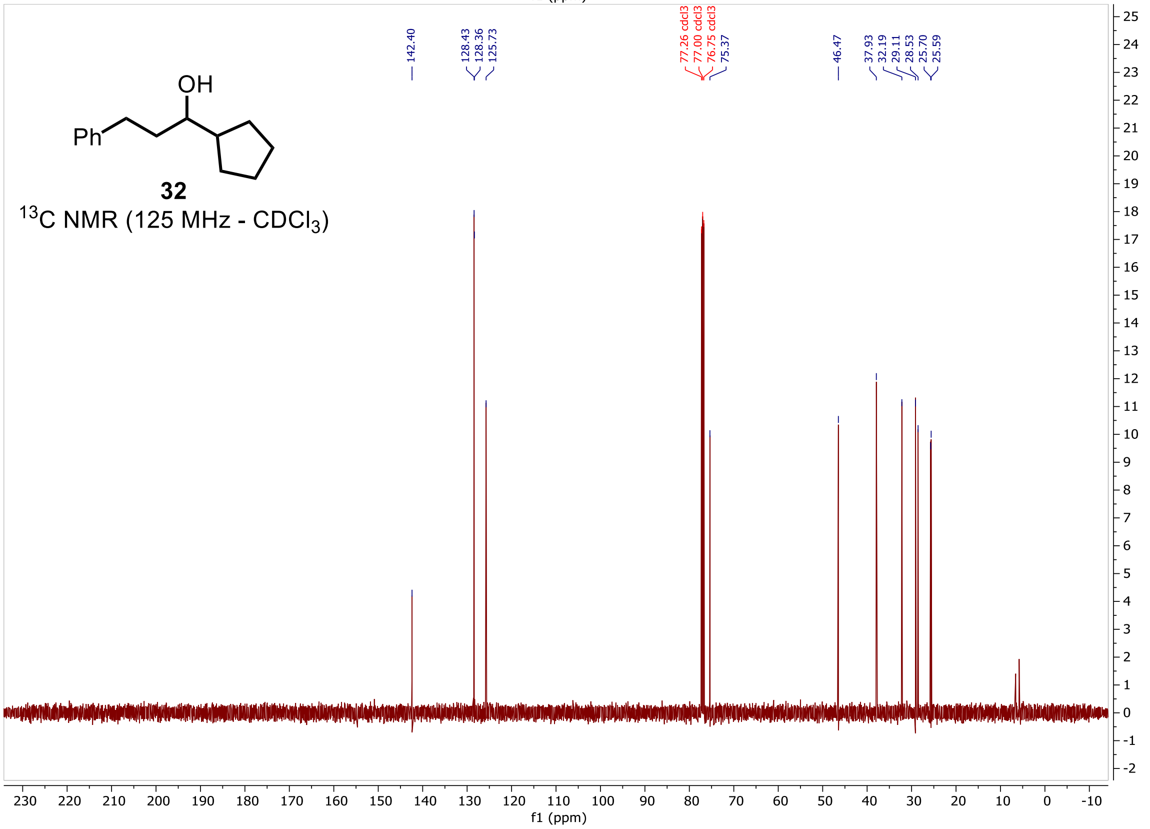
OH
Ph
32
^{13}C NMR (125 MHz - $CDCl_3$)
142.40
128.43
128.36
125.73
77.26 cdcl3
77.00 cdcl3
76.75 cdcl3
75.37
46.47
37.93
32.19
29.11
28.53
25.70
25.59
f1 (ppm)

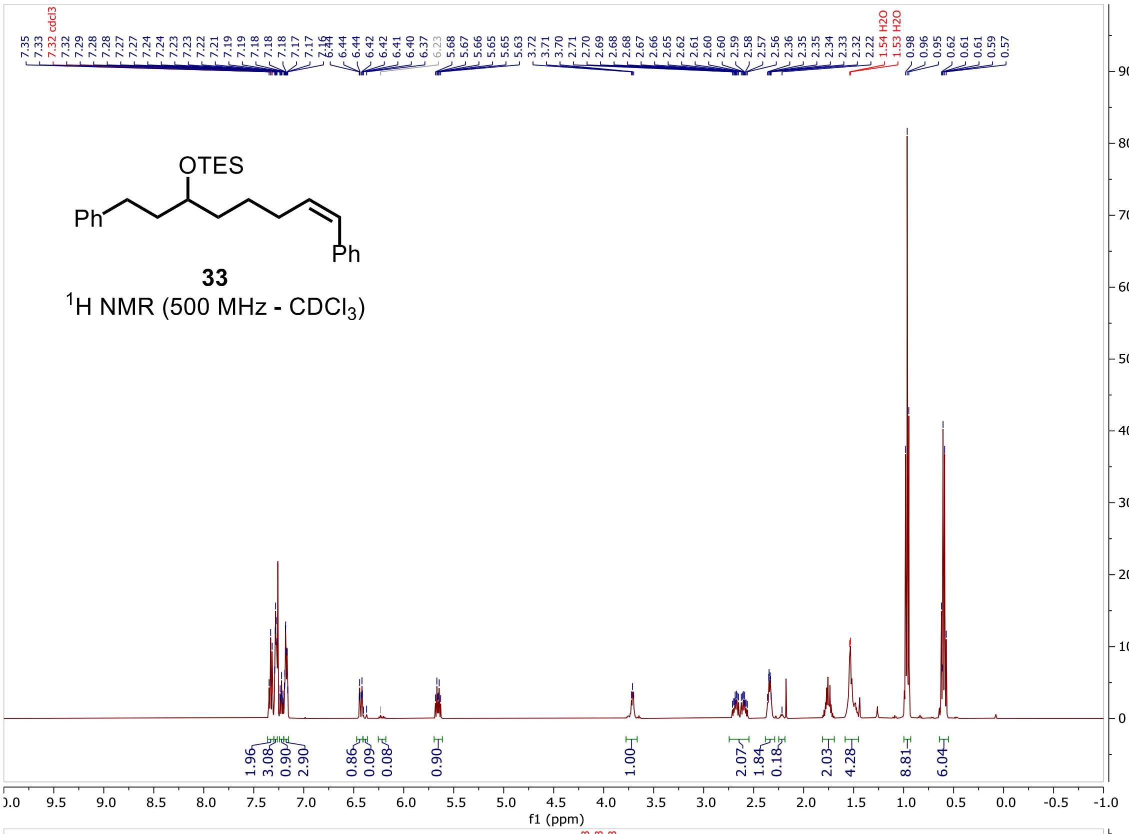
OTES
Ph
Ph
33
^{1}H NMR (500 MHz - CDCl$_3$)
f1 (ppm)

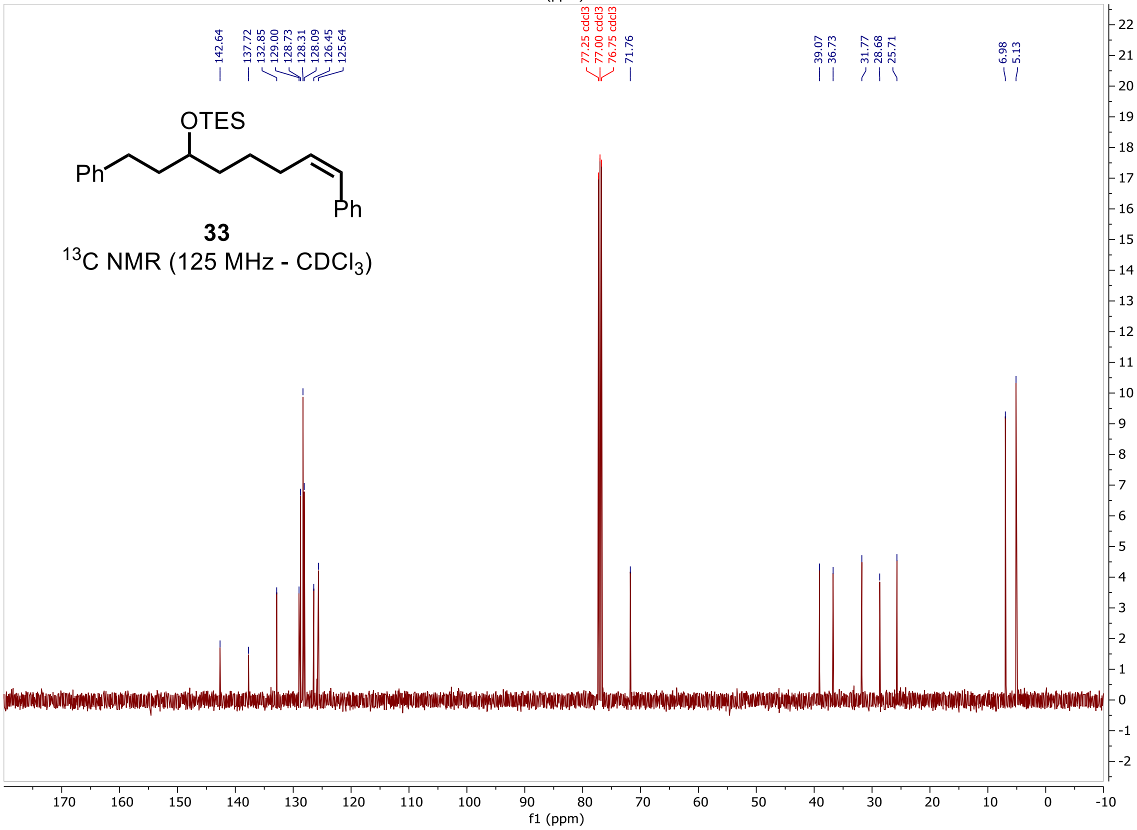
OTES
Ph
Ph
33
^{13}C NMR (125 MHz - CDCl$_3$)
142.64
137.72
132.85
129.00
128.73
128.31
128.09
126.45
125.64
77.25 cdcl3
77.00 cdcl3
76.75 cdcl3
71.76
39.07
36.73
31.77
28.68
25.71
6.98
5.13
f1 (ppm)

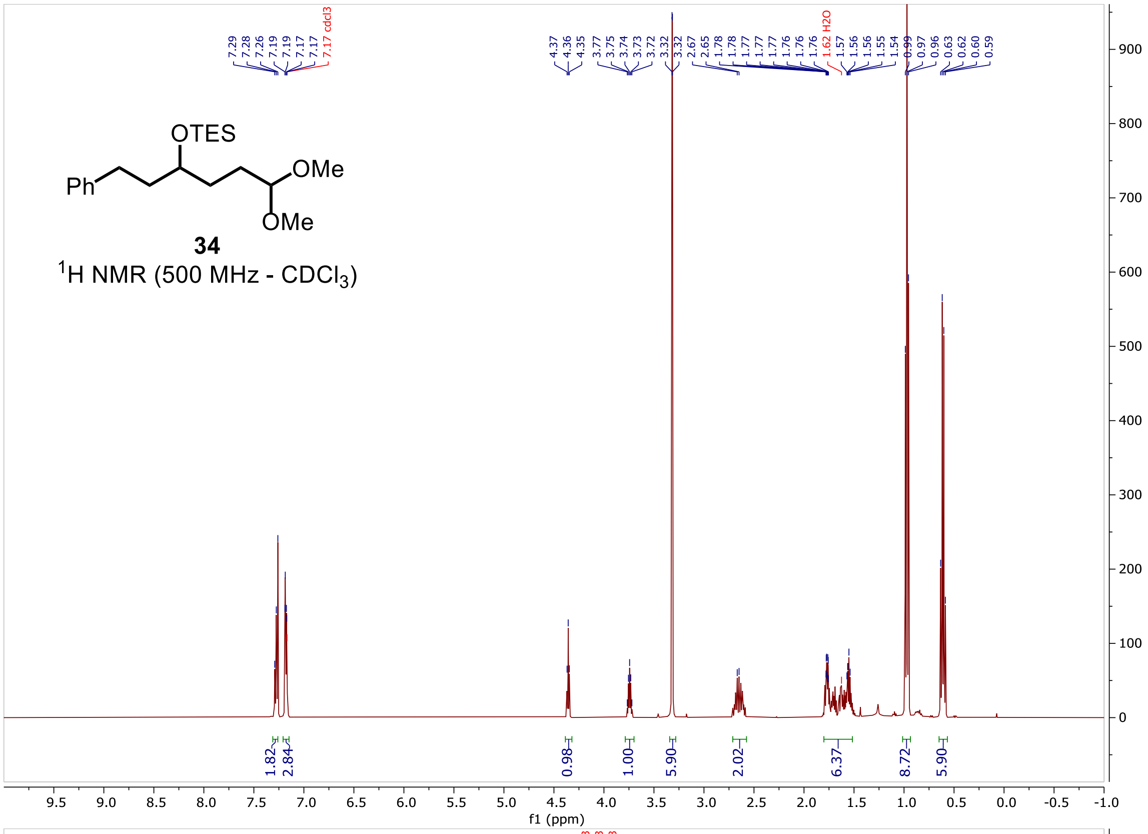
OTES
Ph
OMe
OMe
34
^{1}H NMR (500 MHz - CDCl$_3$)
f1 (ppm)

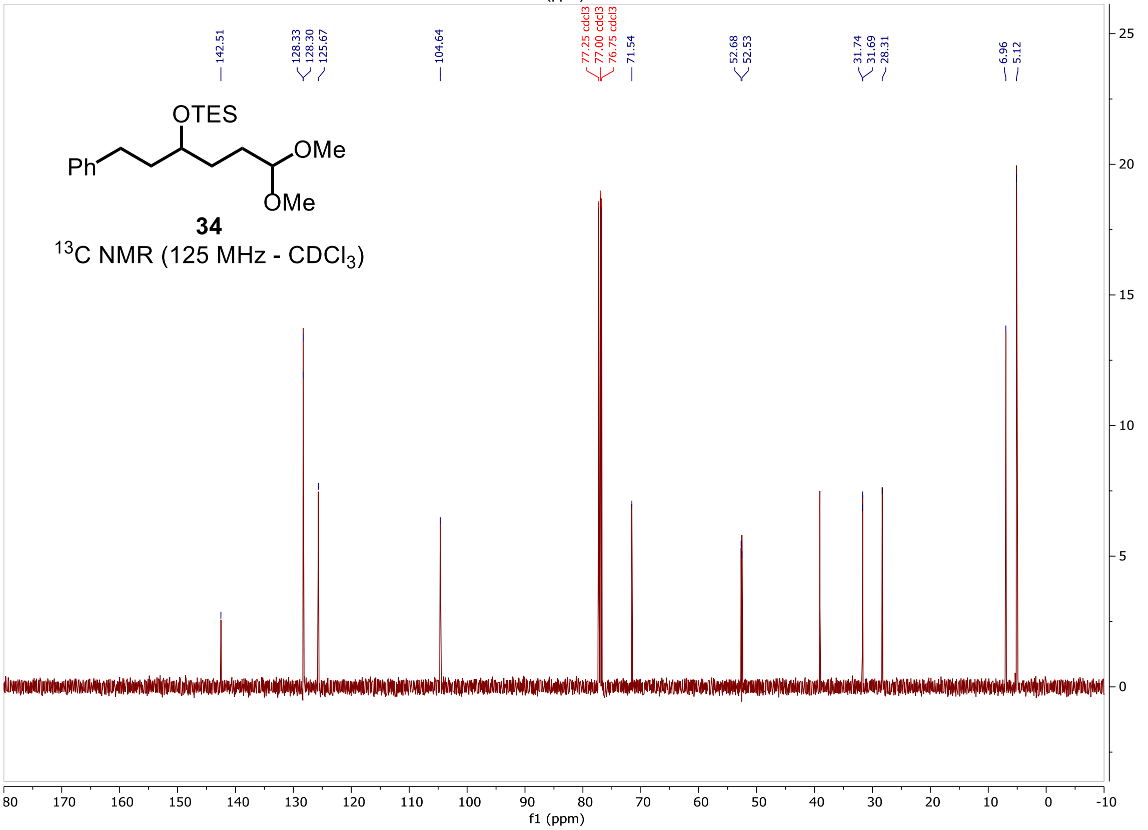
OTES
Ph
OMe
OMe
34
^{13}C NMR (125 MHz - CDCl$_3$)
f1 (ppm)

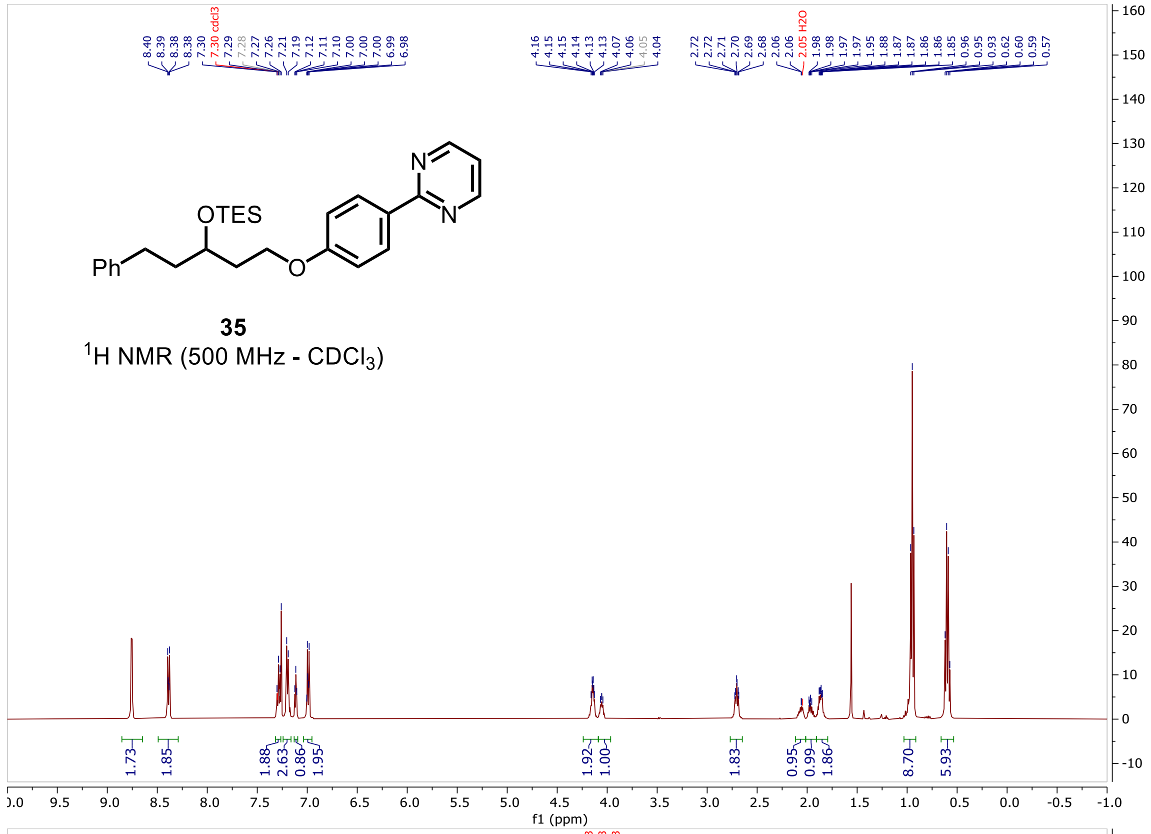
8.40 8.39 8.38 8.38 7.30 7.30 cdcl3 7.29 7.28 7.27 7.26 7.21 7.19 7.12 7.11 7.10 7.00 7.00 7.00 6.99 6.98
4.16 4.15 4.15 4.14 4.13 4.13 4.07 4.06 4.05 4.04
2.72 2.72 2.71 2.70 2.69 2.68 2.06 2.06 2.05 H2O 1.98 1.98 1.97 1.97 1.95 1.88 1.87 1.87 1.86 1.85 0.96 0.95 0.93 0.62 0.60 0.59 0.57
OTES
Ph
O
N
N
35
1H NMR (500 MHz - CDCl3)
1.73 1.85 1.88 2.63 0.86 1.95 1.92 1.00 1.83 0.95 0.99 1.86 8.70 5.93
f1 (ppm)

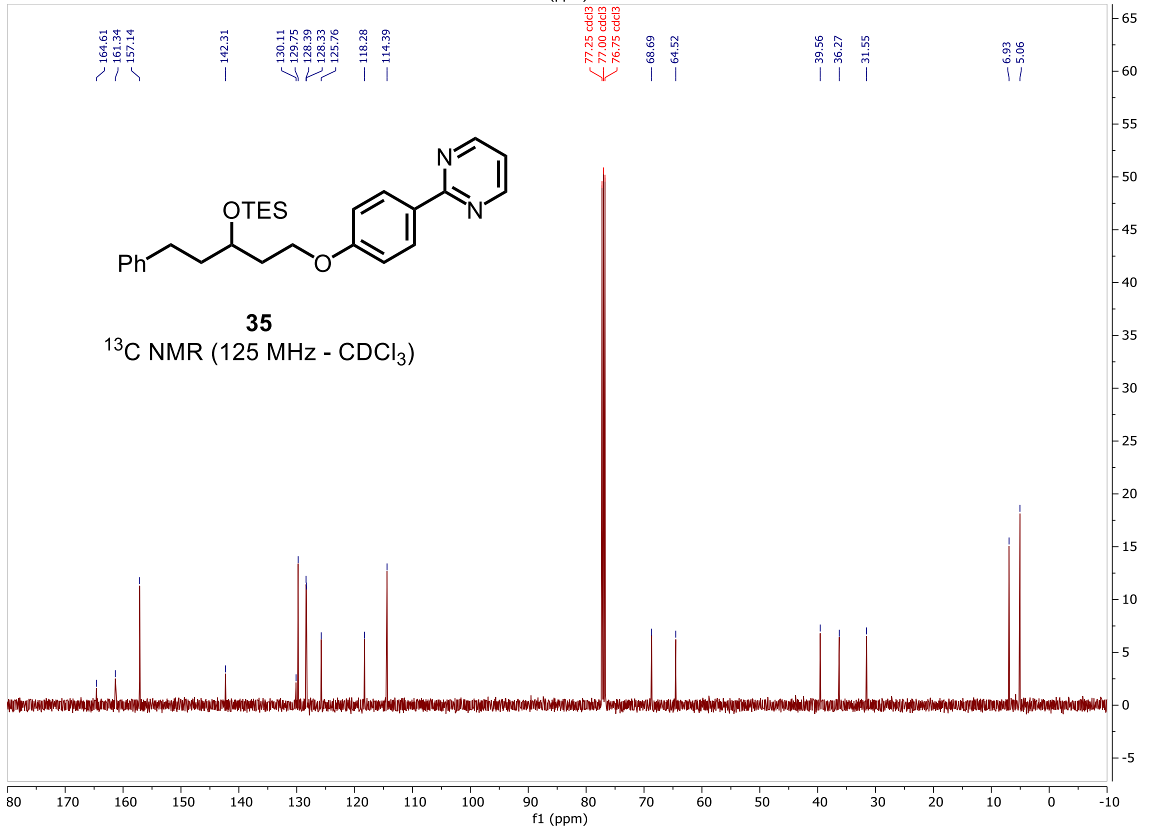
164.61 161.34 157.14 142.31 130.11 129.75 128.39 128.33 125.76 118.28 114.39
77.25 cdcl3 77.00 cdcl3 76.75 cdcl3 68.69 64.52 39.56 36.27 31.55 6.93 5.06
OTES
Ph
O
N
N
35
13C NMR (125 MHz - CDCl3)
f1 (ppm)

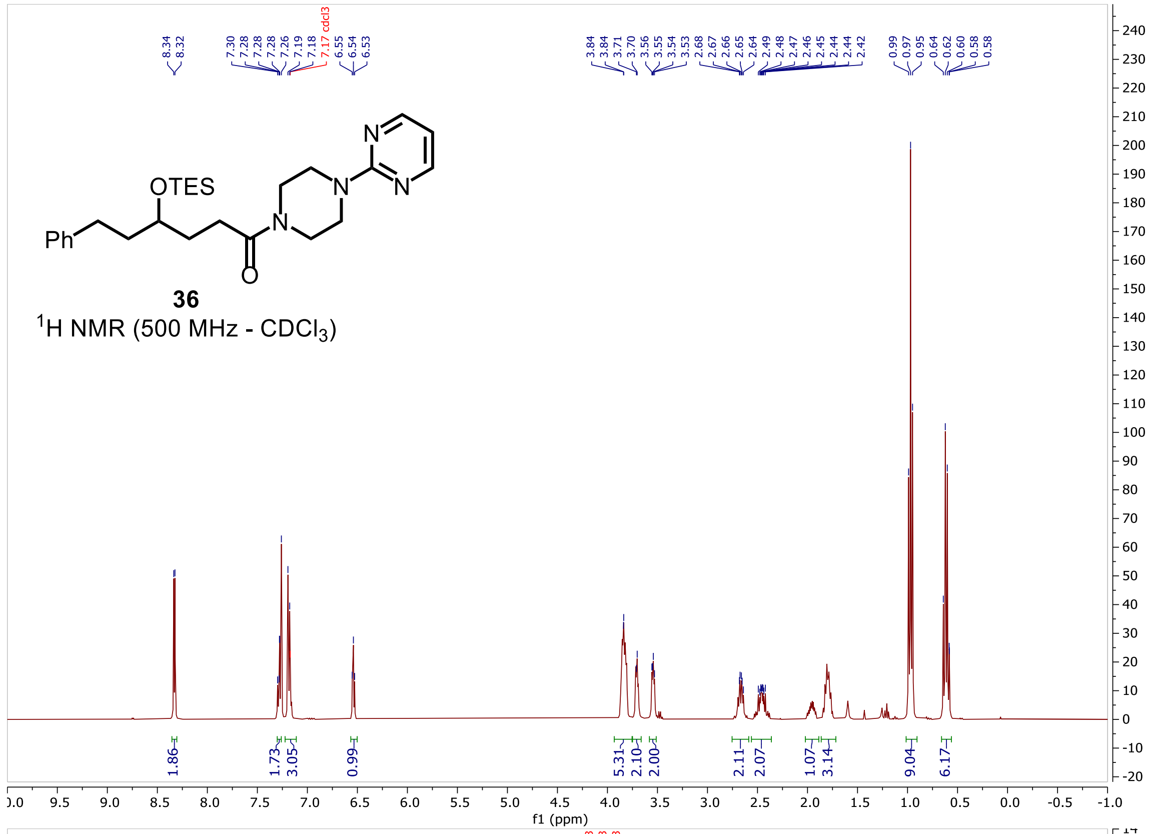
OTES
Ph
N
36
1H NMR (500 MHz - CDCl3)
f1 (ppm)

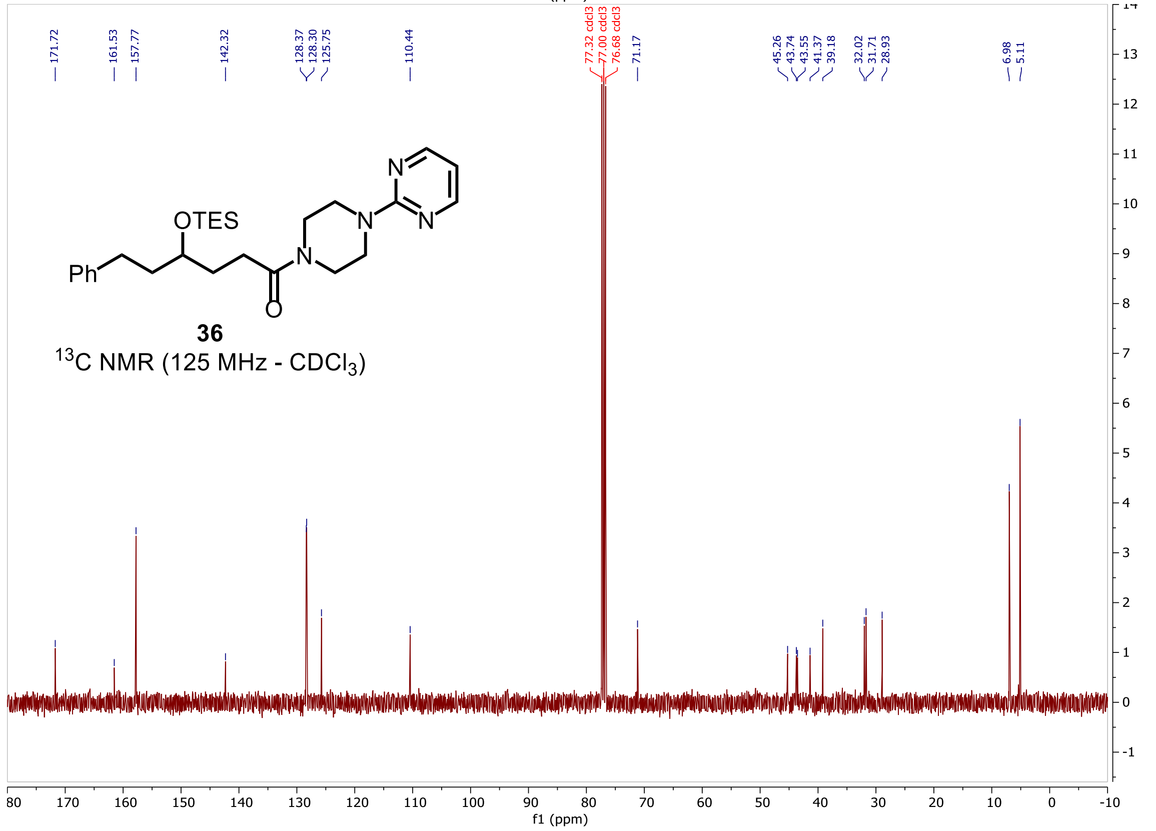
OTES
Ph
N
36
13C NMR (125 MHz - CDCl3)
f1 (ppm)
